# Quantum generators of random numbers

**DOI:** 10.1038/s41598-021-95388-7

**Published:** 2021-08-09

**Authors:** Marcin M. Jacak, Piotr Jóźwiak, Jakub Niemczuk, Janusz E. Jacak

**Affiliations:** 1 Wrocław University of Science and Technology, Wyb. Wyspiańskiego 27, 50-370 Wrocław, Poland; 2 Faculty of Computer Science, Wrocław University of Science and Technology, Wyb. Wyspiańskiego 27, 50-370 Wrocław, Poland; 3 Department of Quantum Technologies, Wrocław University of Science and Technology, Wyb. Wyspiańskiego 27, 50-370 Wrocław, Poland

**Keywords:** Quantum information, Information theory and computation

## Abstract

Generation of random numbers is a central problem for many applications in the field of information processing, including, e.g., cryptography, in classical and quantum regime, but also mathematical modeling, Monte Carlo methods, gambling and many others. Both, the quality of the randomness and efficiency of the random numbers generation process are crucial for the most of these applications. Software produced pseudorandom bit sequences, though sufficiently quick, do not fulfill required randomness quality demands. Hence, the physical hardware methods are intensively developed to generate truly random number sequences for information processing and electronic security application. In the present paper we discuss the idea of the quantum random number generators. We also present a variety of tests utilized to assess the quality of randomness of generated bit sequences. In the experimental part we apply such tests to assess and compare two quantum random number generators, PQ4000KSI (of company ComScire US) and JUR01 (constructed in Wroclaw University of Science and Technology upon the project of The National Center for Research and Development) as well as a pseudorandom generator from the Mathematica Wolfram package. Finally, we present our new prototype of fully operative miniaturized quantum random generator JUR02 producing a random bit sequence with velocity of 1 Mb/s, which successfully passed standard tests of randomness quality (like NIST and Dieharder tests). We also shortly discuss our former concept of an entanglement-based quantum random number generator protocol with unconditionally secure public randomness verification.

## Introduction

The turn of the 20th and 21st centuries can be considered the beginning of the currently observed rapid development and spreading of information technology in almost all areas of economy and science and in the sphere of utility. Information technology in many key aspects requires taking into account in algorithms the generating of random variables. Hence, the problem of random number generators plays a fundamental role in the field of information technology, in particular, of information security.

The current applications of random number generators (RNGs) extend to the area of information technology in terms of:

- applications in the field of cryptography—for individual user applications;—for generation of random initialization sequences (so-called seeds) for encryption algorithms, authentication or digital signature;—for key generation (for asymmetric and symmetric cryptography, e.g., for the One Time-Pad cipher^1^ to ensure unconditional security), nonces/initiating vectors (IV), challenges for authentication, selection of exponents in the Diffie-Hellman protocol
- other IT applications: e.g., tags/tokens for communication protocols, for indexing in databases, etc.
- statistical applications (e.g., selection of a representative sample for statistical analysis)
- numerical simulations of the Monte Carlo type
- nondeterministic behavior of artificial intelligence (AI)—AI in computer games, in self-controlled devices (e.g., drones), etc.
- AI algorithms: neural networks (e.g., random weighting for networks) and genetic algorithms (e.g., randomly introducing mutations, randomly mixing representatives)
- structures and support services of currently popular cryptocurrencies (e.g., bitcoin wallets, bitcoin exchanges, etc.)
- games of chance (e.g., online casinos, also for cryptocurrencies)
- randomness in control processes (important problem of sample selection for control processes quality)
- randomness in administration (e.g., drawing the order on election lists)

The above list briefly shows the scale of the range of the application of randomness and of random number generators. In this context, the quality of randomness and its truthfulness become a fundamental problem.

The consequences of the predictability of the generated classical pseudorandom sequences are obvious—the lack of a true randomness in any of the previously indicated applications is an obstacle to the intended functioning. In the case of cryptographic applications, the consequences can be particularly severe. The problem with classic random number generators, i.e., pseudorandom number generators, consists in the possibility to know the deterministic process of pseudorandom generation by unwanted persons. This may result, in the case of cryptography, in compromising a myth of security. Another problem may be the incorrect handling of the generated sequence—mostly in cryptographic uses, the generated random sequence is applied once. Its multiple usage may lead to a security breach (e.g., in the case of the OTP cipher, a sufficiently long key should be truly random and used once in that protocol, otherwise it will be possible to break the code). The scale of threat can be illustrated by selected attacks and information about threats as listed below:

- 2006–2012—over the years there have been many reports of attacks on cryptographic keys generated by weak PRNGs (which allows for example to carry out a brute force attack on SSH secured with RSA keys)^2,3^
- 2010—a spectacular attack was carried out on users of Sony’s PlayStation 3 (PS3) game console (data was stolen as many as 77 million users). The attack was carried out using a flaw in the implementation of the ECDSA algorithm by Sony (disclosed materials reported that the same random number was mistakenly used multiple times as the so-called nonce for authentication)^4^
- 2012—two groups of researchers revealed numerous RSA encryption keys that were then actively used on the Internet as secure and were at risk of being broken due to insufficient random generator that was used to create them^5^
- 2013—following Snowden’s disclosure of these shortcomings to the U.S. National Security Agency (NSA), Reuters^6^ and New York Times^7^ conducted investigations revealing that the NSA was intentionally secretly lowered the security of the world’s popular hardware and programming solutions for the purpose of crypto-attacks on encrypted content (including attacks on RNGs):
  - Dual EC DRBG (Dual Elliptic Curve Deterministic Random Bit Generator) was used for this, a PRNG created and strongly pushed as a standard by the NSA. Only in 2013 it turned out that the NSA was the only one to have a backdoor for this generator and thanks to this the NSA was able to crack the cryptographic keys that had been generated using these generators. Upon disclosure, RSA Security and the US National Institute of Standards and Technology (NIST) instructed not to use the Dual EC DRBG generator.
  - NSA carried out a secret project code-named Bullrun, focusing on exploiting vulnerabilities in a disseminated PRNG, to which it had access at random, in various devices (e.g., Juniper).
  - Intel and Via on-chip HRNG motherboard random number generators probably also had backdoors^8^. It has been indicated that the RdRand and Padlock instructions most likely have backdoors in Linux kernels up to v 3.13.
  - Suspected scandal over NSA^9^ eavesdropping of 35-country leaders was just related to the use of attacks on RNG.
- 2013—Google confirmed that the IBM Java SecureRandom class in Java Cryptography Architecture (JCA) generated repetitive (and therefore predictable) sequences, which compromised application security made for Android to support the electronic currency Bitcoin – the equivalent of USD large amount in Bitcoins^10,11^ was stolen.
- 2014—It is suspected that the attack on the Tokyo cryptocurrency exchange MtGox, in which more than 800,000 Bitcoins were stolen (which resulted in the declaration of bankruptcy by MtGox) was related to an attack on RNG^12^
- 2015—Hard-to-detect remote attack using an externally attached hardware Trojan horse on FPGA-based TRNGs presented^13^
- 2015—theft of 18,866 bitcoins from the Bitstamp exchange (12% of the currency traded on this exchange) – attack signature of the RNG attack^14^
- 2017—ANSI x9.31 PRNG compliant to 2016 FIPS USA (Federal Information Processing Standards) – compromised if used with hard-coded seed (DUHK attack—Don’t Use Hard-coded Keys)^15^

The presented above examples clearly show that classic random number generators may be exposed to various attacks, or may have the so-called backdoors. This justifies the need to develop alternative technologies that could replace the classic generators on a large scale. The most promising, because they have a fundamental justification for the randomness in the formalism of quantum mechanics, are quantum random number generators.

Classical random number generators, due to the deterministic generation process (dictated by deterministic laws of classical physics or deterministic mathematical information algorithms), generate sequences which, despite the perfect balance between the digits 0 and 1, will inevitably always be characterized by the presence of certain deterministic long-range patterns – correlations that can pose a potential risk to IT security, unexpected errors in scientific simulations or gaps in a physical processes testing^16–18^.

It should also be emphasized that regardless of the reduction of the above-mentioned threats (e.g., using randomness tests to detect repetitive patterns, adequate security of the generation process, one-time use of the generated sequences), there is a certain threat, which will not be able to handle it within classical computer science—it is a quantum computer. The appearance of an efficient quantum computer (currently pseudo-quantum computers are being commercialized, e.g., DWave^19^ significantly exceeding the computing power of classic devices^20^, moreover Google presented recently the fully operational quantum machine Sycamore to demonstrate Quantum Supremacy^21^, and later Chinese scientists presented a photonic quantum computer, called Jiuzhang^22^) will cause any classical random number generator to be potentially endangered—theoretically, a quantum computer will find the deterministic nature of the generation process in a real time, as long as this process is based on the phenomenon of classical physics. The answer to this threat seems to be quantum random number generators, which are becoming more and more popular, despite the fact that the prospect of an efficient large scalable entanglement-based quantum computer is still postponed due to current technological constraints.

## Types of random number generators

There are many types of random number generators. They can be divided in relation to e.g. the type of the generation process—software random number generators (software RNGs—based on the deterministic software) or hardware RNGs (based on the physical phenomenon—classical or quantum). Different division is based on the physical nature of the generation process—-classical RNGs and quantum RNGs. These two main divisions’ perspectives partly overlap—software RNGs are purely classical, while hardware RNGs are divided into classical, quantum, and generators, in which it is impossible to clearly distinguish the nature of the physical process.

There are further divisions within subcategories, e.g., there are different types of pseudorandom number generators (PRNGs), among which there are currenty cryptographically secure pseudorandom number generators (CSPRNGs). Classical hardware RNGs can be divided due to a specific physical process underlying the generation, similarly to quantum RNGs. Some generators may additionally test the generated sequences basing on the implemented tests and assessing the deviation from the assumed randomness parameters of the generated sequence. There are also hybrid generators which combine features of many categories.

The basic subgroup of PRNG are algorithmic random number generators – these generators use an algorithmic process of random sequence generation based on a preliminary random key (initial entropy portion). The initialization key represents a portion of entropy that remains unchanged no matter how long the generated sequence takes or how complex it is. Therefore, the software RNGs are undoubtedly pseudorandom. The knowledge of the initial random seed compromises the security and the randomness of the entire generated sequence—based on the knowledge of the initial key and algorithm parameters, it is possible to recreate the entire generated sequence. In such a case, the sequences generated by PRNG (when the initial random seed is compromised) are repeated and remain deterministic, resulting with the generation process as no longer efficient.

Classical hardware random number generators do not require an initial entropy—in this case, the source of entropy is a classical physical process. If the available entropy is consumed, such generator must wait until the generation process supplies enough portion of a new entropy. Generators of this class are also pseudorandom generators due to the determinism of the classical physics, and therefore can be a potential target of an attack. In particular, an effective attack on such a generator could be carried out using a quantum computer.

Quantum hardware random number generators, or quantum random number generators (QRNG), can be divided into three categories^23^:

- Practical Quantum Random Number Generators—fully trusted and calibrated devices. The randomness depends on the correct modeling and implementation of the physical quantum process. Typically, the generation speed is moderate and the cost of the device relatively low. In practice, in these devices, quantum randomness is often mixed with classical noise (which, however, can be removed if the basic quantum process is modeled appropriately). For these devices, security depends on trust in the device and its components, what can be a problem when dealing with third-party vendors.
- Self Testing Quantum Random Number Generators—the generated sequence is tested for randomness because of limited confidence in the implementation of a physical process. Testing can be based on classic tests, but also on e.g., verification of the existence of quantum entanglement, by checking the Bell inequalities^24^. These devices are also known as device independent quantum random number generators^25^. Due to the complexity of the testing process, such generators are usually slow or require additional complex testing devices.
- Semi-testing quantum random number generators—this category includes devices in which the randomness testing has been reduced by virtue of the implementation confidence. This allows for an optimalization of the speed parameters with the cost of confidence in the generated randomness. Some components in such devices are considered safe and trusted due to their precise characterization, others cannot be considered as such, and therefore it is necessary to perform more extended tests.

## Randomness definition

Many definitions of the randomness have been developed, along with many different concepts of testing it. One of the basic concepts of randomness was given by Kolmogorov in the 1950s—it was based on the computational complexity. In this approach (the so-called Kolmogorov complexity), the generated sequence is random if it is of high Kolmogorov complexity^26^. This definition of randomness, similarly to others (described briefly below), turns out to be incomplete in the sense that it will always be possible to prepare a deterministic generator that will generate a predictable sequence, and yet it will pass all the proposed statistical tests as defined (as there is uncountably many different infinite bit sequences thus the complete set of tests should also be uncountably infinite, which makes it impossible to be properly described). Therefore, the natural randomness contained in the laws of physics on which to generate unpredictable truly random numbers is sought more and more frequently.

### Classical approach to the randomness

The definition of the randomness presents a major conceptual difficulty within probabilistic and statistical theories. While there are general formalized definitions of the randomness associated with the developed mathematical-statistical apparatus, they are unable to provide a complete formal description of the unpredictable true randomness.

A detailed discussion of the theoretical foundations of the classical concept of the randomness can be found in the Supplementary Information A.

However, irrespective of the assumed theoretical approach, any of them does not seem to constitute a complete condition of the randomness. The concept of fundamental unpredictability seems to be closer to the essence of the randomness, despite the imperfections of formal attempts to formulate an appropriate description.

According to the arguments of Khrennikov and Zeilinger^27^, it is possible that a purely mathematical approach to randomness and the formalization of its definition seems to be out of reach, as mathematical tools may be insufficient to formulate a theoretical framework for the concept of randomness. Perhaps it is rather physical processes that are the realm of reality in which there is true randomness beyond classical determinism in the area of quantum physics phenomena perceived as fundamentally nondeterministic.

### The problem of randomness in quantum mechanics

The Copenhagen interpretation of quantum mechanics was formulated by N. Bohr and W. Heisenberg in 1927 in Copenhagen, based on the idea of M. Born to interpret the wave function in a probabilistic manner (e.g., for a wave function in a positional representation, square of its modulus represents the probability density of finding a particle at a given point of the space^28^). Nowadays, this interpretation is often called the standard one, despite the intensive development of a competing probabilistic concept called Quantum Bayesianism proposed by Fuchs^29^, which contradicts some basic assumptions in the Copenhagen interpretation.

In the Copenhagen representation, the measurement of a quantum system randomly selects one of the many possible classical states initially realizing the superposition of quantum states (cf. Supplementary Information B). This choice is due to the so-called collapse of the wave function due to the interaction of an external observer (i.e., an external macroscopic measuring system characterized by a number of degrees of freedom corresponding to at least the Avogadro number, cf. Supplementary Information B) with the measured quantum system. The quantum measurement scheme has been the subject of the research by von Neumann^30^. As a result, von Neumann proposed an “ansatz”, which can be formulated as an axiom stating that when the measurement takes place, the wave function of the quantum system collapses in a truly random manner into one of the states of the measured observable basis (cf. Supplementary Information B). At any given moment *t* in time (at which the measurement takes place), this can be written as:

1 $$\begin{aligned} \begin{aligned}{}&\psi \left( {\mathbf {r}},t \right) = \sum _{i} {c}_i(t) \phi _{i} \left( {\mathbf {r}} \right) , \\&{\hat{A}} \phi _i = \lambda _i \phi _i, \\ \end{aligned} \end{aligned}$$

where ${\mathscr {H}}_{{\hat{A}}}$ is a Hilbert space spanned by eigenstates of the observable (Hermitian operator in the Hilbert space)${\hat{A}}$ forming the base $\left\{ \phi _i \right\}$, and $\lambda _i$ is the eigenvalue of the ${\hat{A}}$ operator (cf. Supplementary Information B). In this formulation, the measurement consists in selecting, in an unpredictable random manner, one state $\phi _i$ for which $c_i$ is non-zero. In such a case, the measured quantum system assumes a randomly selected state $\phi _i$ and the eigenvalue $\lambda _i$ is reflected in the macroscopic number of degrees of freedom of the measuring device, which allows us to observe it in the classic way as the result of a measurement of a certain physical observable corresponding to ${\hat{A}}$. The square of the modulus of $c_i$ coefficient defines only the probabilities of the occurrence of different $\phi _i$ eigenstates of the measured observable as a random result of the measurement. It is assumed that there are frequency probabilities in this approach, which, however, poses a significant problem in relation to the formulation of quantum mechanics. Such an approach assumes the existence of an infinite number of identical copies of the measured system, for which the occurrence of certain states in the results of measurements on subsequent copies, will be the distribution determined by the coefficients $c_i$. This, however, runs counter to the property of quantum measurement, which is destructive and unique (cf. Supplementary Information B).

In other words, quantum measurement is an irreversible process that destroys the original quantum state, and is therefore a unique process (which cannot be repeated). This process distinguishes the observer who performs this single, unique measurement. Additionally, Żurek’s fundamental non-cloning theorem^31^ ensures that there is no possibility to copy the unknown quantum system being measured, emphasizing the truly unique nature of quantum measurement.

The frequency probability mentioned above is an interpretation that defines the probability of an event occurring in a given process as a limit of the relative frequency of obtaining such an event in the implementation of an infinite number of such processes.

2 $$\begin{aligned} P \left( x \right) = \ lim_{N \rightarrow \infty } \frac{n}{N}, \end{aligned}$$

where *N* is the number of process repetitions, and *n* is the number of process reruns in which the event took place. In this context, there is the problem of the difficulty of repeating a quantum measurement infinitely many times. This problem was at the heart of the concept of a probability paradigm in quantum mechanics. Fuchs explains this by analogy to the weather forecast^29^. When forecasting the weather for the next day, we are dealing with a situation that has never happened in the past, therefore we cannot refer to the frequency probability paradigm (as this corresponds to phenomena that can be observed repeatedly). Instead, the Bayesian probability paradigm should be considered. The weather forecast must be made on the basis of the knowledge of similar but not identical situations. Therefore, it can be determined on the basis of the conditional probability. Fuchs argues that the same is true for the case of the quantum measurement as it is a unique process due to its destructive nature. This approach is called quantum Bayesianism or QBism^29^. The state of a quantum system can be regarded as objective (characterized by the measure of objective probability) or as subjective, measured by the observer’s expectation with respect to this system (the approach represented in QBism). These differences can have important ramifications for the concept of a quantum random number generator. Therefore, the problem of randomness can also be related not only to the technical imperfection of the implementation of a given solution based on quantum mechanics, but also to the interpretation of quantum mechanics itself, which is not unambiguous in this respect.

In the quantum aspect, the cause of the randomness is an unknown quantum state (an unknown coherent superposition of known states, cf. Supplementary Information B), and the random decoherence (measurement) provides an unpredictable random variable. However, a question arises here about the preparation of the unknown state and whether it is perhaps known to another observer who could possibly communicate with a local observer taking a measurement on what he believes is an unknown state. Therefore, it seems that the true definition of the randomness may be based on the quantum measurement process, but only of a true quantum information, and therefore fundamentally undefined—unknown to any classical observer. Whether such information exists is an unsettled question and raises problems of philosophical epistemology. One can, however, notice some interesting properties of the randomness of such information if it did exist. It turns out that the randomness contained in a single qubit of such unknown information may be equivalent to the randomness contained in any arbitrary number *n* of qubits and is related to the concept of quantum entanglement (cf. Supplementary Information B).

## Quantum Random Number Generator (QRNG)

Generating sequences of random numbers is of great practical importance as indicated above. Such sequences are crucial in IT security implementations, e.g. cryptographic techniques (both classical^32,33^ and quantum^34–36^), in numerical mathematical calculations and simulations (mainly in Monte Carlo calculations)^37,38^, in physical tests^18^, in games and lotteries etc. The available numeric routines only generate pseudorandom bit sequences. They are sufficient, for example, for computer games, but for cryptographic security techniques and for accurate mathematical simulations, they do not meet the randomness requirements (they can be tested using probability calculus and statistics^26,39^, however, it must be remembered that a selected fragment of a pseudorandom sequence may successfully pass randomness tests, yet still remaining deterministic in its nature). This situation results from the statistical nature of the tests themselves and means that the decisive factor is the negative (rejecting the sequence as definitely pseudorandom) rather than the positive result of such tests. An example of a simple pseudorandom number generator is the congruence algorithm (*Linear Congruential Generator*): (*a*, *b*, *m* are appropriately selected known constants): the initial state is the seed value, the output bit which is taken arbitrarily, the next bit is generated according to the recipe: $new \; state = a {\tilde{A}}\; - old \; state + b \; mod (m)$, $generated \; bit = new \; state \; mod (2)$. It is a pseudorandom algorithm: (1) it becomes periodic easily, (2) there are known methods of guessing *a*, *b*, *m* based on the sequence observation. Another example of a pseudorandom generator is an iterative call to a cryptographic hash function (such as MD5 or SHA1). All pseudorandom generators are not safe, i.e., the pseudorandom sequences they generate can be predicted with sufficiently large computational resources expenditures, as a result of which these sequences lose the attribute of randomness. Often pseudorandom sequences are generated with classical physical pseudorandom number generators related to the physical attributes of the computer itself, such as, e.g., hard to predict intervals of input-output activity in the computer, fluctuations in processor temperature, or the frequency of the keypad signal. The various hardware electronic noise generators considered to be truly nondeterministic are in fact pseudorandom generators. For example, we can use the analogy of randomness in the case of bubbles of water vapor on the surface of boiling water. Considering the microscopic nature of the initiation of the production of closed surface elements (vapor bubbles) inside the liquid, when the water vapor pressure exceeds the hydrostatic pressure, it could be assumed that the volumetric boiling which translates into an irregular, dynamic pattern of the surface of the boiling water is random. However, it is easy to notice that, for example, by pouring a small amount of sand into the water, you can determine the points of bubble formation, and the appearance of the boiling water surface can be strongly changed through the sides of a strong fan – in this way it is easy to introduce the so-called *bias*, with which it is possible to substantially modify a seemingly random behavior. This may also be similar to a simple bias for the throw of an asymmetrically loaded dice or a coin with an asymmetrically profiled edge. In such cases, the disruption of randomness in large sequences may be very important (which is easily illustrated by dishonest tricks, e.g., in games with marked cards, loaded dice, or magnetically distorted roulette).

### Quantum rules for generating true random sequences

The generating of truly random sequences of bits by biased generators is an important challenge in computer science, cryptography, and statistical applications. The dominant view is that no classical realizations are able to generate truly random sequences of bits, because of the determinism of the laws of classical physics. So what remains is quantum physics, and it is referenced by the quantum random number generators QRNGs. Why is quantum physics unique in this regard? The answer is related to the von Neumann projection axiom^28^ adopted in quantum mechanics (cf. Supplementary Information B) concerning the absolutely random unpredictable result of quantum measurement. According to the quantum picture of the world, the state of a given system (let’s say a particle) is determined by a complex wave function, varying in time and space, which is an element of a Hilbert space – a linear complete space (i.e., Banach space) according to the metric induced by the scalar product. The square of the module of the wave function determines the probability of finding a particle at ${\mathbf {r}}$ at time *t* (normalization to 1 of the modulus squared of the wave function means that there is a single particle in the entire 3D space at any time *t*). If the observer does not measure (observe), then the quantum state evolves unitary and deterministically in the Hilbert space according to the Schrödinger equation,

3 $$\begin{aligned} i \hbar \frac{\partial \Psi ({\mathbf {r}}, t)}{\partial t} = {\hat{H}}\Psi ({\mathbf {r}}, t), \end{aligned}$$

where $\hbar = 1.05 \times 10^{-34}$ Js is a Planck constant, and ${\hat{H}}$ is the operator of energy called the Hamiltonian. The equation (3) replaces the Newton equation in quantum mechanics. It is also a differential equation, but of the 1st order with respect to time and does not give a phase trajectory, unlike the Newton equation, which as a 2nd order differential equation with respect to time clearly defined the phase trajectory – position and momentum (or speed = momentum/mass) under given initial conditions for position and momentum. It was the so-called classical determinism – a future unambiguously determined by the past (initial conditions, and a given equation). The Schrödinger equation (3) also gives determinism, like any differential equation satisfying the existence and uniqueness theorem (which can be written as: for ordinary differential equations satisfying the so-called Lipschitz condition—it is satisfied e.g., for continuous and smooth functions giving an equation—exists one and only one solution to a differential equation passing through a given initial condition; these unique solutions are also generalized to partial differential equations, which include the Schrödinger equation, due to the differential form of the Hamiltonian), but it is quantum determinism, i.e., the wave function traverses in the Hilbert space an unequivocal trajectory for a given initial quantum state $\Psi ({\mathbf {r}}, t = 0)$ (solution to the equation (3)),

4 $$\begin{aligned} \Psi ({\mathbf {r}}, t) = {\hat{U}} \Psi ({\mathbf {r}}, t = 0) = e^{i {\hat{H}} t / \hbar } \Psi ({\mathbf {r}}, t = 0), \end{aligned}$$

where the evolution operator ${\hat{U}} = e^{i {\hat{H}} t / \hbar }$ is unitary, ${\hat{U}}^+ = {\hat{U}}^{-1}$ (this is what it is for the Hermitian Hamiltonian, ${\hat{H}}^+ = {\hat{H}}$, here plus means a Hermitian conjugation (in quantum mechanics, the Hilbert space is often chosen as the space of functions integrable with their modulus-square, the so-called $L^2$ space with the scalar product of the function defined as follows, $(\Psi , \Phi ) = \int \Psi ({\mathbf {r}}) \Phi ({\mathbf {r}})^* d^3r$) defined on the scalar product according to the formula $({\hat{A}} \Psi , \Phi ) = (\Psi , {\hat{A}}^+ \Phi )$). The unitarity of the evolution operator guarantees the preservation of the scalar product, the base and the dimension of the Hilbert space, and generally the preservation of the ’quantum information’ contained in the wave function during the evolution. However, this information is not available to the observer (his awareness) who understands only the measurement result in the form of a single real number (he has a classic awareness oriented towards classical measurements). It should be noted here that the classical measurement in classical physics was non-destructive, repeatable and did not distinguish the observer (e.g., measuring the length of a pencil does not destroy the pencil and can be repeated by various observers—as a result, a random variable is obtained depending on the accuracy of the measuring cup and the care taken in making the measurement—this randomness is pseudorandom, classic and related to the measuring device, and it is easy to introduce here a bias).

The quantum measurement is different—it is destructive (during the measurement the measured state of the system disappears irretrievably), unique (because it is destructive) and it distinguishes only one observer. The measurement result is absolutely random. This is where the quantum randomness according to the von Neumann axiom is located. You can measure observables in quantum mechanics, i.e., quantities represented in Hilbert space by Hermitian operators, those that do not change under the influence of the Hermitian conjugation, ${\hat{A}}^+ = {\hat{A}}$. The eigenfunctions of Hermitian operators create ON (orthonormal) bases in a Hilbert space, and the corresponding eigenvalues are real (for the operator ${\hat{A}}$ in a Hilbert space, the solution to the equation, ${\hat{A}} \psi _j = \lambda _j \psi _j$, defines the eigenfunctions $\psi _j$ of this operator and eigenvalues $\lambda _j$; for Hermitian operators, ${\hat{A}}^+ = {\hat{A}}$, i.e., measurable observables—e.g., the momentum operator $\hat{{\mathbf {p}}} = - i \hbar \nabla$, operator of the position $\hat{{\mathbf {r}}} = {\mathbf {r}}$, energy operator ${\hat{H}} = \frac{- \hbar ^2 \nabla ^2}{2m} + V ({\mathbf {r}})$, $V ({\mathbf {r}})$ is the potential energy—eigenfunctions create ON bases in Hilbert space and eigenvalues are real—such as those needed for the results of measuremets understandable for classic consciousness). If an observer measures the size-observable on the state of the system at some selected point in time *t*, then according to the von Neumann axiom follows:

1. The state collapses to one random observable eigenfunction;
2. The measurement result is the real eigenvalue of this randomly selected eigenfunction;
3. The system continues its evolution by starting with a new random start function.

**Figure 1:**
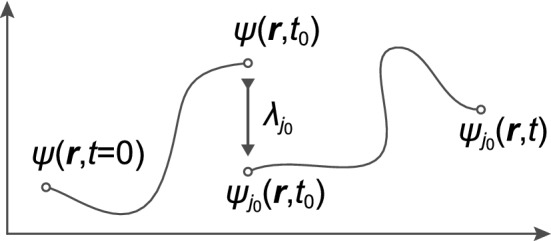
Von Neumann projection scheme: the quantum state at the moment of measuring the size of the observable ${\hat{A}}$ collapses to a randomly selected eigenfunction of this observable, $\psi _{j_0}$ and the measurement result is a random value on of this corresponding eigenvalue $\lambda _ {j_0}$ (the real number). The result is completely random and the measurement destroyed the state of $\Psi ({\mathbf {r}}, t)$, which ’remembered’ its initial condition $\Psi ({\mathbf {r}}, t = 0)$ and the system has any further memory on this state. The new evolution no longer remembers this state and begins with a completely randomly selected eigenfuncion of the observable $\psi _{j_0} ({\mathbf {r}}, t_0)$.

As a result of von Neumann projection, the system ’forgot’ about its previous unitary evolution (deterministic in Hilbert space) from its previous initial state $\Psi ({\mathbf {r}}, t = 0)$ and accidentally jumped at the time of the measuring *t* for further evolution but already from the state corresponding to the eigenfunction of the measured value $\psi _{j_0} ({\mathbf {r}})$, completely independent of $\Psi ({\mathbf {r}},t)$. It is schematically shown in Fig. 1.

Von Neumann’s projection (collapse) is completely accidental – it is also irreversible, because to its final state the system could be projected from various states and it is not known (due to ambiguity) to which it should be returned, and the true initial state of the system disappeared during the measurement. The von Neumann scheme determines only the probability of a random selection of the eigenstate of the observable with the number $j_0$. Due to the fact that the eigenvalues of the observable created the base ON in the Hilbert space, the state could be presented in this base,

5 $$\begin{aligned} \Psi ({\mathbf {r}}) = \sum _i c_i \psi _i ({\mathbf {r}}), \; \; \sum _i |c_i |^2 = 1, \end{aligned}$$

in the equation (5) the coefficients $c_i$ are complex (the Hilbert space is defined over the body of complex numbers in the quantum mechanics) and the sum of their squared modulus is equal to 1 due to the normalization of the system wave function and of eigenfunctions of the observables. The numbers $| c_i |^2$, real, non-negative, not greater than 1 (as can be seen from (5)) are probabilities – this is the probability of von Neumann’s projection to select the state *i*. All of these states are probable as long as $| c_i |^2> 0$, but only one of them, $i = j_0$, is realized. Which $j_0$ is, it is not known in advance—only after the measurement it is known how the von Neumann projection took place. It is a random process. It is not covered by the unitary quantum evolution according to the Schrödinger equation. It is not known why the von Neumann projection takes place and why it is random and introduces irreversibility during the measurement. The von Neumann projection, however, is correct with all known (thousands and more) different quantum measurements and is therefore taken as an axiom.

To approximate the von Neumann projection scheme, a simplified classical illustration can be used (however, it must be remembered that this is an incomplete analogy and the von Neumann scheme cannot be explained classically). The quantum state can be visualized as the unknown state (unknown position) of the coin in the hand before it is thrown. When the coin is thrown, this condition disappears and one of the two ’eigenstates’ of measurement is carried out randomly—heads or tails with a probability of 1/2 for each of the results. After the measurement, the coin is in one (random) own state and the measurement result is the corresponding ’eigenvalue’—heads or tails. This simple example illustrates the content of the scheme but is classic—-the coin toss is deterministic according to classical mechanics. The von Neumann projection is not deterministic. Therefore, the von Neumann scheme is applicable to the generation of truly random numbers and sequences that are unbiased in a fundamental manner guaranteed by the laws of quantum mechanics. The implementation of a quantum random number generator based on the von Neumann projection requires: (1) the ability to implement this projection in practice on a specific system, (2) the ability to prepare the system for projection in the same known state. Neither (1) nor (2) is easy to provide, and therefore the structure of the QRNG is not simple and develops with advances in experimental quantum mechanics. Referring to the above-mentioned condition (2), one should note an important limitation here. According to the fundamental theorem of quantum computing^31,40^, (No-cloning), you cannot make copies of an unknown quantum state, only a known state. This is due to the linearity of quantum mechanics, while the state copy is nonlinear (quadratic). The nonlinearity of the copy conforms only to the numbers 0 and 1 (because 0 or 1 squared is still 0 or 1). These values of 0 and 1 correspond to two basis vectors of qubit (and therefore known qubit states – a two-dimensional state in the simplest Hilbert space with the basis $| 1>$ and $| 2>$, the qubit $| \Psi> = c_1 | 1> + c_2 | 2>$). The known state here is $| 1>$ or $| 2>$, for which $c_1 = 1, \; c_2 =$ 0 or $c_1 = 0, \; c_2 =$ 1, respectively. These states are copyable. Unknown states, on the other hand, are states with $c_1 = x, \; c_2 = \sqrt{1-x^2}$, ($| c_1 |^2 + | c_2 |^2 =$ 1) and those with unknown $x \in R, \; x \in (0,1)$, cannot be copied. The QRNG scheme would therefore include the preparation of a series of known states and carrying out a von Neumann projection on them in a different base than they were prepared. If you perform a projection in the basis of another qubit, you can obtain an absolutely random sequence of eigenvalues of the measured observables – two eigenvalues for a qubit, which can be identified with bits 0 and 1.

### Types of QRNGs

The premise of the absolute randomness of hardware quantum random number generators is the belief that the von Neumann projection is perfectly random. Thus, the measurement on the superposition state of at least two states (qubit) leads to the generation of a random sequence. Two stages must be distinguished here:

1. Preparation of the input state (it can be the same known quantum state or also a randomly selected state of the source—-in the latter case the possible randomness of the source and its quality is also important for the randomness of the second stage—the measurement).
2. State series measurement—this process generates a quantum randomness and ideally guarantees the absolute randomness of the final sequence.

The absolute randomness of the final sequence can be compromised by the faulty source. If, for example, the source will provide its own state of the measured quantity with some frequency, the randomness of the result will be strongly disturbed. Therefore, step (1) is as important as step (2). Moreover, the result of step (2) is always to some extent mixed with the classical noise resulting from the macroscopic practical implementation of the von Neumann projection. It should be emphasized here that von Neumann projection is always performed with a macroscopic device and only in an idealized situation the arrangement of a measurement experiment does not introduce random classical disturbances. The generated sequence of bits is extremely susceptible to various forms of the bias. Reducing bias is relatively simple, whereas identifying the classical implicit component (correlation) involved in the generated sequence is much more difficult and not always effective by software methods. Rather, we should rely on the physical recognition of the whole phenomenon and physical identification and minimization of the classical components of randomness. Various signal whitening algorithms are available for bias reducing and de-correlation. They are the most common development of the von Neumann algorithm. According to this algorithm, two successive bits of the sequence are compared, if they are the same, both are rejected, if they are 0,1, then 0 is assumed, if they are 1,0, this is assumed to be 1. The resulting sequence is balanced, but at least twice as short and random as there is no correlation in the output sequence. More advanced randomization extractors are e.g., Trevisan extractor^41^ or Toeplitz extractor using Fast Fourier transform^42^. In general, random sequence bleachers work by themselves as pseudorandom generators. A good example is the Blum, Blum, Shub (BBS) algorithm^43^. It returns the sequence from the output seed $x_0$, according to the recipe,

6 $$\begin{aligned} x_{n + 1} = x_n^2 mod (M), \end{aligned}$$

where $M = p \times q$, *p*, *q* are high prime numbers. The bit-wise result of the procedure is $x_{n + 1}$ parity or, for example, the last significant bit $x_{n + 1}$. The seed $x_0$ must be relatively prime to *q* and *p* and cannot be 0 or 1. An interesting feature of the BBS generator is the analytical form of the result,

7 $$\begin{aligned} x_i = \left( x_0^{2^i mod \lambda (M)} \right) mod (M), \end{aligned}$$

where $\lambda (M)$ is the Carmichael function. This function, defined by a positive integer *n*, denoted as $\lambda (n)$, is defined as the smallest positive integer *m* such that $a^m = 1 mod (n)$ for each integer of *a* relatively prime with respect to *n*. So it’s easy to guess the whole random sequence knowing the seed and numbers *p*, *q*.

The use of various anti-bias and anti-correlation algorithms (balancing and decay whitening) is a software-based raw sequence processing and completely deterministic (though usually difficult in terms of hash functions). Despite the effective removal of bias, excessively complicated procedures of randomness extractors can themselves disturb/obscure the quantum randomness contained in the raw sequence, adding their own pseudorandom component to the mixture with classical noise contribution. Therefore, it is important to search for hardware solutions of a quantum random number generator with a relatively small classical admixture. QRNGs using the von Neumann qubit measurement, e.g., of a photon registering, are limited in the relaxation rate of the measuring device – single photon detectors (e.g., avalanche diodes or photomultipliers) have an inertia of the order of 100 ns, which limits the random sequence generation rate to Mb/s. This is too low a generation rate for cryptographic applications where the required speed should be up to Gb/s or even 100 Gb/s. According to the review of QRNGs^44^, such gigabyte speed can be demonstrated in generators strongly supported by software, which is a compromise for performance.

QRNGs that test the quantum randomness of the generated sequence are also proposed. Quantum randomness authorization is used here by verifying violation of the Bell’s inequality and discarding fragments not meeting this criterion^28^. Such generators obtain a high level of confidence even with incompletely characterized and random sources. However, they do slow down the routine^44^.

### Application of Fermi golden rule to QRNG constructs

The main and innovative goal of this paper is the analysis of a new and original concept of QRNG not based on conventional von Neumann projection. The work to date on the quantum generation of randomness has been limited mainly to the concept of the von Neumann projection and the related unpredictability of its result. In a heuristic way, in relation to QRNGs, the randomness of quantum tunneling through a semiconductor barrier junction was also discussed, based on the fact that only the probability of the tunneling is also random in a quantum sense.

We notice, however, that in quantum mechanics not only the von Neumann projection is the source of randomness (or possibly tunneling). In our opinion, the essence of quantum randomness is the interface between quantum and classical information. Ihe classical reading of quantum information is the source of randomness. Without measurement, the system remains in a coherent superposition. As a result of the measurement the coherent superposition is removed in a random manner. The cause of the random result seems to lie in the percolative trajectory of loading quantum information into a classical measuring device at the level of its microscopic structure (cf. Supplementary Information B), and not in the measured system (qubit). The cause of randomness is the decoherence.

Fermi golden rule^28^ (cf. Supplementary Information B) describes the probabilities of a quantum transition per time unit under the influence of a time-dependent perturbation (switched on at some instant and turned off after some time), but with the continuous spectrum of the final states for a quantum system. Here is also involved the decoherence being the source of the randomness. The probability of a quantum transition in a discrete spectrum of a quantum system induced by the time-dependent perturbation is proportional to $T^2$ (*T* is the time duration of the perturbation action)^28^, only after introducing the continuous spectrum of the measured system it attains the features of classical probability proportional to *T*, so that the transition probability per time unit is constant. A purely quantum transition in the discrete spectrum is clearly non-classical—proportional to $T^2$, i.e., ’accelerates’ with the passage of time, which we do not observe in the classical world. This ’acceleration’ can be understood by solving the exact quantum problem of so-called Rabi oscillations^28^—i.e., of cyclic transitions between two qubit stationary states upon the time dependent periodic perturbation (cf. Supplementary Information B). The probability of a transition between these states is determined by the function $\sim sin^2(\alpha T) \simeq T^2$, where the last approximation is correct for small *T*, that is, in accordance with the quadratic time dependence of the transition probability in the time-dependent perturbation calculus mentioned above. The transition ’accelerates’ quadratically but next it slows down squarely and ’accelerates’ in the opposite direction—Rabi’s quantum oscillations arise. However, when the final state of the system (then it is not a qubit) belongs to the continuous spectrum, Rabi oscillations and the quadratic acceleration of the transition disappear with time and the conventional classical transition takes place with a constant probability per time unit. It is important because this transition which looks like classical is in fact quantumly random. Thus here is hidden a source of truly random signals. The quantum transitions according to the Fermi golden rule is unconditionally random, as in the von Neumann projection, because, similarly to the von Neumann measurement scheme, decoherence works here—either as a collision of a small quantum system (qubit) with a giant (with of order $10^{23}$ degrees of freedom) classical system, in which the result is being imprinted, or as an introduction, by the giant classical system, of a continuous (like in the classical case) energy spectrum of final states for quantum transitions. Thus, the Fermi golden rule is as good for implementing the QRNG as is the von Neumann projection. The processes according to the Fermi golden rule are e.g., absorption, emission (induced or spontaneous) of light, registration of radio waves, thermal emission of electrons and numerous electronic effects at the microscopic level—everywhere where in the Boltzmann-type kinetic formalism all collisions are governed by the Fermi golden rule. Plasmon coupling effects, for example, of a metallic nanoparticle illuminated with light with a substrate of a semiconductor solar cell, are also quantum random events. Fermi golden rule opens up a huge reservoir of various possible QRNGs arrangements not exploited as of yet. Going in this direction, one would only need to determine the source of entropy—the initial state undergoing the quantum transition according to the Fermi golden rule. A standard photovoltaic cell, a glowing photo-emission diode or even a light bulb, are examples of an entropy source—the resulting noise of the relative average signal from these devices will have a high entropy quantum component. However, each time it is necessary to analyze additional classical and thermal noises, which would mix with the true quantum randomness. The question here arises to what extent a thermal noise is separable from a quantum noise—in quantum statistical thermodynamics it is not separable^45^. It should be remembered that classical thermodynamics (Boltzmann decomposition) is just the theoretical boundary of something more general—quantum Gibbs distribution (canonical or grand canonical ensemble)—in which one cannot separate quantum noise from ’thermal noise’. The thermal noise in QRNG implementations is not a disturbing and not a pure classical component—although it causes a strong bias, but the fluctuations around the mean value of the signal always remain quantum. This is similar to the quantum properties of light (known from quantum optics^46^)—each light is in fact quantum in the sense that either the number of photons or the phase of the e-m field and a related to photons e-m field itself cannot be simultaneously determined according to the uncertainty principle. Even if we deal with absence of photons (full darkness), i.e., the number of photons equals 0, the e-m field cannot be zero and must fluctuate randomly without any determined value^46^. The same happens for any fixed number of photons, which must be associated with a randomly fluctuating e-m field. And conversely, if the e-m field is steady well-defined, then the number of photons must randomly fluctuate. Such types of randomness are absolutely unpredictable—they are quantum.

### Commercial QRNGs summary

The Table 1 summarizes the essential features of the commercially available quantum random number generators presented in the Supplementary Information C. It is worth emphasizing that regardless of the quality of the quantum nature of a given generation process, the testability of, among others, NIST battery testing is the primary advantage presented by the manufacturers. Some important aspects have been omitted from the table, e.g., the possibility of being miniaturized to the chip size. Doubts about the nature of the source of entropy in given solutions were also presented. None of the currently available commercial generators is based on quantum entanglement, which is most likely caused by low generation speed parameters, implementation difficulties or costs of such solutions – but on the other hand, no doubt the fundamentally quantum nature would outweigh these disadvantages.

**Table 1:** Features of commercial quantum random number generators.

QRNG	Generation speed Mb/s	Link	Source entropy	Self-test.	Compatibility	Bit access	Doubts
Quantis	4, 16	USB, PCIe	Quantum optics.	Yes	NIST, METAS, CTL, BSI’s AIS31	No	Quantum simulation
ComScire	4, 32, 128	USB	Electr.	Yes	All	No	Quantum simulation
Toshiba	8000	USB, SATA	Quantum optics.	No	TestU01, NIST	No	Quality of single-photon detectors
PQRNG150	150	USB	Quantum optics.	No	Confirmed for selected	No	Quality of single-photon detectors
Entropy engine	350	PCIe	Quantum optics.	No	NIST, Alphabit, Dieharder, FIPS140, TestU01	No	Quality of single-photon detectors
qStream	1000	Ethernet	Quantum optics.	Yes	NIST and chosen	Yes	Quantum simulation
QNG2	1000	Chip	Tunnel. quantum.	No	NIST, Dieharder	No	Quantum simulation
MQRNG	40000	USB, PCIe, PCM	Radioactic decay.	No	NIST, AIS.32, Diehard	No	Measurement method
quRNG	50	USB	Quantum optics.	Yes	NIST, Dieharder	No	Quality of single-photon detectors
MPD QNRG	16, 32, 64, 128	USB	Quantum optics.	No	NIST, Dieharder, TestU01	Yes	Quality of single-photon detectors
QRNG100E	200, 600	USB, Ethernet	Quantum optics.	no	GM/T 0005-2012 and NIST	Yes	Quantumness of the process
Quside FMC 400	400	USB, PCIe, Ethernet	Quantum optics.	Yes	Quside randomness metrology	Yes	Quantumness of the process

## Randomness tests for generated bit sequences by statistical analysis methods

A very important aspect of the generation of random bit sequences is testing whether the obtained sequence is actually random or not. Despite the problems with the formal definition of randomness (related to an uncountable number of infinite zero-one sequences), it is possible to define some probabilistic or statistical properties of a perfectly random sequence (e.g., the simplest such property is an equal average number of zeros and one in the whole sequence and in any of its trains/subsequences). With regard to these properties, it is possible to characterize any generated sequences and check whether in the language of these statistical correlations they are closer or further to the perfectly random sequence.

There is, however, a key problem here, namely there are an infinite number of possible statistical tests (e.g., a test of the occurrence of a certain pattern, and such patterns in the case of infinitely long sequences are also infinitely many). Therefore, there is no complete set of tests, only some tests that seem to be sufficient for the given applications (others for the requirements of only the uniqueness of the generated sequence and others for cryptographic security). For many years, a certain balance has been sought between the range of tests that can be performed effectively (in terms of available computing resources) and the level of randomness guarantee that results from them.

In the Supplementary Information D, the most popularized so-called test batteries or test suits, which are the accepted standard for randomness verification, are shortly characterised. A detaild description of the NIST Statistical Test Suite can also be found in the Supplementary Information E.

## Examples of RNG tests and comparison

### Commercial QRNG PQ4000KSI

**Figure 2:**
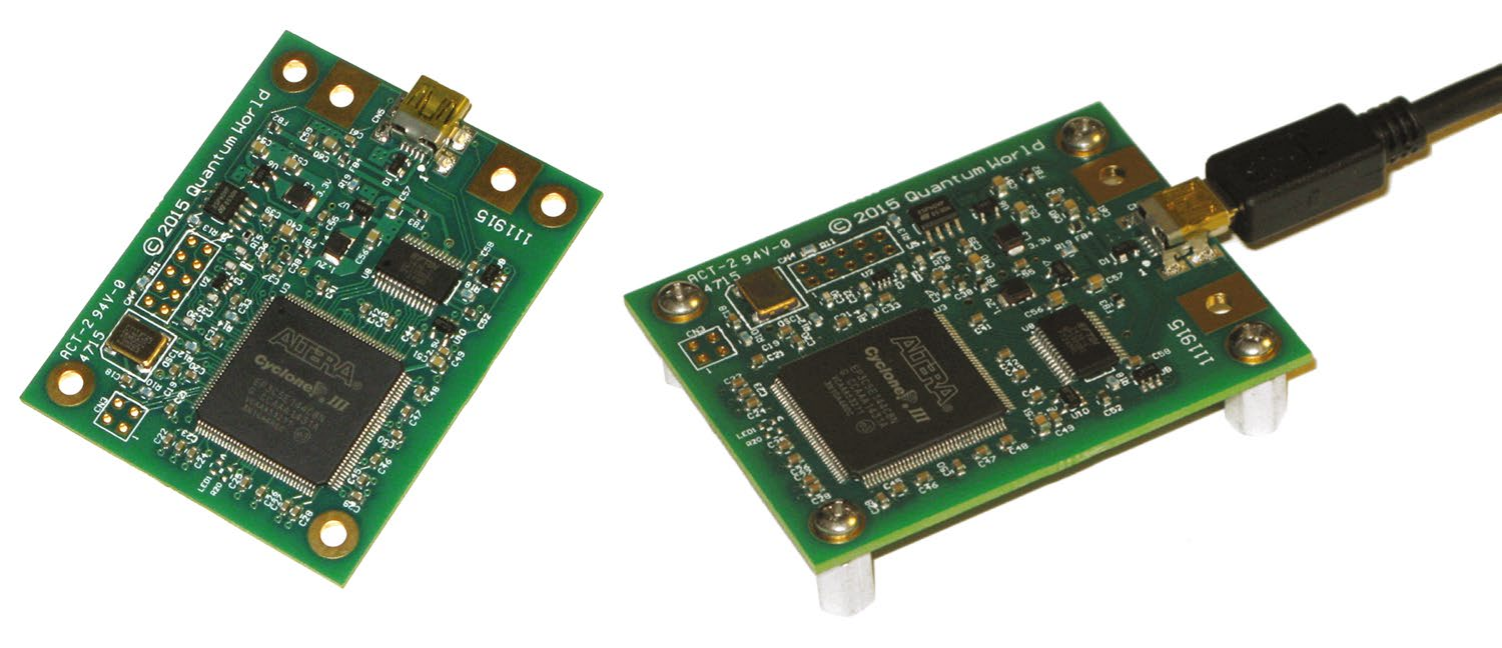
PQ4000KSI quantum random number generator after removing the cover. Generator performance 4Mb/s of random binary code.

QRNG PQ4000KSI (Fig. 2) is manufactured by the American company ComScire US.

According to the manufacturer the offered product is a quantum random number generator, although the quantum process, being the source of entropy, is not identified. As referred to a shot noise in a transistor, it is therefore a quantum random number generator operating according to the Fermi golden rule and the software technique of *bias* removal and whitening the signal used in it is so effective that the manufacturer declares a high level of randomness of the output produced at 4 Mb/s. The device is equipped with a USB connection for power supply (90 mA). The manufacturer’s suggested quantum entropy is 0.999. The manufacturer argues that the thermal *shot noise* and subliminal tunneling on the transistor translates into voltage fluctuations on the capacitor, from where the signal is collected to the binary converter. Although the literature discusses the classical components of the *shot noise*, the manufacturer claims that the source of the noise, in his product, is the tunnel outflow of carriers on a semiconductor junction. The tunnel effect, apart from the quantum name, does not identify the von Neumann projection, but rather it can be related to the probability of crossing the barrier, which can be seen as Fermi golden rule quantum randomness. An additional argument is the nanoscopic junction scale (60 nm) provided by the manufacturer.

### QRNG JUR01

**Figure 3:**
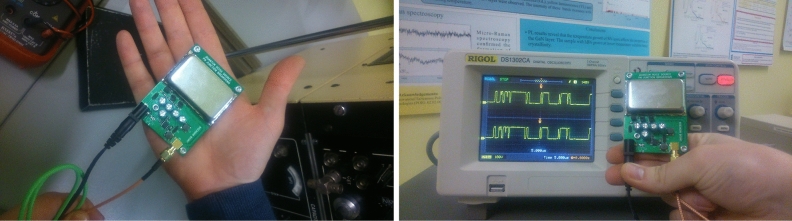
JUR01 quantum random number generator after removing the cover. Generator performance 4Mb/s of random binary code (on the right panel visible signal 0,1 on the oscilloscope screen).

The second tested random number generator is QRNG JUR01 produced at the Department of Quantum Technologies of WPPT PWr (as part of the NCBiR project POIR.01.01.01-00-0173/15)—Fig. 3. The quantum source of the entropy is also not explicitly identified in this system and similarly refers to the subliminal conductivity across the barrier of a transistor junction, and thus realized with the probability of quantum tunneling—quantum transition acc. to Fermi golden rule. It should be noted here that in quantum solutions the entropy of a bit is assumed to be a’priori equal to 1, taking the position of the absolute unpredictability of the value of this bit. At the same time, for a pseudorandom bit, entropy is assumed to be 0, because the value of this bit can be predicted, although often the computational resources expenditure can be very high, which in practice makes it difficult for classical computers to guess such bit (however, it is possible and therefore entropy is 0). The reduction of the entropy of the quantum bit occurs as a result of the admixture of classical deterministic chaos. Using software techniques for the *bias* removal and the signal whitening, in turn, increases the entropy. However, you can never be sure of the role of individual factors, and you should approach their evaluation with caution, especially when they are given by the manufacturer. In this context, therefore, the possibility of sampling the randomness of the sequence generated with statistical methods and adopting contractual randomness quality criteria becomes important.

### Pseudo-RNG under Mathematica Wolfram

The third generator tested is a pseudorandom generator within the Mathematica Wolfram system. In order to generate a 100 MB of binary sequence, code that generates 0 and 1 was applied to a text file (ASCII encoding) in the *RandomChoice* function, which with equal probability selects the given values as a parameter inside curly braces^49^. The *RandomChoice* function returns various sequences of pseudorandom selections depending on the *seed* established by the *SeedRandom* function (by default, the *seed* value depends on the time and certain parameters of the current Wolfram session) and on the adopted pseudorandom generator method ($extended\; cellular \;automaton\; generator$ by default). Using the code, a text file with a size of 800 Mb was generated (ASCII encoding—each byte of the sequence represented by an 8-bit text code)—the data from the file was read as part of the NIST STS tests as ASCII encoded—hence in the next paragraph a binary sample of 100 MB length will be mentioned. The code used is shown below,

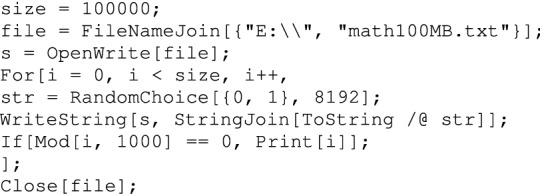



### Statistical testing of selected above generators

As part of the tests performed with the NIST STS randomness test library, 3 groups of data were compared. The first group is a collection of binary random sequences generated using a commercial Comscire quantum generator. The second group consists of random sequences generated by the current version of the JUR01 quantum generator. The third group are random sequences generated algorithmically within the Mathematica system.

Which tests should be selected for randomness analysis is a difficult question. It depends on the analyzed generator (data from a given generator), its usage and the determination of random errors that are not acceptable. Without such detailed information, all the tests in the NIST STS kit should be used in the randomness analysis. To apply the entire test suite, the *n* parameter (representing the length of a single sequence in bits) should be greater than 100,000. NIST Documentation STS^50^ recommends testing at least *k* = $\alpha ^{- 1}$ = 100 sequences (assuming $\alpha = 0.01$). This is also a suitable value for the *p* value distribution test (test at least 55 sequences). As NIST STS uses some approximation methods to process the value of *p*, the more sequences you test, the more accurate the results you get. STS NIST authors suggest testing 1000 or more sequences^50^ .

A set of NIST STS randomness tests (15 tests already described) was separately run for each group using the publicly available C test implementation offered by the NIST Institute^50^.

Most empirical randomness tests, including NIST STS tests, are based on statistical hypothesis testing. Each of the tests is constructed in such a way as to verify the null hypothesis that the test sequence is random from a particular point of view of that test, which may be defined by some statistics of bits or blocks of bits. The test statistics is a function of the test data and is able to compress the measured randomness into a single value—the observed statistics. In order to evaluate the test, the distribution of statistics for the null hypothesis (about the randomness of the tested sequence) must be known. Most NIST STS tests take the $\chi ^2$ distribution or the normal distribution as the reference distribution. The observed statistics is transformed to the value of *p* using the adopted reference distribution due to the fact that the value of *p* can be more easily interpreted. The value of *p* corresponds to the probability that a true random generator will produce a sequence less random than the sequence being parsed.

#### Statistical test input

Three 100 MB binaries were prepared for testing. The sample from the Comscire quantum generator was generated using dedicated software. The sample generated with the JUR01 quantum generator was provided by the constructor of the device. The sample from the pseudorandom generator was generated algorithmically in Mathematica. Additionally, three 10 MB samples were tested.

Each of the three 100 MB files was split into *k* = 1000 sequences (each sequence consisted of *n* = 800,000 bits) and then subjected to the testing procedure (the entire set of 15 NIST STS tests). The 10 MB samples were split into *k* = 100 sequences.

Some of the NIST STS tests are run in several versions, i.e., selected tests perform sub-tests and then test more properites, of analyzed sequence, of the similar type. For example, the incremental sum tests a given sequence forwards and backwards – the test involves two sub-tests. Table 2 summarizes the requirements for the values of test parameters included in NIST STS. The table also shows the number of sub-tests performed within a specific test. For the non-overlapping pattern test, the number of sub-tests performed depends on the *m* parameter – the number 148 corresponds to the default value of the *m* = 9 parameter.

**Table 2:** Summarizing of parameters required for test (^51^).

Name of test	*n*	*m* or *m*	Number of sub-tests
Frequency test	$n \ge 100$	–	1
Frequency test in block	$n \ge 100$	$20 \le M \le n/100$	1
Test of courses	$n \ge 100$	–	1
Test of courses in block	$n \ge 128$		1
Matrics test	$n > 38912$	–	1
Spectral test	$n \ge 1000$	–	1
Test of nonoverlapping patterns	$n \ge 8m - 8$	$2 \le m \le 21$	148*
Test of overlapping patterns	$n \ge 10^6$		1
Test of Maurera	$n > 387 840$		1
Test of linear complexity	$n \ge 10^6$	$500 \le M \le 5000$	1
Series test		$2< m < [\log _2 n] - 2$	2
Test of entropy		$m < [\log _2 n] - 5$	1
Test of increasing sums	$n \ge 100$		2
Test of excursions	$n \ge 10^6$		8
Variational test of excursions	$n \ge 10^6$		18

Basing on Table 2 the following parameters were selected (Table 3).

**Table 3:** Selected parameter values.

Test name	parameter	parameter value
Frequency tes in block	*M*	128
Test of nonoverlapping patterns	*m*	9
Test of entropy	*m*	10
Series test	*m*	16
Test of linear complexity	*M*	500

The test results (as a set of *p* values) can be interpreted in many ways. NIST has adopted the following two interpretations:

- testing the proportion of positively passing a given statistical test—the number of positively passing a given test should be within a specific range,
- testing the uniformity of the distribution of *p* values—*p* values calculated for random sequences should have an even distribution in the interval [0, 1).

Both the Comscire commercial quantum generator and the Mathematica pseudorandom generator passed the randomness tests (all 15 tests of uniformity of distribution and 15 tests of proportion for the samples from each generator passed). Both generators can then be considered as generating random sequences in the light of NIST’s statistical tests. It’s worth noting that the results for both generators are similar – the proportion test results for each test are around 99%. It is difficult to notice significant differences between the two generators (quantum and pseudorandom) in the analysis of the NIST STS results, which may indicate the fact that during the statistical testing of random sequences it is not possible to detect true randomness.

In the case of the current version of the JUR01 quantum generator, more than 7 tests for the first sample have failed (another test was carried out with the second sample—also failed) which, according to both the original NIST interpretation and the interpretation proposed by^51^, proves that the generator is non-random and biased. Probably the non-randomness character of the analyzed sample captured by NIST STS is caused by a design error in the current version of the generator under construction (which has not implemented any whitening procedure).

Tests for 10 MB samples in the case of the QRNG Comscire and PRNG Mathematica generators gave results indicating non-randomness, which were considered as statistical errors related to too few tested sequences—the results were not presented, as the results were focused on the larger 100 MB samples. The value of *p* as the result of a single randomness test that focuses on a given statistical property has a relatively clear interpretation, but in the case of a set of tests, interpretation of such results presents some problems. These tests (and their results) are often interrelated and interdependent. For example, if the frequencies of the occurances of ones and zeros are disturbed (unequal) for a given sequence, it is highly probable that the frequencies in 2-bit blocks will also be disturbed. For a clear interpretation of the results (in the form of a set of *p* values—a separate *p* value for each test), the dependencies/relationships between the test results should be analyzed. This was partially done in the work^52^, but due to the lack of information about the quality of the data (generator quality) used in the analysis and the omission of some tests, the authors^51^ proposed to interpret the results (generator randomness assessment) based on the number of proportion tests that ended with a negative result. For this purpose, reference probabilities of completing the tests of proportion and uniformity of distribution with a negative result were determined, depending on the number of tested sequences *k* and the significance level $\alpha$. According to the calculations, a sample of 1000 sequences can be considered non-random if 7 or more proportion tests fail. The NIST STS^50^ authors recommend that a sample should be considered non-random in the event of a negative result for one test – requiring the sample to pass all tests to be considered random. In^51^ it was noticed that for larger samples (greater number of tested sequences) the probability of failing one of the tests is higher and hence they propose an interpretation based on the analysis of the number of failed tests. In the event that the generator fails the randomness test (the sample is considered non-random), both^51^ and^50^ suggest that the test should be performed again for the next sample to determine whether the test result is a statistical anomaly or clear evidence that the generator is non-random.

**Proportion of sequences passing test** The probability that a random sequence will pass a given test is equal to the completion of significance level $1 - \alpha$. For multiple random sequences, the proportion of sequences passing the test is usually different but close to $1 - \alpha$. There is a high probability that the value of such a proportion should fall within a specific numerical range around the value $1 - \alpha$. NIST STS determines the range of acceptable proportions using the $\alpha$ significance level (0.01 by default) and the *k* number of the tested sequences:

8 $$\begin{aligned} 1 - \alpha \pm 3 \sqrt{\frac{\alpha \left( 1 - \alpha \right) }{k}}, \end{aligned}$$

For the value $\alpha$ = 0.01 and number of tested sequences *k* = 1000 the value of the proportion should fall into the interval 0.99 ± 0.0094392.

**Uniformity of***p***value distribution** Values of *P* calculated in a single test should be evenly distributed over the interval $\left[ 0,1\right)$. Hence, the uniformity of the *p* distribution formulates a hypothesis that can be verified with a statistical test. NIST STS uses the $\chi ^2$ single-sample test to evaluate the uniformity of the distribution of *p* values. The $\chi ^2$ test measures whether the observed discrete distribution (histogram) of a certain feature corresponds to the expected distribution. In NIST STS the interval $\left[ 0,1\right)$ is divided into 10 sub-intervals and the test $\chi ^2$ checks if the number of *p* values for each of the sub-intervals is close to the value of *k*/10 (for $k = 1000$, $k / 10 = 100$). The value in the ’P value’ column in the result table corresponds to the result (in the form of *p* value) of the test of the uniformity of the distribution of *p* values.

**Statistical test results** Partial results from finalAnalysisReport.txt files (NIST STS result files with a summary of the tests performed) for three test groups—100 MB samples are presented below (cf. Table 4 for Comscire, Table 5 for JUR01, Table 6 for Mathematica). Each row of the result set corresponds to one test (or sub-test). The values in columns C1, C2, ..., C10 represent the number of single values of *p* that fall between the ranges of values [0.0, 0.1), [0.1, 0.2), ..., [0.9, 1.0). The value in the column “P value” means the test result of the uniformity of the distribution of *p* values calculated for the given test. The value in the column “Proportion” means the proportion of sequences that passed the test. Results interpreted by NIST as non-random are marked with an asterisk.

**Table 4:** Quantum generator Comscire (test sequence of length 100 MB, tested *k* = 1000 sequences, each consistent of *n* = 800 000 bits), by * (number of performed sub-tests) indicated averaged proportions and values of *p* for uniformity distribution test for results related to particular sequences.

C1	C2	C3	C4	C5	C6	C7	C8	C9	C10	Value P	Proportion	Test
101	111	99	117	103	90	85	102	78	114	0.112047	994/1000	Frequncy
103	108	108	112	92	91	95	90	103	98	0.775337	992/1000	Frequency in block
91	115	113	100	112	95	106	96	77	95	0.186566	994/1000	Increasing sums (2*)
98	90	123	108	99	98	100	95	100	89	0.486588	990/1000	Courses
93	101	100	110	101	116	84	95	101	99	0.647530	990/1000	Courses in block
78	94	104	107	109	106	90	118	97	97	0.246750	991/1000	Matrices
102	111	92	103	97	84	121	85	93	112	0.152902	988/1000	Spectral
95	101	96	90	110	96	111	106	108	87	0.691081	990/1000	Nonoverlappnig patterns (148*)
124	112	96	85	114	103	86	81	94	105	0.037076	980/1000	Overlapping patterns
124	103	112	113	97	68	89	92	93	109	0.007530	984/1000	General
91	89	109	114	93	110	89	111	95	99	0.440975	991/1000	Entropy
48	61	63	51	54	67	71	59	48	43	0.141976	559/565	Excursions (8*)
49	48	67	48	61	49	78	58	57	50	0.066510	561/565	Variational excursions (18*)
122	102	96	88	96	90	87	121	95	103	0.119508	990/1000	Series (2*)
113	106	101	110	96	96	94	106	84	94	0.635037	989/1000	Linear complexity

**Table 5:** Quantum generator JUR01 (sequence of length 100 MB, tested *k* = 1000 of sequences, each consistent of *n* = 800 000 bits).

C1	C2	C3	C4	C5	C6	C7	C8	C9	C10	Value P	Proportion	Test
1000	0	0	0	0	0	0	0	0	0	0.000000*	0/1000*	Frequency
527	172	103	65	41	34	19	18	14	7	0.000000*	845/1000*	Frequency in block
1000	0	0	0	0	0	0	0	0	0	0.000000*	0/1000*	Increasing sums (2*)
1000	0	0	0	0	0	0	0	0	0	0.000000*	0/1000*	Courses
107	104	91	94	118	108	93	90	106	89	0.459717	985/1000	Courses in block
104	82	108	108	85	106	98	106	105	98	0.536163	994/1000	Matrices
115	105	92	87	97	104	104	96	95	105	0.749884	991/1000	Spectral
726	106	66	41	22	12	15	7	4	1	0.000000*	631/1000*	Nonoverlapping patterns (148*)
149	106	107	102	94	100	69	90	92	91	0.000023*	984/1000	Overlapping patterns
633	122	62	45	39	23	23	12	23	18	0.000000*	723/1000*	Universal
1000	0	0	0	0	0	0	0	0	0	0.000000*	0/1000*	Entropy
0	0	0	0	0	0	0	0	0	0	–	–	Excursions (8*)
0	0	0	0	0	0	0	0	0	0	–	–	Variational excursions (18*)
350	52	52	42	52	59	35	49	51	51	0.000000*	519/1000*	Series (2*)
87	102	104	109	100	94	86	107	99	112	0.641284	990/1000	Linear complexity

**Table 6:** pseudorandom generator from Mathematica (sequence 100 MB, tested *k* = 1000 of sequences, each consistent of *n* = 800 000 bits).

C1	C2	C3	C4	C5	C6	C7	C8	C9	C10	value P	Proportion	Test
88	93	109	93	97	100	115	115	107	83	0.262249	994/1000	Frequency
98	100	87	104	105	96	101	112	100	97	0.921624	990/1000	Frequency in block
83	110	98	106	100	117	88	103	100	95	0.440975	993/1000	Increasing sums (2*)
102	95	97	102	95	99	106	96	120	88	0.674543	994/1000	Courses
114	108	107	111	99	104	99	92	84	82	0.281232	984/1000	Courses in block
87	89	121	121	114	107	95	87	81	98	0.019453	992/1000	Matrices
114	120	83	102	86	95	104	107	94	95	0.193767	989/1000	Spectral
102	93	86	93	109	101	118	89	98	111	0.392456	990/1000	Nonoverlapping patterns (148*)
122	106	98	115	98	92	89	101	79	100	0.129620	990/1000	Overlapping patterns
96	98	99	100	108	112	95	78	119	95	0.259616	989/1000	Universal
115	118	117	84	90	79	97	106	89	105	0.032274	987/1000	Entropy
59	43	59	60	66	71	64	63	56	51	0.387323	586/592	Excursions (8*)
55	60	51	53	53	64	62	66	60	68	0.799089	587/592	Variational excursions (18*)
110	112	99	96	107	89	82	96	111	98	0.440975	989/1000	Series (2*)
98	105	80	104	103	116	116	95	85	98	0.202268	990/1000	Linear complexity

Both the Comscire commercial quantum generator and the Mathematica pseudorandom generator passed the randomness tests (all 15 tests of uniformity of distribution and 15 tests of proportion for the samples from each generator passed). Both generators can then be considered as generating random sequences in the light of NIST’s statistical tests. It’s worth noting that the results for both generators are similar—the proportion test results for each test are around 99%. It is difficult to notice significant differences between the two generators (quantum and pseudorandom) in the analysis of the NIST STS results, which may indicate the fact that during the statistical testing of random sequences it is not possible to detect true randomness.

In the case of the current version of the JUR01 quantum generator, more than 7 tests for the first sample have failed which, according to both the original NIST interpretation and the interpretation proposed by^51^, proves that the generator is non-random, unless the whitening procedure is applied to the row sequence (not hardware implemented in JUR01, however).

Tests for 10 MB samples in the case of the QRNG Comscire and PRNG Mathematica generators gave results indicating non-randomness, which were considered as statistical errors related to too few tested sequences—the results were not presented, the results were focused on the larger 100 MB samples.

The above examples demonstrated that the NIST’s test is too weak to distinguish between pseudorandom classical sequence and true quantum random sequence, at least at the tested sequence length of 100 MB. This test was able to detect a bias, however. It actually does it in the case of the second tested QRNG JUR01. The generator JUR01 is a row electronic implementation without any hardware bias reduction of bias by whitening methods installed. Application of the simple von Neumann algorithm (it considers two bits at a time (non-overlapping), taking one of three actions: when two successive bits are equal, they are discarded; a sequence of 1,0 becomes a 1; and a sequence of 0,1 becomes a zero—it thus represents a falling edge with a 1, and a rising edge with a 0—this eliminates simple bias, and is easy to implement as a computer program or in digital logic) occurs to be efficient in reducing bias, and with the cost of reduction of the sequence length, the improved sequence passed successfully NIST’s test. We suspect that any QRNG needs the reduction of an unavoidable bias linked to technical implementation and such a procedure is hardware implemented in offered QRNGs, including Comscire.

### Toward miniaturization

**Figure 4:**
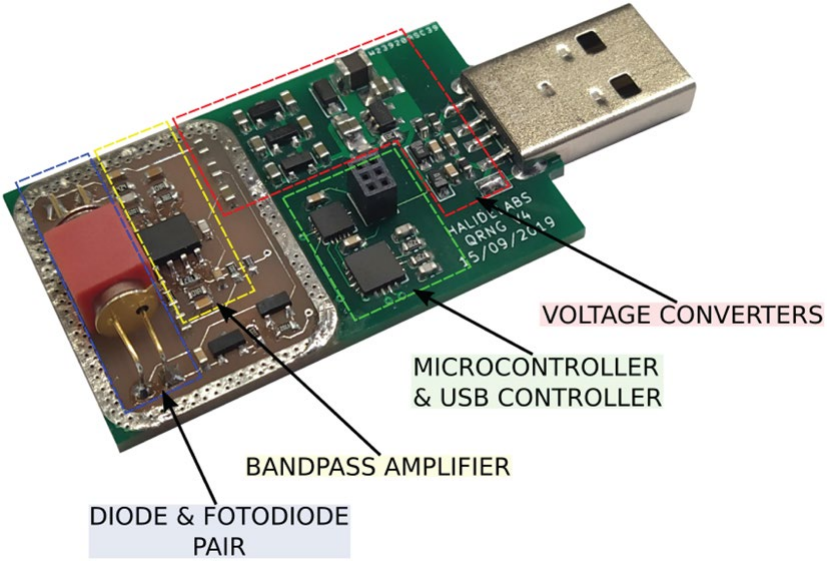
Prototype of miniaturized version of QRNG JUR02 designed at WUST (2020) – it passed all NIST/Dieharder tests without the bias reduction, at the speed of entropy creation 1 Mb/s (configured with conventional USB controller).

Hardware realization of QRNG meets with growing needs to implement advanced cryptosystems (including Quantum Key Distribution systems^34–36,53^) for future Internet and communication security. QRNGs will be in near future built-in personal computers and even in mobile devices. Thus miniaturization of QRNGs is required. The next step in project Jurand (after the first prototype JUR01) was the prototyping of the following model of QRNG operating on the basis of Fermi golden rule, by exploiting, as the source of the entropy, the photoelectric process in a photodiode coupled to a small LED. The size of the construction has been reduced to $28\times 10\times 46.5$ mm and moreover, a few millimeter integrated circuit (to be next developed) has been also designed, which can be easily incorporated into mobile phones and portable computers. Remarkable, the prototype (as shown in Fig. 4 and in Supplementary Information F) producing entropy with the speed 1 Mb/s passed the NIST and Dieharder tests without bias reduction (which evidences that at such photovoltaic source of the entropy the possible bias is low).

The small QRNG has been presented in 2020 upon the project NCBiR, POIR.01.01.01-00-0173/15. The prototype called JUR02 is miniaturized to the box of size $28\times 10\times 46.5$ mm and integrated with conventional USB port allowing for universal easy application in personal computers—cf. Fig. 4. JUR02 successfully passed NIST SP-800-22 and Dieharder v. 3.31.1 tests. Testing using the package Linux-ent gives

- Optimum compression would reduce the size of this 2064385896 byte file by 0%
- Chi square distribution for 2064385896 samples is 255.90, and randomly would exceed this value by 47.23%
- Arithmetic mean value of data bytes is 127.5014 (127.5 = random)
- Monte Carlo value for $\pi$ is 3.141579925 (error 0.00%)
- Serial correlation coefficient is $-0.000035$ (totally uncorrelated = 0.0)

**Figure 5:**
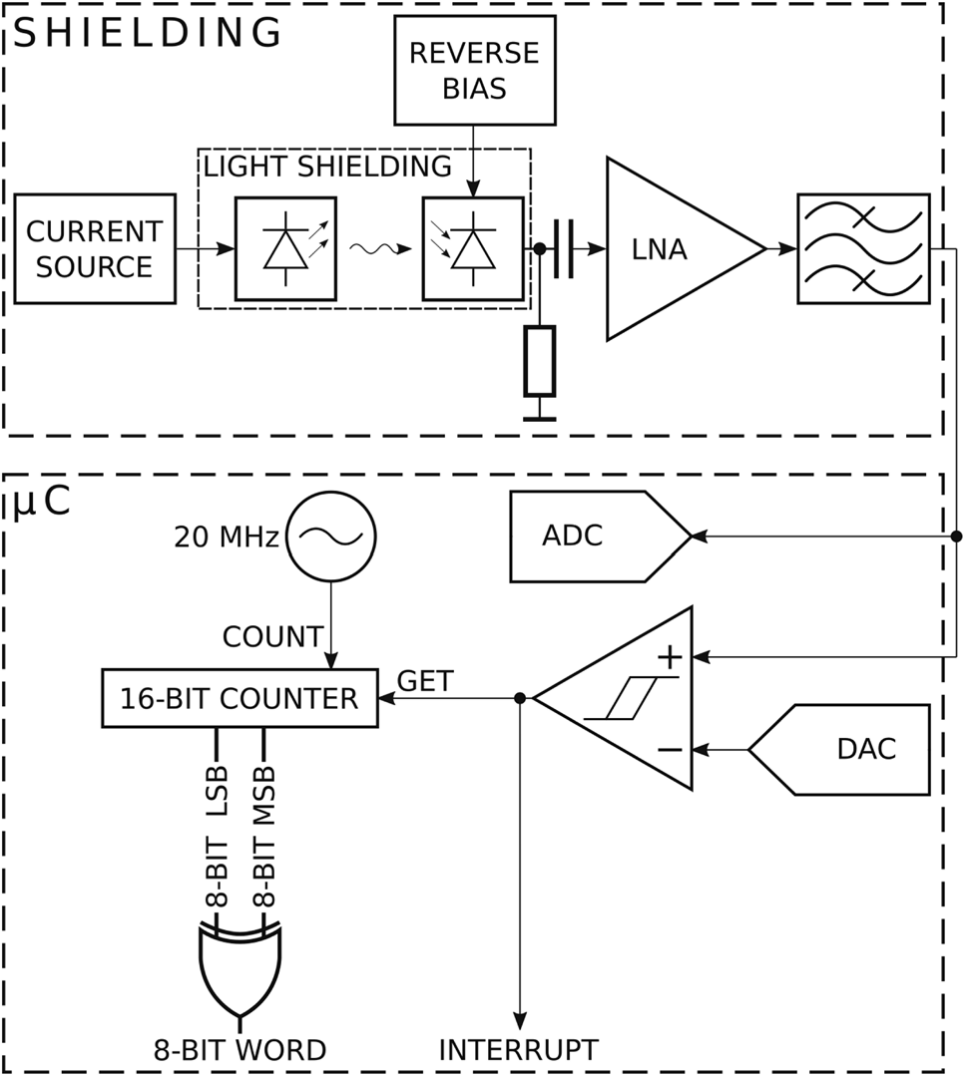
Simplified block scheme of JUR02.

**Figure 6:**
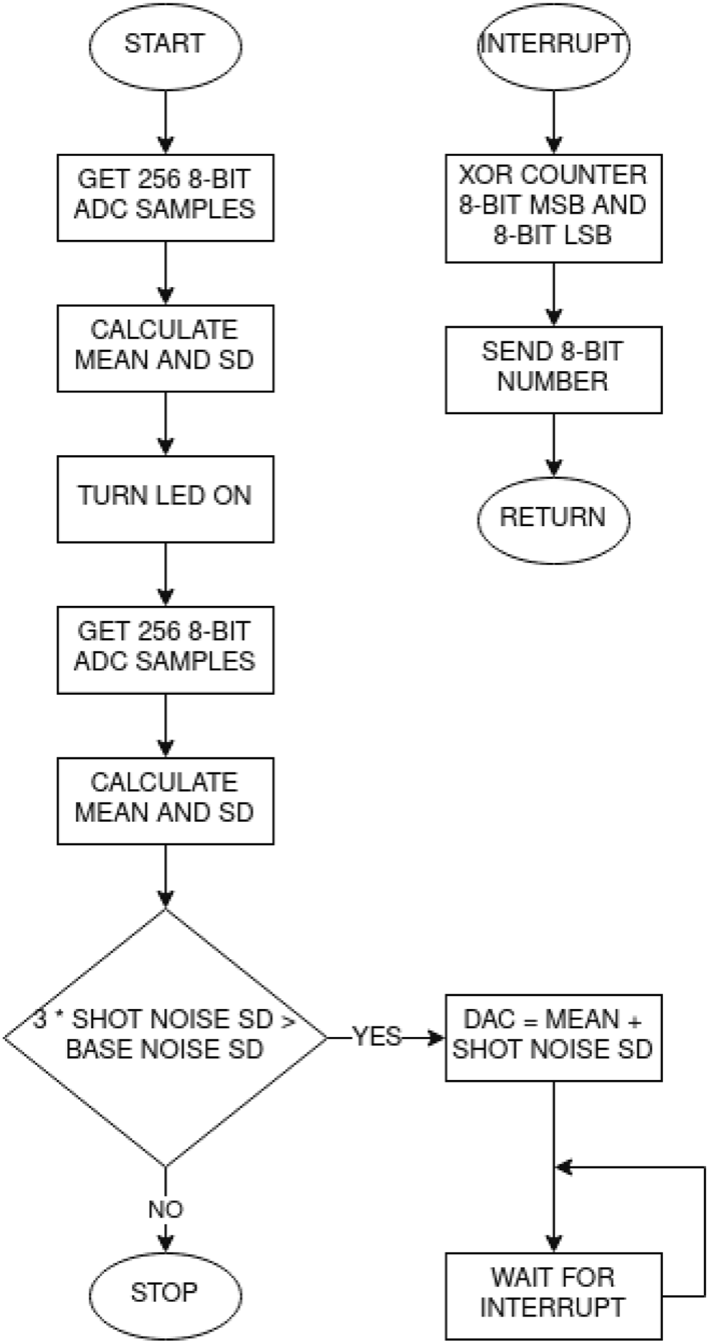
The scheme of the algorithm for automatic starting and calibration sequence and generating of random bit sequence implemented in JUR02.

The source of randomness in JUR02 is the shot noise in the photodiode and produces the random bit sequence at the time rate 1 Mb/s. The simplified block-scheme of JUR02 is shown in Fig. 5 and the scheme of starting sequence and the algorithm of random bit generation is visualized in Fig. 6. The exemplary data (2GB) from JUR02 are available at address https://halidelabs.eu/QRNG/data.bin.

## New concept of a entanglement-based QRNG protocol with public randomness verification

Irrespectively of the QRNG type, due to inevitable implementation imperfections, the fidelity of the quantum randomness extraction will always be not ideal (similarly as in other quantum information protocols, which are perfect only in theory, e.g., quantum key distribution, but imperfect when implemented). Whether in device independent (DI) QRNGs^54–56^, in self-testing DI approach-type QRNGs^57^, or in other QRNGs the separation of the classical component from the quantum can be done only up to a finite confidence level, and its verification can be reduced to statistical predictions (like statistical proofs of Bell, CHSH or Mermin type inequalities violation^24,58,59^, or similar to continuous variable approach statistical analysis^23^). Thus, it is crucial that quantum random number generation should always be accompanied by a classical randomness verification procedure as comprehensive as possible^60^.

It is important to emphasize, that a detailed randomness testing is a task of considerable computational complexity, especially testing for the existence of long-range correlations (i.e. verifying the deviations of the frequency of occurrences of long patterns, which could manifest potential biases, as standard tests are focused rather on short-range correlations). Basically, the reason for that complexity is an exponential increase of the number of possible testing patterns with the increase of those patterns length. The concept of an ideal randomness can only be used in the case of an infinite sequence, but even so, without a formal mathematical definition, due to the uncountable number of all possible testing patterns. But in the case of finite sequences, one can define a complete testing in the simplistic manner (surely not an optimal one) as a verification of deviations, from the expected values, of the frequency of occurrences of all the patterns with length not exceeding the length of the tested sequence. For each sequence of the length *m*, the expected value of the frequency of occurrences is determined on the basis of the statistics of an infinite sequence, i.e. $\frac{1}{2^m}$. For the tested sequence of the length *n*, the number of all tested patterns, not longer than *n*, is equal to $\sum _{k=1}^n 2^k$. The exponential growth is clearly visible, same as the exponential growth of the computing resources requirements, to keep the testing procedures in the regime of effective execution times. What is crucial here is that typically, the QRNG itself (or even together with the control unit) has a small amount of computing resources, allowing to perform locally a strictly limited tests scope in realistic execution times.

Recently Cambridge Quantum Computing together with IBM announced a launch of the world’s first cloud-based Quantum Random Number Generation Service with integrated verification for the users (initially intended for the members of the IBM Q Network)^61,62^. This service is implemented on the IBM Quantum computers network, claimed to be device independent quantum machines in a verifiable manner allowing to certify the generated randomness. Such verification procedure is a statistical analysis of the underlying processes of quantum randomness generation with the use of the Bell test based on the Mermin inequality, but with an assumption of complete shielding of user’s facility (including the quantum device) from the outside once the protocol starts^62^. Such situation is highly restricting due to two main reasons:

1. it requires that user must be equipped with the IBM Quantum Computer with Qiskit module qiskit_rng^63,64^, otherwise, in case of claimed to be cloud-based QRNG service, user have to trust the service vendor, as the vendor has full access to generated random sequences, which user would want to use cryptographically;
2. the classical statistical analysis, in the case of a high level of confidence, could require huge amount of computational resources (unavailable locally) to be completed in realistic time.

The concept of entangled QRNG protocol with public verification^60^ is free of such limitations due to unique features, like unconditionally secure public randomness testing, overcoming local computational restrictions, or diminishing of the average time of the complex randomness testing of finite length bit sequence. In this protocol the randomness of the generated single sequence proves the randomness of all the other simultaneously generated sequences (or the randomness of the shorter sequence proves the randomness of longer sequence), which is a crucial result of multi-qubit quantum entanglement involved^60^. The idea of the protocol is briefly described below.

The protocol uses a multi-qubit entangled state (in computational basis $\left\{ 0,1\right\}$), on which quantum measurements (of ${\hat{\sigma }}_z$ operator) are performed. $k+2$ entangled qubit state, in a form as below, is required to obtain *k* secure and publicly verifiable sequences.

9 $$\begin{aligned} \begin{aligned} \left| \Psi _{Q_1\ldots Q_{k+2}}\right\rangle = 2^{-\frac{k+1}{2}}\left( {\prod _{i=1}^{k+1}}^{\left( \otimes \right) }\sum _{q_i = 0}^1\left| q_i\right\rangle \right) \otimes \left| q_1\oplus q_2\oplus \ldots \oplus q_{k+1}\right\rangle \end{aligned} \end{aligned}$$

where $q_1,\ldots ,q_{k+1}$, in the last ket of each sum element, are valued according to $k+1$ first kets in that element; $\oplus$ is the sum modulo 2.

Steps of the protocol in the ideal case are as follows (the not ideal case is discussed in details in^60^)

**Figure 7:**
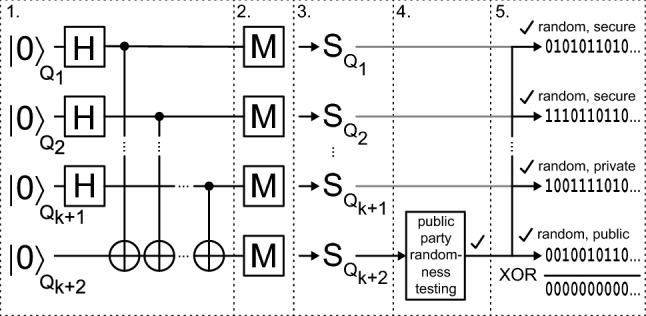
Protocol of the multiqubit, entanglement based, cryptographically secure QRNG with public randomness verification^60^.

1. Preparation of the initial state (as in eq. (9)) in the form of the uniform sum of such kets, that each of them has identical sum modulo 2 of every single qubit states defining that ket – the so-called XOR rule valued 0 (cf. Fig. 7 1.)
2. Local measurements of each qubit (cf. Fig. 7 2.)
3. *n* times repetition of steps 1. and 2. to obtain *n*-bits long sequences, $S_{Q_1},S_{Q_2},\ldots ,S_{Q_{k+1}},S_{Q_{k+2}}$, corresponding to the measurement results of the qubits $Q_1, Q_2, \ldots , Q_{k+1},Q_{k+2}$ accordingly. The so-called XOR rule (here valued 0) is formulated as the sum modulo 2 of all qubit measurement result values (in the i-th measurement series) must be equal equal 0, $S_{Q_1}^{\left( i\right) }\oplus S_{Q_2}^{\left( i\right) }\oplus \ldots \oplus S_{Q_{k+1}}^{\left( i\right) }\oplus S_{Q_{k+2}}^{\left( i\right) } = 0$ (cf. Fig. 7 3.)
4. Public randomness testing of only a single sequence from set $S_{Q_1},S_{Q_2},\ldots ,S_{Q_{k+1}},S_{Q_{k+2}}$ (in result this is equivalent to simultaneous public randomness testing of all of the sequences but without compromising their secrecy), by publicly announcing a sequence in order to verify its randomness by a third party (with arbitrary large computational resources). Due to a specific quantum entanglement of initially measured qubits the single sequence testing result will also concern all the unpublished sequences (cf. Fig. 7 4.)
5. After a successful randomness verification all the remaining sequences are also truly random and all but one (here, *k* sequences) can be used cryptographically (one sequence must never be used or published to ensure the secrecy of the remaining generated sequences, due to the XOR rule) (cf. Fig. 7 5.)

In the ideal case, due to the quantum entanglement all the sequences of measurement results, $S_{Q_1}, S_{Q_2}, S_{Q_3}, \ldots$ share the same statistical properties—the deviations of frequencies of occurrences in sets of patterns of the same length are identical for all of those sequences in the limit of sequences length *n* tending to infinity. Irrespective of the number *k* $\left( k > 2\right)$ of entangled qubits $Q_1, Q_2, Q_3, \ldots , Q_k$, a successful verification of randomness of only a single sequence $S_{Q_i}$ proves the randomness of all $k - 1$ remaining sequences.

Randomness verification of the sequence $S_{Q_i}$ can be performed publicly, leaving the secrecy of remaining sequences ($k - 1$) completely intact, provided that another single sequence (from the remaining sequences) $S_{Q_j}, j \ne i$ will be kept in secret and never be used—which leaves $k - 2$ secret sequences with the randomness proven by the sequence $S_{Q_i}$ randomness verification result and ready for cryptographic or any other usage.

Public testing allows to perform an arbitrary complex testing (up to the verification of deviation from statistical prediction of occurrences of all possible patterns for *n*-bit tested sequence, which is a very challenging task in terms of computational resources) overcoming the strong restrictions of computational resources nature of the local randomness testing possibilities of the QRNG controlling unit or of the QRNG itself. However, public testing should be performed by a trusted party, or as a service within a reputation based model (then the trust is based on the reputation and service usability), e.g., one with a blockchain type public testing results database, which will be discouraging to falsify tests results (reputation loss for unhonest verifiers), and encouraging to test faster and more accurate (reputation gain for honest verifiers).

Another crucial feature of this protocol is a diminishing of an average time of the complex randomness testing (which in the case of e.g., finding patterns, the execution times grows exponentially with the increase of the length of searched patterns) of finite length bit sequence. With the increase of the number of entangled qubits, the number of secret random bit sequences also increases. All of those sequences hold the same statistical properties (due to the nature of the proposed protocol)—it is sufficient to test only a single sequence to get the information about the randomness of all other sequences. As the time needed to test a single sequence is fixed (it depends on the sequence length and does not change with the increase of entangled qubits), thus the average time (single sequence time divided by the number of sequences sharing the same statistical properties) can be brought to arbitrary small value in theory.

Moreover, the generated sequences can be concatenated into a one long sequence, whose length corresponds to the number of entangled qubits, and its randomness will still be proven by the randomness of a short single sequence of the initial length. In other words, with the increase of the number of qubits composing multi-qubit entanglement the complexity of the randomness testing decreases. Hence, with the same amount of the computational resources one can test much longer sequences (in the infinite limit of entangled qubits number the randomness testing, in this scope, becomes trivial)^60^. This interesting observation seems to shed a new light on how to understand fundamental theoretical concepts behind recently reported quantum supremacy for the randomness testing with use of multi-qubit entanglement.

The recent reports on the programmable entanglement based processor Sycamore^21^ allows to propose its usage for quantum acceleration of classical randomness testing. Sycamore obtained the quantum supremacy title, which in fact was later denounced by IBM^65^ – nevertheless, the exponential growth of the time needed to simulate Sycamore classically, with the increase of quantum gates and qubits involved was sustained (currently the quantum supremacy title belongs to Chinese photonic quantum computer Jiuzhang^22^). The Google’s processor allows to freely choose the order and the type of one-qubit gates ($\sqrt{X}, \sqrt{Y}, \sqrt{\frac{X+Y}{2}}$, as exemplary presented by Google’s team^21^) applied in layers to each qubit and the patterns type of alternating swapping of neighboring qubits (with the use of iSWAP gates), locating the operations in the quantum supremacy regime or not. It is possible to employ those degrees of freedom to perform a classically unattainable randomness testing. One can introduce consecutive parts of the tested sequence as the choosing keys for single-qubit gates layers defining subsequent specific quantum circuit configuration. Next for each of such configurations a random sampling procedure can be performed (multiple execution with the following quantum measurements to obtain statistical distribution of possible quantum states resulting from that specific configuration). Afterwards, all the obtained ket distributions (squared amplitudes of kets from all $2^{53}$ possible kets) from each configuration, can be merged together, resulting in a pattern correlated with the tested sequence. As it is possible to shuffle the qubits numbering for each configuration (to ensure the lack of distinguishability between all qubits – due to the qubits located on the boundaries of the grid), in case of a random tested sequence, one should expect the uniformity of the obtained pattern, and any deviations should indicate that the tested sequence is not random. For this test to be effective (beyond the simple testing of the patterns occurrences), the parts of tested sequence should be long enough to exceed the number of degrees of freedom of the output distribution – the Sycamore processor would then operate as a specific type of a hash function (the measurement of qubits irreversibly destroys mutual phase shifts between kets in a specific mixture of the all $2^{53}$ possible kets). Due to the quantum supremacy regime, such procedure would not be possible to be calculated effectively in a classical manner. Such usage of the Sycamore or Sycamore type processor would constitute a great tool for a public institution offering an open randomness testing service in the model of entangled QRNG with public randomness verification protocol.

## Conclusion

In this paper, we emphasize the key role of the unconditional randomness of quantum random number generators in contrast to pseudorandom classical generators. We identify quantum ideal sources of entropy in the randomness of quantum measurements according to the von Neumann scheme, and innovatively in quantum transitions based on the so-called Fermi golden rule. The latter source of the entropy is extremely useful in modern constructions of very fast and efficient quantum random number generators. We have proposed two of our own prototypes of such quantum random number generators, based on the Fermi golden rule. The first one uses the tunneling current in the Zener diode, and the second one uses the shot noise in a photodiode illuminated by LED. However, in every case of quantum randomness generator practical implementation, there exists some classical component/admixture, causing a bias in the resulting random sequence. In the first prototype of ours, the bias was removed by the von Neumann algorithm for whitening the random bit sequence. In the second case, the proposed system gave a negligible bias, and the random sequence, generated at the velocity rate of 1 Mb/s, successfully passed all randomness tests in NIST and Dieharder batteries.

We also emphasize that the currently used randomness tests are not ideal, and have difficulties in distinguishing pseudorandom sequences from truly random ones (what we have demonstrated with selected examples). Achieving greater randomness testing precision is a task, however, highly consuming computing resources, which are not always locally available to a sufficient extent. Any external, public testing is destructive for the security of the tested bit sequence, effectively preventing its cryptographic use. To circumvent this difficulty, we propose a new algorithm for the operation of a quantum random number generator with world’s first scheme of the non-destructive public randomness testing. The concept uses multiqubit quantum entanglement (at least 3-qubit entanglement), and allows any external party to publicly test the randomness, with an arbitrarily high accuracy, of only a single bitstream component, while maintaining a complete confidentiality of the other bitstream components (their number grows with the initial number of entangled qubits), however, sharing the same fidelity level (identical statistical correlations), with regard to their randomness quality as the one published for testing. Such approach overcomes the highly restricting local computational resources limitations of randomness testing procedures, and it allows to lower in average the overall time needed for an arbitrarily complex randomness testing.

## Supplementary information

Supplementary material 1 (pdf 2111 KB)
