# Supplementary material for: Quantum generators of random numbers
Source: Sci Rep. 2021 Aug 9;11:16108. doi: 10.1038/s41598-021-95388-7 (PMC8352985; doi:10.1038/s41598-021-95388-7)
Supplement: Supplementary file 1 — Supplementary material 1 (pdf 2111 KB) [file 41598_2021_95388_MOESM1_ESM.pdf]

SUPPLEMENTARY INFORMATION

Quantum generators of random numbers

Marcin M. Jacak[1,+], Piotr Jóźwiak[2,+], Jakub Niemczuk[1,+], and Janusz E. Jacak[3,*,+]

[1]Wrocław University of Science and Technology, Wyb. Wyspiańskiego 27, 50-370 Wrocław, Poland
[2]Faculty of Computer Science, Wrocław University of Science and Technology, Wyb. Wyspiańskiego 27, 50-370 Wrocław, Poland
[3]Department of Quantum Technologies, Wrocław University of Science and Technology, Wyb. Wyspiańskiego 27, 50-370 Wrocław, Poland
[*]janusz.jacak@pwr.edu.pl
[+]these authors contributed equally to this work

Contents

A Mathematical definitions of the classical randomenss 2
A.1 The Von Mises Unpredictability . . . 2
A.2 Typicality of Laplace-Ville and Martin-Löf . . . 4
Randomness by Martin-Löf • Solovay randomness
A.3 Taking randomness in terms of computational complexity of Kolmogorov . . . 4
A.4 Kolmogorov-Chaitin's randomness . . . 5

B Differences between classical and quantum measurement – sources of entropy in quantum mechanics 6
B.1 Measurement in quantum mechanics . . . 6
B.2 von Neumann measurement . . . 6
B.3 Unitary evolution . . . 6
B.4 Quantum transitions – the source of entropy for QRNGs . . . 7
B.5 Fermi golden rule . . . 7

C Commercial QRNGs overview 8
C.1 IDQ Quantis Random Number Generator . . . 8
C.2 ComScire PureQuantum . . . 9
C.3 Toshiba UFICS-QRNG . . . 9
C.4 PicoQuant PQRNG 150 . . . 10
C.5 Whitewood Entropy Engine . . . 10
C.6 QuintessenceLabs qStream . . . 10
C.7 QuantumNumbersCorp QNG2 . . . 11
C.8 EYL Micro Quantum Random Number Generator . . . 11
C.9 qutools quRNG . . . 12
C.10 Micro Photon Devices Quantum Random Number Generator . . . 12
C.11 QuantumCTek Quantum Random Number Generator QRNG100E . . . 12
C.12 Quside FMC 400 . . . 13
C.13 Commercial QRNGs Summary . . . 13

D Popular test batteries or test suits for the statistical randomness verification 13
D.1 NIST Test . . . 13
D.2 Test U01 . . . 14
D.3 Diehard and Dieharder tests . . . 14

E Detailed description of the NIST battery tests 15
E.1 Test of the frequency of single 0 and 1 occurrence in the studied sequence . . . 15
E.2 Test of the frequency of single 0 and 1 occurrences in the block of the tested sequence . . . 16
E.3 Runs test . . . 17
E.4 Longest run of ones in the block test . . . 17
E.5 Binary matrix rank test . . . 18
E.6 Spectral test – Discrete Fourier transform test . . . 19
E.7 Test for occurrence of non-overlapping patterns . . . 19
E.8 Test for Overlapping Patterns . . . 21
E.9 Universal statistical Maurer test . . . 24
E.10 Linear Complexity Test . . . 25
E.11 Serial test – pattern distribution test . . . 26
E.12 Entropy Estimation Test . . . 27
E.13 Test of cumulative (rising) sums . . . 28
E.14 Random excursion test . . . 29
E.15 Variant random excursion test . . . 32
F New prototype of QRNG upon the project Jurand, JUR02 32
References 34

A Mathematical definitions of the classical randomenss

A.1 The Von Mises Unpredictability

The theory presented by von Mises in 1919[1] proposed one of the principles of the theory of probability, but its foundations were not purely mathematical as they were defined by a physical experiment. This rule was based on the so-called collective property of a random sequence.

Suppose S is a random experiment in which all possible results produce a finite set of $L = \{\alpha_1, \ldots, \alpha_m\}$ called the tagging set (attribute) of this experiment. The finished sample of the results of this experiment can be defined as,

$$x = (x_1, \ldots, x_N), \quad x_i \in L, \tag{1}$$

where N is the sample size. The collective is defined as the infinite number of realization of the experiment S, which is written as follows:

$$x = (x_1, \ldots, x_N, \ldots), \quad x_i \in L, \tag{2}$$

for which these expressions are true provided two assumptions:

1. Statistical stabilization of the relative frequency of the occurrence of each $\alpha \in L$ attribute (i.e., the possible result of the S experiment) in the above defined infinite sequence, where the frequencies are defined as follows:

$$\nu_N(\alpha, x) = \frac{n_N(\alpha, x)}{N}, \tag{3}$$

where $n_N(\alpha, x)$ is the number of α occurrences in the first N trials. Statistical stabilization is defined as the approximation of the relative frequency to a specified limit for each α, which is called the α attribute probability of a random S experiment:

$$P_x(\alpha) = \lim_{N\to\infty} \nu_N(\alpha, x). \tag{4}$$

2. Randomness of the sequence – which is defined by the statement that the statistical stabilization should be satisfied for the appropriate selection of the sub-sequence from x. The construction of the sub-sequence is based on the decision whether to accept or reject the n-th element of the original sequence – this decision cannot depend on the value of α and cannot be based on the value of x_n or any value in x_i where $i > n$. Therefore, the above selection can be described as follows:

$$f_1, f_2(x_1), f_3(x_1, x_2), \ldots, f_n(x_1, \ldots, x_{n-1}), \ldots, \tag{5}$$

where f_i returns 0 to reject and 1 to accept i-th element, so there must exist an infinite number of indices i for which f_i returns 1. The randomness condition states that there is no strategy that satisfies the selection rules and returns a sub-sequence with a different probability than the sequence generated by tossing the perfect coin (an eagle in the n-th coin toss preserves the x_i element). The sub-sequence selection is referred to as the site selection.

For example, for $L = \{0,1\}$ let x be the collective – a random sequence. Let n_1 be the first index for which $f_{n_1}(x_1,\ldots,x_{n_1--1}) = 1$, n_2 be the next such index, etc., then from the randomness condition one can write:

$$\exists_{x_{n_i}} \lim_{N\to\infty} \frac{1}{N} \sum_{i=1}^{N} x_{n_i} = \lim_{N\to\infty} \frac{1}{N} \sum_{k=1}^{N} x_k = P_x(1). \tag{6}$$

Von Mises never solved the problem of the existence of a random (collective in its nomenclature) sequence. But one can recall some definitions of collectivity pertaining to the special selection classes defined by certain rules.

- Bernoulli Sequences, which von Mises found inadequate to fully define collectivity, may be considered collective under very specific rules for choosing a place[1] – are defined as follows. Let $\omega_k = y_1 \ldots y_k$ be a k character word, i.e., the sequence k integers. For any word ω_k, the sequence $x \in 2^{\omega}$ is k-assigned if the probability P that any sub-sequence $x_n \ldots x_{n+k-1}$ of x is identical to the word ω_k is $\frac{1}{2^k}$, as follows:

$$P\left(x_n \ldots n_{n+k-1} = y_1 \ldots y_k = \frac{1}{2^k}\right). \tag{7}$$

  Bernoulli's sequence is a sequence that is ∞-assigned, i.e., k-assigned for each positive integer k. This definition can be understood as counting the relative frequency of the occurrence of any word in the analyzed sequence, which should coincide with randomness.

- Mises-Wald Collectivity – Wald in his work[1] formulated the theorem that for any countable set U of place choices and for any probability distribution p on the set of L tags, the set of sequences $X(U;p)$ has a cardinal number equal to the continuum, where

  - U – a family of place choices
  - $X(U;p) = \left\{x \in L^{\infty} : \forall_{\phi \in U} \lim_{N\to\infty} \nu_n(\alpha_j;\phi x) = p_j, j = 1,\ldots,m\right\}$
  - $\nu_N(a,y), \alpha \in L$ – the relative frequency of the occurrence of the α tag from the L set in the first N elements of $y \in L^{\infty}$
  - L^{∞} – infinite sequence of tags from the L set. In this formulation, Wald's random sequence is based on an arbitrary but countable set of place selections.

- Mises-Church Collects – Church[1] proposed to use select functions that can be algorithmized – only recursive ϕ_r functions are allowed in the von Mises definition. The set of such place choices is countable[1]. The results obtained by Wald justify the existence of algorithmically computable choice functions proposed by Church. It should be pointed out, however, that again in the light of von Mises's definition, such placement properties are too restrictive.

- Axiomatization of Lambalgen Collectives – Lambalgen has proposed[1] an approach of so-called axiomatization of the relative independence of sequences. The suggestion was to choose between accepting or rejecting i-th value of the binary representation of $x \in 2^{\omega}$ based on i-th digit in yet another sequence $y \in 2^{\omega}$.

While the presented definitions of the collectivity (randomness) seem to complete von Mises's theory, Ville has published a serious critical argument[1]. Ville found that if the set of markings $L = \{0,1\}$ and the countable set of place choices $U = \{\phi_n\}$, then

$$\exists_{x\in L^{\infty}} \begin{cases} \forall_n \lim_{N\to\infty} \sum_{j=1}^{N} (\phi_n x)_j = \frac{1}{2}, \\ \forall_N \sum_{j=1}^{N} (\phi_n x)_j \geq \frac{1}{2}, \end{cases} \tag{8}$$

By definition, such a sequence x is the collective, $x \in X\left(U;\frac{1}{2}\right)$, but cannot be considered random.

A.2 Typicality of Laplace-Ville and Martin-Löf

As Ville's objection to the von Mises theory proved that von Mises-Wald collectivity in general does not meet the criteria of randomness, he showed (which also contradicts Laplace's concept[1]) an alternative concept of collectivity, taken as a dual definition of randomness to the von Mises approach, based on the following idea: The random sequence should satisfy all probability properties 1 – these properties are the laws of probability theory: $\mu(\{x \in 2^{\omega} | A(x)\}) = 1$, where μ is a normalized measure and A is an expression. As with the allowed place selection in the von Mises definition, the problematic statement here is "all A properties with probability 1". Each such property can be interpreted as a test of randomness – so a random sequence, according to Ville, is a sequence that passes all randomness tests. However, such a situation is not possible, since only countably many such properties can be selected, or otherwise, in the case of an uncountable family of sets, their cross-section may have a probability of less than 1 or even probability not defined at all. In other words, the random sequence defined within the selected probabilistic measures should satisfy all the probability laws for this measure, or the set of random sequences should be the result of the cross-section of all possible probability properties 1 – however, such a cross is an empty set, therefore the condition defined in this way is never met. The only way to avoid the indicated situation is to select only a part of such properties – i.e., a certain (countable) family of tests. This leads to ambiguity in the described approach, such as a selection can be made in many ways giving different families of tests. This problem was solved by Martin-Löf in 1970[1] by considering recursive, i.e., algorithmic, probability properties of 1, which led to the theory of recursive algorithmic testing of the randomness. However, despite the success in formally defining randomness, a paradoxical situation arises. It can be proved that in the language of algorithmic recursiveness there is a so-called universal algorithmic randomness test, which is a universal oracle that decides whether a sequence is truly random or not. Nevertheless, such a test cannot be constructed in an algorithmic manner and its form remains unknown. This leads to a situation where, for a given sequence, its randomness cannot be checked algorithmically, despite theoretical knowledge that a proper algorithmic test exists.

A.2.1 Randomness by Martin-Löf

Formally, the Martin-Löf's randomness of the real r is defined as follows:

$$\forall_i \mu(A_i) \leq 2^{-i} \rightarrow \neg\forall_i r \in A_i, \tag{9}$$

where A_i corresponds to the set of recursively enumerable infinite series of the interval sets.

A.2.2 Solovay randomness

Solovay proposed a definition without using the so-called the convergence of significant levels as opposed to the case of the Martin-Löf's definition. Solovay's randomness of the real number r is defined as:

$$\sum_i \mu(A_i) < \infty \rightarrow \exists_N \forall_{i>N} r \notin A_i. \tag{10}$$

In the case of von Mises's theory, collectivity is considered more important compared to statistical tests, while Ville treats statistical tests as the main factor of randomness – in the Martin-Löf's approach in order to confirm the randomness of the sequence, it is required that all computational statistical tests must be satisfied with probability 1. Such a situation takes place when the considered sequence cannot be algorithmically compressed to a shorter sequence – this sheds light on the correspondence or even the equivalence of the definition of randomness based on statistical arguments with computational complexity based on arguments.

A.3 Taking randomness in terms of computational complexity of Kolmogorov

Kolmogorov found that randomness is directly correlated with complexity and that this complexity must be verified algorithmically. One can formally define:

- $L = \{0,1\}$.
- word is a terminated sequence of symbols in L.
- L^* is the set of all words in the L alphabet.
- the algorithmic complexity of the word x with respect to an arbitrarily defined algorithm A is defined as,

  $$K_A(x) = \min l(\pi), \tag{11}$$

  where $\{\pi\}$ is the set of all programs that can generate the word x using the A algorithm, $l(\pi)$ is the length of the program pi.

- $\forall_A \exists_{A_0} \exists_{C>0} K_{A_0}(x) \leq K_A(x) + C$.
- Kolmogorov's complexity $K(x_{1:n})$ of starting segments of x, $x_{1:n}$, is asymptotically convergent to n:

$$K(x_{1:n}) \sim n, \quad n \to \infty, \tag{12}$$

- conditional algorithmic complexity $K(x;n)$ is the length of the π program that generates the word x based on the fact that the word x is n

The Martin-Löf's theorem[1] states that for every sequence x in the alphabet $L = \{0,1\}$ the following expression is satisfied for infinitely many n,

$$K(x_{1:n};n) < n - \log_2 n. \tag{13}$$

This showed that the Kolmogorov random sequence, defined as $K(x_{1:n}) \sim n, \quad n \to \infty$, does not exist.

The Kolmogorov randomness as an algorithmic complexity has another drawback – it is not algorithmically computable, so the form of any optimal A_0 algorithm remains unknown. Nevertheless, it is possible to estimate this complexity, which is a solution to the above problem.

A.4 Kolmogorov-Chaitin's randomness

Despite the problems indicated above, it is possible to construct an improved definition of randomness based on the Kolmogorov concept[1].

Definitions:

- The algorithm is a computable (recursive) function $A : L^* \to L^*$ defined on some subset of D_A of L^*.
- Kolmogorov complexity of $x \in L^*$ in relation to the A algorithm is the length of the shortest program $\pi \in D_A$ such that,

$$A(\pi) = x : K_A(x) = l(\pi). \tag{14}$$

  In the case that the π program does not exist, then $K_A(x) = \infty$.
- The word prefix $x = x_1 \ldots x_n$ is written as $\hat{x} = x_1 \ldots x_m$ where $m \leq n$.
- The prefix-free D of L^* is a subset where no word in D is a prefix of any other word in D.

Suppose A is an arbitrary algorithm – i.e., a computable function, defined as a prefix of the free domain D. The prefix-free algorithmic complexity of the word x of L^* defined with respect to A is the length of the shortest program $\pi \in D$ such that

$$A(\pi) = x : \tilde{K}_A(A) = l(x). \tag{15}$$

Also, if such a program $\pi \in D$ does not exist, then $\tilde{K}_A(x) = \infty$. In this context, a theorem may be formulated that there exists an optimal prefix-free algorithm (computable function) A_0, such that,

$$\forall_A \exists_{C>0} \quad \tilde{K}_{A_0}(x) \leq \tilde{K}_A(x) + C. \tag{16}$$

Finally, the Kolmogorov-Chaitin random sequence x from L^∞, $L = \{0,1\}$ is a sequence that is uncompressible – none of its leading segments cannot be compressed by more than a certain finite number of bits. This property can be recorded as follows,

$$\forall_n \exists_{b>0} \quad \tilde{K}(x_{1:n}) \geq n - b. \tag{17}$$

The Kolmogorov-Chaitin randomness is equivalent to the Martin-Löf's randomness[1]. Both approaches are based on a well-developed formalism – in the case of Kolmogorov-Chaitin it is computational complexity and incompressibility, in the case of Martin-Löf it is typicality constituting the statistical basis for testing of pseudorandom sequences (e.g., in the scope of NIST standard tests). However, irrespective of the discussed theoretical foundations, both approaches do not seem to constitute a complete condition of the randomness. The concept of fundamental unpredictability seems to be closer to the essence of the randomness, despite the imperfections of formal attempts to formulate an appropriate description.

According to the arguments of Khrennikov and Zeilinger[1], it is possible that a purely mathematical approach to randomness and the formalization of its definition seems to be out of reach, as mathematical tools may be insufficient to formulate a theoretical framework for the concept of randomness. Perhaps it is rather physical processes that are the realm of reality in which there is true randomness beyond classical determinism in the area of quantum physics phenomena perceived as fundamentally nondeterministic.

B Differences between classical and quantum measurement – sources of entropy in quantum mechanics

To access the information, the measurement of its carrier is required. In the classic case, when the information carrier is a macroscopic system, the measurement does not change the system and can also be repeated. In the case of the quantum system, the measurement is destructive and cannot be repeated. In classical physics, the measurement is not destructive, it is repeatable. In the quantum case, this is impossible because a destructive measurement can only be performed once. Assuming the classical character of an observer, the information obtained as a result of measuring both classical or quantum information must be expressed as a real number, because only such an information is understandable by the consciousness of the classical observer. This condition causes that various results of the measurement, which can be distinguished in the classical gauge, must involve at least the Avogadro number of microscopic degrees of freedom at any measurement of the quantum system. Only then will the measurement results be macroscopically distinguishable, which is necessary for a classical observer. The quantum measurement results from the interaction of the measuring instrument with the system. The classical measurement does not take into account the interaction of the gauge device with the measured system, and therefore the classical measurement does not disturb the system. In the case of quantum measurements the situation is opposite – the interaction of the gauge device with the system is the essence of quantum measurement. In the result the quantum entanglement of the system with the gauge device takes place during the quantum measurement. Quantum entanglement meets the conditions of Schmidt representation[39], i.e., any quantum entanglement must be symmetrical — both the measured system and the instrument get entangled with each other in the same symmetrical step. There is no such effect in classical physics.

B.1 Measurement in quantum mechanics

In quantum mechanics, Hermitian operators in the Hilbert space of quantum states of a given quantum system are assigned to measurable quantities, they are called observables[40]. We chose the Hermitian operators as observables because the real character of their eigenvalues which can be interpreted as measurement results. For any Hermitian operator the spectral representation holds:

$$\hat{A} = \sum_n \lambda_n \hat{P}_n, \tag{18}$$

where λ_n is n-th eigenvalue of the operator $\hat{A}$, while $\hat{P}_n$ is projection operator onto the subspace in the Hilbert space corresponding to n-th eigenvalue of $\hat{A}$. The projection operator satisfies the properties:

$$\hat{P}_n^+ = \hat{P}_n, \tag{19}$$

i.e., is Hermitian operator, and

$$\hat{P}_n \hat{P}_m = \delta_{nm} \hat{P}_n. \tag{20}$$

B.2 von Neumann measurement

According to the von Neumann postulate (axiom), in quantum mechanics we assume that as a result of the measurement of the quantity A one gets only a single eigenvalue λ_{n_0} of that quantity, however, in an unpredictable manner which one. Simultaneously, the state $|\Psi\rangle$, which has been measured disappears and changes into one of the eigenstate of $\hat{A}$ corresponding to randomly selected eigenvalue – the measurement result. In advance it is known only the probability of this random selection,

$$p_{n_0} = ||\hat{P}_{n_0}|\Psi\rangle||^2 = \left\langle \Psi \middle| \hat{P}^+_{n_0)} \hat{P}_{n_0} \middle| \Psi \right\rangle = \left\langle \Psi \middle| \hat{P}_{n_0} \middle| \Psi \right\rangle, \tag{21}$$

while the wave function $|\Psi\rangle$ reduces during the measurement in an irreversible way (*collapses*) to a function (it is the so-called von Neumann collapse),

$$\frac{\hat{P}_{n_0}|\Psi\rangle}{\left\langle \Psi \middle| \hat{P}_{n_0} \middle| \Psi \right\rangle^{1/2}}. \tag{22}$$

The von Neumann projection is absolutely unpredictable and thus is considered as the entropy source for QRNGs.

B.3 Unitary evolution

Quantum evolution acc. to the Schrödinger equation for a closed system, $i\hbar\frac{\partial\Psi}{\partial t} = \hat{H}\Psi$ (where $\hat{H}$ is the Hamiltonian of the closed system) is the unitary evolution, $\Psi(t) = e^{-i\hat{H}t/\hbar}\Psi(0)$, which conserves information (and entropy). The unitary evolution

is deterministic in the Hilbert space – conserves the entropy $S = -Tr(\hat{\rho} ln \hat{\rho})$, where $\hat{\rho} = |\Psi\rangle\langle\Psi|$ is the density matrix for the pure state Ψ (the conventional Dirac bracket notation is used, $\Psi = |\Psi\rangle$ and $|\Psi\rangle\langle\Psi|$ is the projection operator on the state Ψ). However, this deterministic evolution is broken by any measurement – intervention of the observer with a classical gauge instrument. The measurement is not reversible and apparently not unitary evolution. The von Neumann projection produces entropy in an ideal manner – it is assumed as the ansatz that the entropy generated by the randomness of the quantum measurement is complete and equals 1 per bit. This is the reason that QRNGs are regarded as ideal perfect randomness sources. Realization of prefect von Neumann projection, is however, complicated in practice – thus the related QRNGs are rather slow and ineffective for expected applications.

B.4 Quantum transitions – the source of entropy for QRNGs

The time-dependent perturbation calculus describes the pattern of transition between the stationary states of a quantum system under the influence of any time-dependent perturbation:

- $\hat{H}_0 \phi_n = E_n \phi_n$ – stationary states (eigenstates of Hamiltonian) of unperturbed system,
- perturbed Hamiltonian

$$\hat{H}(t) = \begin{cases} \hat{H}_0, & \text{for } t < 0, t > T \\ \hat{H}_0 + \hat{V}(t), & \text{for } 0 < t < T. \end{cases}$$

The non-stationary state during the operation of the disturbance can be decomposed in the base of the stationary states of the undisturbed system. Then, using the Schrödinger equation, it is easy to find the formula for the probability of transition from the n stationary state to k stationary state under the influence of $\hat{V}(t)$ disturbance in the first order with respect to the disturbance[40],

$$p_{kn} = \frac{1}{\hbar^2} \left| \int_0^T e^{i\omega_{kn}t} V_{kn}(t)\, dt \right|^2, \tag{23}$$

where $\omega_{kn} = \frac{E_k - E_n}{\hbar}$, V_{kn} is a matrix element of the perturbation between stationary states n-initial and k-final.

For simple perturbation,

$$\hat{V}(t) = \begin{cases} 0, & \text{for } t < 0 \\ V, & 0 < t < T \end{cases} \tag{24}$$

one finds,

$$p_{kn} = |V_{kn}|^2 \frac{sin^2(\omega_{kn}T/2)}{(\hbar\omega_{kn}/2)^2}. \tag{25}$$

From the above we see that the maximum of p_{kn} is proportional to time square, T^2 where T is the time duration of the perturbation.

B.5 Fermi golden rule

Taking into account that (in the formula (25))

$$\lim_{T \to \infty} \frac{\sin^2\left(\frac{x}{2}T\right)}{\left(\frac{x}{2}\right)^2 T} \approx 2\pi\delta(x) \tag{26}$$

and assuming that the final states n belong to the continuous spectrum (similar as for a classical system) one can remove the Dirac delta via integration over final states. In the result the Fermi golden rule is obtained,

$$\frac{p_{kn}}{T} = \int \frac{2\pi}{\hbar} |V_{kn}|^2 \delta(E_k - E_n) \rho(E_k)\, dE_k, \tag{27}$$

($\rho(E_k)$ – density of final states) and in more general case of periodic perturbation[40],

$$\frac{p_{kn}}{T} = \int \frac{2\pi}{\hbar} |V_{kn}|^2 \delta(E_k - E_n + \hbar\omega) \rho(E_k)\, dE_k. \tag{28}$$

The essential feature of both of the above results is the probability of transition under the influence of an engaged perturbation – this probability is expressed per unit of time (and constant, in contrast to (23), upon the Fermi golden rule

probability of transition caused by a perturbation is again proportional to time T). Nevertheless, the transition is still quantum, i.e., it corresponds to a truly random behavior. Time sampling of this random process (taking into account the stability of the probability of the transition per unit of time) produces a truly random sequence – it is the perfect randomness. Thus, the Fermi golden rule and the quantum transitions carried out according to it can be used to construct a wide class of QRNGs. Such QRNG operating acc. to the Fermi golden rule are quick and suitable for information applications.

All phenomena of microscopic transport (especially in semiconductor devices like diodes or transistors) belong to quantum transition driven effects, thus are perfect sources of entropy, which can be applied to construct QRNGs. Similarly, the photo-effect effect, which occurs in photovoltaic cells of various types including plasmonic metallic structures and other optically driven microscopic phenomena especially in opto-electronic devices are governed by transitions of Fermi golden rule type, thus assigned by perfect entropy gain. According to the second law of thermodynamics the increase of the entropy is associated with irreversible transitions and the source of this entropy increase is quantum nonunitary evolution, which undergoes in all open microscopic systems with contact with a large reservoir (environment). The arising quantum entanglement of an open quantum system with the surroundings leads to mixed states shared by a subsystem and the environment, which as the whole evolves unitary according to the common pure though entangled quantum state. the subsystem in such a case is not in a pure quantum state but in a mixed state described by the density matrix operator $\hat{\rho} = \sum_n p_n |\Psi_n\rangle \langle\Psi_n|$, where $|\Psi_n\rangle$ are elements from the ortho-normal basis in the Hilbert space for this subsystem, and p_n is the probability distribution. The entropy $S = -Tr(\hat{\rho} ln(\hat{\rho})) \rightarrow -\sum_n p_n ln(p_n)$ and attains maximum for uniform probability distribution of p_n. We see that the source of entropy is related with perturbation of a system by the surroundings, which has irreversible character.

C Commercial QRNGs overview

C.1 IDQ Quantis Random Number Generator

Generator by the Swiss company idQuantique, which is a pioneer in the commercialization of quantum cryptography (Clavis series) and quantum random generators (Quantis). The company was founded as a spin-off of the University of Geneva. The QRNGs were initially components of the cryptographic Clavis quantum key distribution sets, only after some time they were introduced to the offer as separate devices.

Speed: PCIe 4/16 Mb/s, USB 4 Mb/s

source of randomness: the light source illuminates the semi-transmissive mirror and hits the two detectors

Advantages described by the manufacturer:

- True Quantum Randomness
- Most certified generator [NIST SP800-22 Test Suite Compliance; METAS Certification; CTL Certification; Several iTech Labs individual Certificates; Compliance with the BSI's AIS31 standard [Quantis dedicated version]]
- Thermal noise contribution $< 1\%$
- Storage Temperature -25°C to 85°C
- High speed up to 16 Mbps
- Possibility to extract randomness
- Continuous status check
- Low cost
- Compact and Reliable
- easy integration
- Instant entropy
- USB external device/PCIe card

Doubts: doubtful quantum – the source is not single-photon, detectors are not single-photon

C.2 ComScire PureQuantum

Generator of the American company ComScire specializing in creating generators based on physical phenomena. The company was established for this purpose in 1994. Its creator is the inventor Mr. Wilber, known e.g., from the invention of the heart rate monitor (one of the most valuable medical inventions of the 20th century). The company guarantees that all components are made in the United States. He considers his product to be the most technologically advanced and reliable (in the sense of long-term operation) commercially available product.
Speed: USB 4/32/128 Mb/s
Entropy source: current noise associated with leakage of carriers due to tunneling in MOS transistors
Advantages described by the manufacturer:

- distortion 0/1 and autocorrection < 1 per 10^{18}
- Estimated Quantum Entropy: 0.999+ (4 Mb/s), 0.87 (128 Mb/s) bit per output bit
- Estimated Total Entropy: $(1-e)$ bits per bit, $e < 10^{-100}$
- Permissible ambient temperature 0°C to 50°C
- Humidity without condensation
- Designed To Meet NIST SP 800-90 B Recommendations
- The manufacturer guarantees that the device will pass every random test
- Continuous hardware verification with automatic halt
- Access to raw data stream and internal statistics
- Independent power regulation for generator system
- USB external device

Doubts: it is difficult to precisely determine whether the noise is a purely quantum result or whether the classical component predominates there.

C.3 Toshiba UFICS-QRNG

Generator of the Japanese Toshiba Corporation (the Europe branch, active i.a. in the QKD field), developed within the Horizon2020 project, under the code nam UFICS-QRNG (Ultra-Fast, Integrated, Certified Secure Quantum Random Number Generator)[2].
Speed: 8 Gb/s (SATA)
Entropy Source: phase noise from spontaneous emission from a pulsed laser
Advantages described by the manufacturer:

- generates numbers at ultrafast rate (8 Gb/s)
- a self-contained and compact unit 10 x 23 x 5 cm3
- provided with embedded monitor functions that allows to run the generator diagnostics in real time
- real time post-processing
- communication protocols USB/100M, 10G/SATA
- passing of test suites Test-U01 (including Big Crush battery) and NIST SP-800-22

Doubts: the quantum nature of the generation process is unclear, due to the operation of multi-photon and not single-photon bundles.

C.4 PicoQuant PQRNG 150

Generator by the German company PicoQuant, founded in 1996, specializing in the production of specialized optical and electronic devices and components (pulsed diode lasers, time-tagged data aggregation devices, single-photon detectors, etc.). Its offer includes a generator that uses a high-speed quantum process. The manufacturer does not publish the detailed parameters of the device.
Speed: 150 Mbps USB
Entropy Source: generation process based on photon arrival times
Advantages described by the manufacturer:

- Precision of 1ps photon arrival times
- Fast generation 150 Mb/s
- Determination of photon arrival times supported by hardware postprocessing
- The device passes all major randomness tests
- USB external device

Doubts: the quantum nature of the generation process is unclear, due to the operation of multi-photon and not single-photon bundles.

C.5 Whitewood Entropy Engine

Generator from the American company Whitewood, established to commercialize its own quantum random number generator for cryptographic applications, along with supporting IT solutions. Solutions are directed to large data centers or clouds, where high generation speed is needed (devices can be connected together to form generator matrices).
Speed: 350 Mbps PCI Express
Entropy source: lensing a photon beam onto the detector
Advantages described by the manufacturer:

- Entropy level > 99.4% (speed 350 Mb/s; at 200 Mb/s hash SHA512 100% entropy)
- Operating temperature: 5°C to 65°C (storage -25°C to 80°C)
- Humidity allowed: 0 to 80 % RH at 40°C
- Test Compatibility: NIST SP 800-22, NIST SP 800-90B, Alphabit, Dieharder, FIPS 140, TEST U01
- Raw Stream Access
- Real-time testing of the generation process (continuous repeat testing, continuous adaptation testing, full component test on power-up)
- PCIe card

Doubts: the quantum nature of the generation process is unclear, due to the operation of multi-photon and not single-photon bundles.

C.6 QuintessenceLabs qStream

Generator of the Australian company QuintessenceLabs established as a spin-off of the Australian National University. The company offers comprehensive solutions in the field of modern cryptography, including quantum cryptography. The proposed solutions, both the quantum key distribution (QKD) and QRNG systems, are characterized by very high speed parameters (related to the physical model used, which, however, on the other hand, may be questioned as not fully quantum).
Speed: 1 Gbits/s RJ-45
Entropy source: dividing a multi-photon laser beam into two and "subtracting" them from each other.
Advantages described by the manufacturer:

- Generation 1 Gbits/s of conditioned homogeneous entropy / 8 Gbits/s of unqualified entropy
- Central key management system or direct random sequence via TCP
- Generated sequences can be immediately distributed among multiple devices

- Characterization of random sequences (owner, purpose of use, current lifetime, access history, etc.)
- Conforms to NIST SP800-90 Requirements, Non-NIST Conditioning Support
- Access to the raw generated data stream
- Rackmount Unit 19â€™

Doubts: the quantum nature of the generation process is unclear, due to the operation of multi-photon and not single-photon bundles.

C.7 QuantumNumbersCorp QNG2

Generator by the Canadian company Quantum Numbers Corporation (QNC) established for the commercialization of security systems based on QRNG. The company's activities are aimed at creating microprocessor solutions for the broad financial, banking, military, mobile devices and telecommunications markets. They propose the first QRNG chip in the form of a chip on the commercial market.
Speed: 1 Gbits/s
Entropy Source: quantum tunneling
Advantages described by the manufacturer:

- Minimum QRNG chip integrated on chip – dimensions 20 x 20 microns
- The circuits can be paralleled with high speeds (one chip even up to 1G bits/s)
- Low power consumption, typically < 1 mW
- Operating temperature -50°C to 85°C
- Easy integration with existing electronic devices
- Possibility of integration in mobile devices
- Cost effectiveness compared to other QRNG devices
- Randomness confirmed by NIST and Diehard tests

Doubts: the quantum nature of the generation process is unclear – electron transport processes may have a significant classical component.

C.8 EYL Micro Quantum Random Number Generator

The generator of the South Korean company EYL (Everywhere in Your Life, founded in 2015), which promotes its technology based on the decay of radioactive isotopes, miniaturized to the size of a chip. The company was awarded the title of the most promising startup in 2016 in the international competition in Boston, USA (out of 5500 applicants). The offer is focused on the integration of QRNG with all possible IT-based solutions, in particular with intelligent factories, smart homes or intelligent mobility (e.g., intelligent cars).
Speed: 40 Gb/s
Entropy source: decay of radioactive isotopes
Advantages described by the manufacturer:

- Second generation chips – current 2mm x 2mm
- Thermal noise contribution $< 0.1\%$
- USB peripheral, PCB module, PCIe card, all-in-one server device
- USB 7 Kbits/s 1 Mbits/s or 1.6 Gbits/s $\pm 10\%$
- PCIe 40 Gbits/s $pm10\%$
- Dedicated server, speed 7 kbits/s 43 Gbits/s
- Operating temperatures -45°C to 85°C (USB), 0°C to 60°C (PCIe)
- Full compliance with NIST SP800-22, AIS.31, Diehard, and NIST SP800-90B recommendations

Doubts: the method of measuring the radiation is not precisely defined at the given speeds, most likely an important classical component.

C.9 qutools quRNG

Generator of the German company qutools, which is a spin-off of the University in Munich from 2005. The company specializes in the production and distribution of quantum computing devices, including sources of entangled photons, QRNGs, quantum cryptography systems and optics in the quantum regime.
Speed: 50 Mb/s USB
Entropy source: uncertainty of detection times with single-photon detectors of photons emitted from the LED.
Advantages described by the manufacturer:

- If the emitted light from the LED is properly tuned, it is not necessary to post process the generated signal
- The layout performs continuous tests of the generated sequence
- Compatible with tests such as NIST, Dieharder
- USB external device
- Easy to use device
- Source of randomness physically characterized

Doubts: quality of single-photon detectors

C.10 Micro Photon Devices Quantum Random Number Generator

Generator of the Italian company Micro Photon Devices, which is a spin-off of the Polytechnic University of Milan, founded in 2004. The company manufactures specialized optical equipment in the quantum regime (e.g., single-photon SPAD detectors), including QRNG based on the phenomena of quantum optics.
Speed: 128 Mb/s USB
Entropy Source: internal characteristics of the illuminated quantum detector in the form of an avalanche diode.
Advantages described by the manufacturer:

- Generated is treated as ready to use, with no additional cleanup processes
- Speeds 16/32/64/128 Mb/s
- USB external device
- Set of statistical tests, including NIST, Dieharder, TestU01
- Easy modification of software (libraries C, C++, C#, Matlab)
- Input initializing with sync
- Conforms to EU RoHS 2 Directive

Doubts: ensuring that there is no need to verify the randomness of the generated sequence; quality of single-photon detectors

C.11 QuantumCTek Quantum Random Number Generator QRNG100E

Generator of the Chinese company QuantumCTek Co., Ltd., a Chinese pioneer and leader in commercialized quantum information technology. The product is claimed to be one of the fastest commercial quantum random number generator(QRNG) around the world.
Speed: 600 Mb/s USB
Entropy Source: based on the phase noise of laser spontaneous emission.
Advantages described by the manufacturer:

- Speeds 200/600 Mb/s
- USB or network external device
- Conform to the specification of the GM/T 0005-2012 and NIST SP 800-22
- Support real-time monitoring and control system status
- Support secondary development and multi platform operating system

Doubts: lack of information about passing randomness tests; quantumness of the process

C.12 Quside FMC 400

Generator of the Spanish company Quside, which is a spin-off of The Institute of Photonic Sciences (ICFO) in Barcelona, founded in 2017. In 2020 the company launched their first QRNG Quside FMC 400, based on Quside's proprietary phase-diffusion quantum random number generation technology.
Speed: 400 Mb/s USB
Entropy Source: phase-diffusion process for light emitter due to spontaneous emission events.
Advantages described by the manufacturer:

- Designed specifically for high-performance FPGA-based systems
- Speeds 400 Mb/s
- External device with USB, PCIe and Ethernet interface
- Objective quality prove is possible thanks to Quside Randomness Metrology toolkit
- With entropy bounds above 90% reliable entropy quality is guaranteed

Doubts: lack of information about passing randomness tests; own testing suite; quantumness of the process

C.13 Commercial QRNGs Summary

The Table 1 summarizes the essential features of the commercially available quantum random number generators presented above. It is worth emphasizing that regardless of the quality of the quantum nature of a given generation process, the testability of, among others, NIST battery testing is the primary advantage presented by the manufacturers. Some important aspects have been omitted from the table, e.g., the possibility of being miniaturized to the chip size. Doubts about the nature of the source of entropy in given solutions were also presented. None of the currently available commercial generators is based on quantum entanglement, which is most likely caused by low generation speed parameters, implementation difficulties or costs of such solutions – but on the other hand, no doubt the fundamentally quantum nature would outweigh these disadvantages.

Table 1. Features of commercial quantum random number generators

qrng	generation speed Mb/s	link	source entropy	self-test.	compatibility	bit access	doubts
Quantis	4, 16	USB, PCIe	quantum optics.	yes	NIST, METAS, CTL, BSI's AIS31	no	quantum simulation
ComScire	4, 32, 128	USB	electr.	yes	all	no	quantum simulation
Toshiba	8000	USB, SATA	quantum optics.	no	TestU01, NIST	no	quality of single-photon detectors
PQRNG150	150	USB	quantum optics.	no	confirmed for selected	no	quality of single-photon detectors
Entropy Engine	350	PCIe	quantum optics.	no	NIST, Alphabit, Dieharder, FIPS140, TestU01	no	quality of single-photon detectors
qStream	1000	Ethernet	quantum optics.	yes	NIST and chosen	yes	quantum simulation
QNG2	1000	chip	tunnel. quantum.	no	NIST, Dieharder	no	quantum simulation
MQRNG	40000	USB, PCIe, PCM	radioactic decay.	no	NIST, AIS.32, Diehard	no	measurement method
quRNG	50	USB	quantum optics.	yes	NIST, Dieharder	no	quality of single-photon detectors
MPD QNRG	16, 32, 64, 128	USB	quantum optics.	no	NIST, Dieharder, TestU01	yes	quality of single-photon detectors
QRNG100E	200, 600	USB, Ethernet	quantum optics.	no	GM/T 0005-2012 and NIST	yes	quantumness of the process
Quside FMC 400	400	USB, PCIe, Ethernet	quantum optics.	yes	Quside Randomness Metrology	yes	quantumness of the process

D Popular test batteries or test suits for the statistical randomness verification

D.1 NIST Test

The National Institute of Standards and Technology of the United States developed a 2010 set of statistical tests for random and pseudorandom generators for cryptographic applications. The set consists of 15 tests verifying the deviation of statistical parameters characterizing the tested sequence from the parameters determined statistically for a perfectly random sequence.

The kit includes,

1. Test for the frequency of single 0 and 1 occurrences in the studied sequence

2. Test for the frequency of single 0 and 1 occurrences in the block of the studied sequence
3. Mileage test
4. Test of the longest run in the block of the studied sequence
5. Binary matrix order test – test of linear relationships between subsequences of the same length of the tested sequence
6. Spectral test – Discrete Fourier transform test
7. Test for occurrence of non-overlapping patterns
8. Test for overlapping patterns
9. Universal statistical Maurer test
10. Linear complexity test
11. Serial test – pattern distribution test
12. Entropy estimation test
13. Cumulative sum test
14. Random trip test
15. Variant random trip test

The NIST tests partially overlap with, for example, the Diehard (Dieharder) tests listed below.

D.2 Test U01

Test U01 is a C language library for empirical testing of random number generators created by a team at the University of Montreal in 2007. The following tests belong to the library:

1. Module of tests of the distribution of t-dimensional vectors in a unit hypercube – 4 tests
2. Module of tests based on discrete and continuous empirical entropies – 5 tests
3. Test module based on the distance between the nearest points in a sample of n equally distributed points on a unit torus in t-dimensional space – 3 tests
4. Classic module of tests described by Knuth in the book[3] – 11 tests
5. Diehard/Dieharder test module described further – 9 tests
6. Statistical primitive-based test module – 8 tests
7. Test module based on discrete random walks over the set of all integers – 4 tests
8. Test module based on the change of linear sequence complexity with its increase and the compressibility test – 2 tests
9. Test module using spectral methods (mainly discrete Fourier transform)

D.3 Diehard and Diehard er tests

Diehard is a set of randomness tests created by the American mathematician and computer scientist George Marsaglia in 1996[4] and revised (in modularization, implementation, and functionality in general) in 2006 by Robert G. Brown, who named the modified Dieharder kit[5].

The following tests are included in the kit,

1. Birthday spacing test – a statistical test of the number of duplicate intervals between successive properly defined birthdays (based on the test sequence).
2. Overlapping 5-permutation test – determination of the frequency of occurrence of states from 120 possible for 5 integers (5! permutations of certain numbers) =.

3. Binary rank test 31x31 – binary order test for 31x31 matrices.
4. Binary rank test 32x32 – binary order test for 32x32 matrices.
5. Binary rank test 6x8 – binary order test for 6x8 matrix.
6. Bitstream test – search for missing 20 letter (letters 0 and 1) words in overlapping 20 letter words into which the analyzed sequence is divided.
7. OPSO, OQSO and DNA tests,
   - OPSO – test for the rare occurrence of overlapping pairs (2 letter words composed of a 1024 letter alphabet).
   - OQSO – test for the rare occurrence of overlapping fours (4 letter words composed of a 32 letter alphabet).
   - DNA – a test based on two-bit sequences encoding the letters C, G, A, T forming 10 letter words.
8. Count-the-1's test stream of bytes – test to count ones in the byte stream.
9. Count-the-1's test stream of specific bytes – test of counting ones in a stream of selected bytes.
10. Parking lot test – test consisting in random 'parking' in a 'parking lot' of 100 out of 100 circles with a radius of 1 without collision (i.e., without overlapping the circles).
11. Minimum distance test – test of the minimum distance among 8000 points selected in a square with side 10000.
12. 3D-Sphere test TE – random selection of 4000 points in a cube of side 1000 – at each point a sphere is created with the size reaching the next occupied point. Sphere sizes are to be distributed appropriately.
13. Squeeze test – random integers are converted into fractions from the interval [0,1] – the number of iterations of reducing the number to 1 by rounding is tested.
14. Overlapping sums test – test of overlapping sums.
15. Runs test – test of the number of runs up or down in fractional integer transformations.
16. Craps test – play 200,000 dice games, specifying the number of wins and the number of throws to end the game.

E Detailed description of the NIST battery tests

E.1 Test of the frequency of single 0 and 1 occurrence in the studied sequence

Adapted from[6–8].

A truly random sequence of 0 and 1 should be balanced. This corresponds to the schedule Bernoulli with a probability of $\frac{1}{2}$ getting 1 or 0. The de Moivre-Laplace theorem states that for a sufficiently large number of samples the sum of the Bernoulli random variable normalized to the number of trials $\sqrt{n}$ can be approximated by the normal distribution. So, one could write[9],

$$\begin{aligned} &\lim_{n\to\infty} P\left(\frac{\sum_{i=1}^{n}(2\varepsilon_i - 1)}{\sqrt{n}} \leq z\right) = \Phi(z) \\ &= \frac{1}{\sqrt{2\pi}} \int_{-\infty}^{z} e^{-\frac{u^2}{2}} du, \end{aligned} \tag{29}$$

where $\Phi(z)$ is the standard normal distribution,

$$\begin{aligned} &\Phi(z) = \frac{1}{\sqrt{2\pi}} \int_{-\infty}^{z} e^{-\frac{u^2}{2}} du = \frac{1}{\sqrt{\pi}} \int_{-\infty}^{\frac{z}{\sqrt{2}}} e^{-u^2} du \\ &= \frac{1}{\sqrt{\pi}} \int_{0}^{\frac{z}{\sqrt{2}}} e^{-u^2} du + \frac{1}{\sqrt{\pi}} \int_{-\infty}^{0} e^{-u^2} du \\ &= \frac{1}{2}\left(\frac{2}{\sqrt{\pi}} \int_{0}^{\frac{z}{\sqrt{2}}} e^{-u^2} du + \frac{2}{\sqrt{\pi}} \int_{-\infty}^{0} e^{-u^2} du\right) \\ &= \frac{1}{2}\left(\operatorname{erf}\left(\frac{z}{\sqrt{2}}\right) + 1\right), \end{aligned} \tag{30}$$

where the error function is $\mathrm{erf}(z) = \frac{2}{\sqrt{\pi}} \int_0^z e^{-u^2} du$. In this test, a positive z is assumed as it does not make a difference whether the number of ones (zeros) exceeds the number of zeros (ones) non-randomly. So one can write,

$$
\begin{aligned}
&\Phi(z) = 1 - \Phi(-z), \\
&\Phi(-z,z) = \Phi(z) - \Phi(-z) = \Phi(z) - (1 - \Phi(z)) \\
&= 2\Phi(z) - 1, \\
&\Phi(-z,z)^c = 1 - \Phi(-z,z) = 2(1 - \Phi(z)), \\
&2(1 - \Phi(z)) = 2\left(1 - \frac{1}{2}\left(\mathrm{erf}\left(\frac{z}{\sqrt{2}}\right) + 1\right)\right) \\
&= 1 - \mathrm{erf}\left(\frac{z}{\sqrt{2}}\right) = \mathrm{erfc}\left(\frac{z}{\sqrt{2}}\right).
\end{aligned}
\tag{31}
$$

Hence for the statistic $S_{\mathrm{obs}} = \frac{|S_n|}{\sqrt{n}}, S_n = \sum_{i=1}^{n} (2\varepsilon_i - 1)$, P takes the form

$$
P = \mathrm{erfc}\left(\frac{S_{\mathrm{obs}}}{\sqrt{2}}\right),
\tag{32}
$$

where the accompanying error function is expressed as $\mathrm{erfc}(z) = \frac{2}{\sqrt{\pi}} \int_z^\infty e^{u^2} du$.

One can perform the test in the following way,

1. Convert all zeros in the sequence to be tested to -1, then add all the values, $S_n = \sum_{i=1}^{n} (2\varepsilon_i - 1)$, where ε_i is the value of i-th position in the sequence.
2. Calculate stat for $S_{\mathrm{obs}} = \frac{|S_n|}{\sqrt{n}}$.
3. Calculate the value of P using the accompanying error function, $P = \mathrm{erfc}\left(\frac{S_{\mathrm{obs}}}{\sqrt{2}}\right)$.
4. If the value of P is greater than the adopted significance level (typically selected between values between 0.001 and 0.01), then the sequence can be considered random.

This test is considered fundamental as many other tests are based on its result. It is worth noting that this test will give a positive result for the sequence, the first half of which consists of all ones and the second one of all zeros – of course, such a sequence is not random – hence a test that analyzes the appropriate distribution of zeros and ones along the entire length of the sequence is necessary.

If the resulting value of P is less than the acceptable threshold (0.01) then the sequence should be considered non-random. It is advisable that the tested sequence consists of a minimum of 100 bits.

E.2 Test of the frequency of single 0 and 1 occurrences in the block of the tested sequence

Adapted from[3,6,10,11].

This test analyzes the proportion of the occurrences of zeros and ones in each N block of bits of length M, where $MN = n$ is the number of bits in the tested sequence. The test checks whether deviations from the ideal proportion, $\frac{1}{2}$, for the ones (zeros) in each block, can be considered random or not. If $M = 1$, this test is the same as the previous test (Test of the frequency of single 0 and 1 occurrences in the sequence under study).

The χ^2 test is applied to the initial sequence divided into N of non-overlapping blocks of M length by comparing the calculated proportion of ones in the sub-sequence to $\frac{1}{2}$:

$$
\chi^2_{\mathrm{obs}} = 4M \sum_{i=1}^{N} \left(\pi_i - \frac{1}{2}\right)^2,
\tag{33}
$$

where $\pi_i = \left(\sum_{k=1}^{M} \varepsilon_k^{(i)}\right) / M$ and $\varepsilon_k^{(i)}$ is the value of k-th bit in i-th block.

The value of P is then computed using the incomplete gamma function for $Q(a,x)$ defined as,

$$
Q(a,x) = 1 - P(a,x) = \frac{\Gamma(a,x)}{\Gamma(a)} = \frac{1}{\Gamma(a)} \int_x^\infty e^{-t} t^{a-1} dt,
\tag{34}
$$

where $Q(a,0)=1$, $Q(a,\infty)=0$ and $\Gamma(x)=\int_0^\infty t^{x-1}e^{-t}dt$.

The value of P takes the form,

$$\frac{\int_{\chi^2_{\text{obs}}}^{\infty} e^{-\frac{u}{2}} u^{\frac{N}{2}-1} du}{\Gamma\left(\frac{N}{2}\right) 2^{\frac{N}{2}}} = \frac{\int_{\frac{\chi^2_{\text{obs}}}{2}}^{\infty} e^{-u} u^{\frac{N}{2}-1} du}{\Gamma\left(\frac{N}{2}\right)}, \tag{35}$$

what can be written as so-called *complementary regularized upper incomplete gamma function*, $\Gamma_C(s,x)=\frac{1}{\Gamma(s)}\int_x^\infty t^{s-1}e^t dt$.

If the resulting value of P is less than the acceptable threshold (0.01) then the sequence should be considered non-random.

It is advisable that the tested sequence consists of a minimum of 100 bits. The length of the M block should be chosen such that $M \geq 20, M > 0.01n$ and $N < 100$.

E.3 Runs test

Adapted from[6, 12, 13].

The length of k is defined as a sequence of consecutive identical bits of length k. Such a sequence is flanked before its beginning and end by bits of the opposite value. The Waveform Test analyzes the total number of passes in a sequence to determine whether the number of 0 and 1 runs of different lengths remains similar to the number of such runs for a true random sequence. This test can tell you whether the jumps between 0s and 1s happen too often or too rarely.

In order to calculate the number of runs, we define the function $r(k)$,

$$r(k) = \begin{cases} 0, & \text{for} \quad \varepsilon_k = \varepsilon_{k+1}, \\ 1, & \text{for} \quad \varepsilon_k \neq \varepsilon_{k+1}, \end{cases} \quad k = 1, \ldots, n-1. \tag{36}$$

Total number of runs in n-bit sequence, $\varepsilon = \varepsilon_1 \varepsilon_2 \ldots \varepsilon_n$, defined as V_n, may be computed as follows,

$$V_n = \sum_{k=1}^{n-1} r(k) + 1. \tag{37}$$

It is assumed that the distribution of the total number of passes V_n converges to normal as n increases, for a fixed proportion of $\pi = \sum_j \frac{\varepsilon_j}{n}$ (which is after checking near frequency test $\frac{1}{2}$, $\left|\pi - \frac{1}{2}\right| \leq \frac{2}{\sqrt{n}}$, otherwise the value of P is 0),

$$\lim_{n\to\infty} P\left(\frac{V_N - 2n\pi(1-\pi)}{2\sqrt{n}\pi(1-\pi)} \leq z\right) = \Phi(z). \tag{38}$$

Thus, the value of P is computed as the accompanying error function,

$$P = \operatorname{erfc}\left(\frac{|V_n(\text{obs}) - 2n\pi(1-\pi)|}{2\sqrt{2n}\pi(1-\pi)}\right). \tag{39}$$

If the resulting value of P is less than the acceptable threshold (0.01) then the sequence should be considered non-random.

It is advisable that the tested sequence consists of a minimum of 100 bits.

E.4 Longest run of ones in the block test

Adapted from[6, 13–15].

Another factor characterizing the randomness of the sequence under study is the longest sequence consisting of consecutive ones. This test works on N blocks of M length, where NM is the length of the bit sequence under test. Depending on the total length, n, of the analyzed sequence, the value of M should be appropriately selected, for example: $n = 128 \to M = 8$, $n = 6272 \to M = 128$, $n = 750{,}000 \to M = 10^4$. Depending on the selected M, other parameters can be selected as follows: $M = 8 \to K = 3, N = 16$, $M = 128 \to K = 5, N = 49$, $M = 10^4 \to K = 6, N = 75$. $K+1$ specifies the number of 1 run classes as in Table 2. For each of these classes it is necessary to calculate how many runs (length as in Table 2) are in the analyzed block of length M.

In order to obtain the empirical frequencies needed to determine the value of P, the conditional probability for the longest run of 1s ν should be calculated as follows:

$$P(\nu \leq m|r) = \frac{1}{\binom{M}{r}} \sum_{j=0}^{U} (-1)^j \binom{M-r+1}{j} \binom{M-j(m+1)}{M-r}, \tag{40}$$

Table 2. Classes and probabilities for various real values of K and M

K	M	Class	Robability
3	8	$\{v \leq 1\}$	$\pi_0 = 0.2148$
3	8	$\{v = 2\}$	$\pi_1 = 0.3672$
3	8	$\{v = 3\}$	$\pi_2 = 0.2305$
3	8	$\{v \geq 4\}$	$\pi_3 = 0.1875$

K	M	Calss	Probability
5	128	$\{v \leq 4\}$	$\pi_0 = 0.1174$
5	128	$\{v = 5\}$	$\pi_1 = 0.2430$
5	128	$\{v = 6\}$	$\pi_2 = 0.2493$
5	128	$\{v = 7\}$	$\pi_3 = 0.1752$
5	128	$\{v = 8\}$	$\pi_4 = 0.1027$
5	128	$\{v \geq 9\}$	$\pi_5 = 0.1124$

K	M	Class	Probability
5	512	$\{v \leq 6\}$	$\pi_0 = 0.1170$
5	512	$\{v = 7\}$	$\pi_1 = 0.2460$
5	512	$\{v = 8\}$	$\pi_2 = 0.2523$
5	512	$\{v = 9\}$	$\pi_3 = 0.1755$
5	512	$\{v = 10\}$	$\pi_4 = 0.1027$
5	512	$\{v \geq 11\}$	$\pi_5 = 0.1124$

K	M	Class	Probability
5	1000	$\{v \leq 7\}$	$\pi_0 = 0.1307$
5	1000	$\{v = 8\}$	$\pi_1 = 0.2437$
5	1000	$\{v = 9\}$	$\pi_2 = 0.2452$
5	1000	$\{v = 10\}$	$\pi_3 = 0.1714$
5	1000	$\{v = 11\}$	$\pi_4 = 0.1002$
5	1000	$\{v \geq 12\}$	$\pi_5 = 0.1088$

K	M	Class	Probability
6	10000	$\{v \leq 10\}$	$\pi_0 = 0.0882$
6	10000	$\{v = 11\}$	$\pi_1 = 0.2092$
6	10000	$\{v = 12\}$	$\pi_2 = 0.2483$
6	10000	$\{v = 13\}$	$\pi_3 = 0.1933$
6	10000	$\{v = 14\}$	$\pi_4 = 0.1208$
6	10000	$\{v \geq 15\}$	$\pi_5 = 0.0675$
6	10000	$\{v \geq 16\}$	$\pi_5 = 0.0727$

where r is the number of 1s, $M-r$ the number of 0s in the m-bit block, $U = \min\left(M-r+1, \left[\frac{r}{m+1}\right]\right)$. Therefore,

$$P(v \leq m) = \sum_{r=0}^{M} \binom{M}{r} P(v \leq m|r) \frac{1}{2^M}. \tag{41}$$

Probabilities $\pi_0, \pi_1, \ldots, \pi_K$ (shown in Table 2), and empirical frequencies, $v_0, v_1, \ldots, v_K$ make the following test pattern χ^2,

$$\chi^2 = \sum_{i=0}^{K} \frac{(v_i - N\pi_i)^2}{N\pi_i}, \tag{42}$$

which for a random sequence has an approximated distribution of ϕ^2 with K degrees of freedom.

The analyzed value of P has the form,

$$\frac{\int_{\chi^2_{\text{obs}}}^{\infty} e^{-\frac{u}{2}} u^{\frac{K}{2}-1} du}{\Gamma\left(\frac{K}{2}\right) 2^{\frac{K}{2}}}, \tag{43}$$

what is *complementary regularized upper incomplete gamma function*.

If the resulting value of P is less than the acceptable threshold (0.01), then the sequence should be considered non-random.

It is recommended that the tested sequence should consist of increasingly 128, 6272 or 750 000 bits.

E.5 Binary matrix rank test

Adapted from[4, 6, 16, 17].

This test is also part of the Diehard tests.

It is possible to verify the randomness by analyzing linear dependencies among the equal length of blocks (sequences) of the tested sequence. For this purpose, construct matrices using the sequence under study (by dividing the sequence into successive subsequences as rows and columns) and find linear correlations between rows and columns in matrices. In such matrices the deviation of an order (or lack thereof) from the theoretical values can be tested from the point of view of statistics.

In the case of random two-dimensional matrices with dimensions $M \times Q$, their orders can take the value $r = 0, 1, 2, \ldots, m$, where $m = \min M, Q$, with probabilities defined as,

$$p_r = 2^{r(Q+M-r)-MQ} \prod_{i=0}^{r-1} \frac{\left(1-2^{i-Q}\right)\left(1-2^{i-M}\right)}{1-2^{i-r}}. \tag{44}$$

The values of M and Q may be equal, then M will be the only parameter in the test. In the case when $n = M^2N$ then N will be the new sample size, and when $n \neq M^2N$ then M and N are selected such that $n - M^2N$ will be negligibly small.

In practice, $N = \left[\frac{n}{MQ}\right]$ disregarding a small number of bits in each sub-sequence.

May be assumed, based on the probabilities in the case of the theoretical random sequence for $M = Q = 32$, $p_M \approx \prod_{j=1}^{\infty}\left[1-\frac{1}{2^j}\right] = 0.2888\ldots$, $p_{M-1} \approx 2p_M \approx 0.5776\ldots$, $p_{M-2} \approx \frac{4}{9}p_M \approx 0.1284\ldots$ that all other probabilities for $M \geq 10$ are relatively small, ≤ 0.005.

After calculating the orders, frequency F_M – number of matrices of order equal to M, F_{M-1} – number of matrices equal to $M-1$ and $N-F_M-F_{M-1}$ – number of matrices of order not exceeding $M-2$ are calculated.

The statistics are defined as follows:

$$\chi^2 = \frac{(F_M-p_MN)^2}{p_MN} + \frac{(F_{M-1}-p_{M-1}N)^2}{p_{M-1}N} + \frac{(N-F_M-F_{M-1}-(1-p_M-p_{M-1})N)^2}{(1-p_M-p_{M-1})N}, \tag{45}$$

which, assuming the randomness of the sequence under study, should have an approximate distribution of ϕ^2 with two degrees of freedom.

The analyzed value of P is:

$$P = e^{-\frac{\chi^2_{\text{obs}}}{2}}. \tag{46}$$

If the value of χ^2_{obs} is large, the deviation from the distribution of the random sequence is significant.

If the resulting value of P is less than the acceptable threshold (0.01), then the sequence should be considered non-random.

It is advisable to choose n so that when $M = Q = 32$, $n \geq 38MQ$, then $n = 38912$.

E.6 Spectral test – Discrete Fourier transform test

Adapted from[6, 18–20].

This test is one of the spectral methods based on the Discrete Fourier Transform. The test looks for cyclical behavior in the analyzed bit sequence that could testify to a lack of randomness.

In the sequence under test, bits are encoded as -1 and $+1$. The discrete Fourier transform takes the form:

$$f_j = \sum_{k=1}^{n} x_k e^{i2\pi\frac{(k-1)j}{n}}, \tag{47}$$

where x_k corresponds to the bit at position k in the initial sequence, $k = 1,\ldots,n$, $j = 0,\ldots,n$. As the Fourier transform is symmetric over the real and the imaginary parts, only values from 0 to $\frac{n}{2}-1$ are considered here. If the sequence x_k is random, the value of the Fourier transform modulus $|f_j|$ should be less than $h = \sqrt{\left(\log\frac{1}{0.05}\right)nin}$ 95 % of cases. The P value comes from the binomial distribution.

$$d = \frac{N_1 - N_0}{\sqrt{\frac{n(0.95)(0.05)}{4}}}, \tag{48}$$

where N_1 is the number of vertices smaller than h – only the first half of the vertices is considered ($\frac{n}{2}$ – due to symmetry real and imaginary parts of the transform). The value of P is defined as follows,

$$2\left(1-\phi\left(|d|\right)\right) = \operatorname{erfc}\left(\frac{|d|}{\sqrt{2}}\right), \tag{49}$$

where $\phi(x)$ is the normal distribution and erfc is the complementary error function.

Other values of P can be defined as well, using the Fourier transform or its modulus to analyze deviations from random behavior over the bits.

If the resulting value of P is less than the acceptable threshold (0.01) then the sequence should be considered non-random.

It is advisable that the sequence to be tested consists of a minimum of 1000 bits.

E.7 Test for occurrence of non-overlapping patterns

Based on[6, 21].

This test examines the occurrence of certain predefined patterns of a non-periodic nature in order to analyze whether there are too many of them in the test sequence. When parsing the input sequence, a m window is used, configured to detect a specific

pattern of m length. In case the pattern is not found, the window advances one bit and checks again. When a pattern is found, the window moves to the bit after the last bit in the pattern found in search of the next occurrence.

The pattern can be defined as,

$$B = \left(\varepsilon_1^0, \ldots, \varepsilon_m^0\right), \tag{50}$$

where m is the fixed length. Patterns are selected as parameters for this test. Table 3 and Table 4 contains examples of aperiodic patterns. The exponential growth of the number of patterns with the increase of the patterns length results in exponential growth of the testing time – this happens while checking the long-range correlations, which requires longer patterns.

The pattern B can be described by its set of B periods,

$$\mathrm{B} = \left\{ j, 1 \leq j \leq m-1, \varepsilon_{j+k}^0 = \varepsilon_k^0, k = 1, \ldots, m-j \right\}, \tag{51}$$

for example, for B corresponding to a sequence of all 1s of length m its set of periods $\mathrm{B} = \{1, \ldots, m-1\}$. For B aperiodic patterns, the set of B periods is empty. Such a pattern cannot be written in the form $ll\ldots ll'$ for l less than B with l' specifying the l prefix. In such cases, the occurrences of B in the analyzed sequence do not overlap.

In the considered test, the number of occurrences of a given pattern is statistically analyzed. It is defined as,

$$W = W(m,n) = \sum_{i=1}^{n-m+1} I\left(\varepsilon_{i+k-1} = \varepsilon_k^0, k = 1, ..., m\right), \tag{52}$$

where m is the length of the pattern B, ε_i is i-th bit of the analyzed sequence (lengths M) and ε_i^0 is the i-th bit of the given pattern. The W statistic is also defined for aperiodic patterns, where $\mathrm{B} = \emptyset$. In the case under consideration, according to the central limit theorem, random variables $I\left(\varepsilon_{i+k-1} = \varepsilon_k^0, k = 1, ..., m\right)$ are dependent on m. The parameters of the normal distribution, mean and variance, are defined as,

$$\mu = \frac{n-m+1}{2^m}, \quad \sigma^2 = n\left(\frac{1}{2^m} - \frac{2m-1}{2^{2m}}\right), \tag{53}$$

where $n = MN$ is the total length of the sequence, N is the number of blocks of M length.

Table 3. Aperiodic patterns for small values of m (1)

$m=2$	$m=3$	$m=4$	$m=5$	$m=6$	$m=7$	$m=8$
01	001	0001	00001	000001	0000001	00000001
10	011	0011	00011	000011	0000011	00000011
	100	0111	00101	000101	0000101	00000101
	110	1000	01011	000111	0000111	00000111
		1100	00111	001011	0001001	00001001
		1110	01111	001101	0001011	00001011
			11100	001111	0001101	00001101
			11010	010011	0001111	00001111
			10100	010111	0010011	00010011
			11000	011111	0010101	00010101
			10000	100000	0010111	00010111
			11110	101000	0011011	00011001
				101100	0011101	00011011
				110000	0011111	00011101
				110010	0100011	00011111
				110100	0100111	00100011
				111000	0101011	00100101
				111010	0101111	00100111
				111100	0110111	00101011
				111110	0111111	00101101
					1000000	00101111
					1001000	00110101
					1010000	00110111
					1010100	00111011
					1011000	00111101
					1011100	00111111
					1100000	01000011
					1100010	01000111
					1100100	01001011
					1101000	01001111
					1101010	01010011
					1101100	01010111
					1110000	01011011
					1110010	01011111
					1110100	01100111
					1110110	01101111
					1111000	01111111

For each j-th block ($j = 1, \ldots, N$), the statistic is calculated $W_j = W_j(m,M)$ of the occurrence of the pattern B. For each W_j, let $\mu = (M-m+1)\,2^{-m}$ and $\sigma^2 = M\left(\frac{1}{2^m} - \frac{2m-1}{2^{2m}}\right)$. If M is large enough, then W_j is normally distributed with mean value

Table 4. Aperiodic patterns for small values of m (2)

$m=2$	$m=3$	$m=4$	$m=5$	$m=6$	$m=7$	$m=8$
					1111010	10000000
					1111100	10010000
					1111110	10011000
						10100000
						10100100
						10101000
						10101100
						10110000
						10110100
						10111000
						10111100
						11000000
						11000010
						11000100
						11001000
						11001010
						11010000
						11010010
						11010100
						11011000
						11011010
						11011100
						11100000
						11100010
						11100100
						11100110
						11101000
						11101010
						11101100
						11110000
						11110010
						11110100
						11110110
						11111000
						11111010
						11111100
						11111110

μ and variance σ^2, so the statistic is approximately distributed χ^2 with N degrees of freedom and therefore,

$$\chi^2_{\text{obs}} = \sum_{j=1}^{N} \frac{(W_j - \mu)^2}{\sigma^2}. \tag{54}$$

The analyzed value of P has the form,

$$P\text{-value} = \text{igamc}\left(\frac{N}{2}, \frac{\chi^2_{\text{obs}}}{2}\right). \tag{55}$$

If the obtained value of P is less than the acceptable threshold (0.01) then the sequence should be considered non-random and characterized by irregular occurrences of possible patterns.

It is recommended to use patterns of length $m = 9$ or $m = 10$, the number of N sub-segments should be chosen so that $N \leq 100$ for the correct value of P a M selected to satisfy $M > 0.01n$ and $N = [n/M]$.

E.8 Test for Overlapping Patterns

Adapted from[6,22–24].

The test is used to identify non-random sequences that contain too many or not enough of a sequence of 1s of m length. The test can be modified to detect irregular occurrences of periodic B patterns.

The sequence in question, of length n, is divided into N blocks, each of length M, so that $n = MN$.

The number of runs of 1s of length m that can overlap in the j-th block is expressed as $\tilde{W}_j = \tilde{W}_j(m,M)$. While analyzing the behavior of the sequence of $\tilde{W}_j$ random variables, one can notice the convergence to the Poisson distribution of the distribution of these variables. If $(M - m + 1)\,2^{-m} \to \lambda > 0$, then for real t

$$Ee^{t\tilde{W}_j} \to e^{\frac{\lambda e^t - 1}{2 - e^t}}. \tag{56}$$

The probabilities related to the above scheme can be expressed using the confluent hypergeometric function $\Phi =_1 F_1$. Let U denotes some random variable of a complex asymptotic Poisson distribution, then for $u \leq 1$ and $\eta = \frac{\lambda}{2}$, the probability may be determined as:

$$P(U = u) = \frac{e^{-\eta}}{2^u} \sum_{l=1}^{u} \binom{u-1}{l-1} = \frac{\eta e^{-2\eta}}{2^u} \Phi(u+1, 2, \eta). \tag{57}$$

From the above, you can easily calculate the probabilities for small values of u, for example:

$$\begin{aligned} &P(U=0) = e^{-\eta}, \quad P(U=1) = \frac{\eta}{2} e^{-\eta}, \\ &P(U=2) = \frac{\eta}{8} e^{-\eta} (\eta + 2), \\ &P(U=3) = \frac{\eta}{8} e^{-\eta} \left(\frac{\eta^2}{6} + \eta + 1 \right), \\ &P(U=4) = \frac{\eta}{16} e^{-\eta} \left(\frac{\eta^3}{24} + \frac{\eta^2}{2} + \frac{3\eta}{2} + 1 \right). \end{aligned} \tag{58}$$

Probabilities can also be calculated as follows[24]:

- Consider a bit sequence of length $n-1$ that does not contain the m-bit pattern B. In case $n \leq (m-1)$, then the two sequences formed by appending 0 or 1 at the end to the leading sequence (both of length n) do not match the pattern B. If $n \geq m$ and the sequence of length $n-1$ contains m-bit pattern of the form $011\cdots11$ appended to the end of the sequence, then n pattern formed by appending 1 to the end of the sequence contains exactly one matching B pattern. The number of such sequences, if $T_0(n-m-1)$, is given by the recursive formula $T_0(n)$,

$$T_0(n) = \begin{cases} 1, & n = -1, \\ 1, & n = 0, \\ 2T_0(n-1), & 1 \leq n \leq m-1, \\ 2T_0(n-1) - T_0(n-m-1), & n \geq m. \end{cases} \tag{59}$$

- Consider a bit sequence of n that contains the pattern B exactly once. Such sequences have the pattern $011\cdots110$ and are long $m+2$. In case the pattern starts with j-th bit of the sequence, $T_0(j)$ patterns must be before this bit and $T_0(nm-2-j)$ patterns after this bit. Hence, the recursive formula for $T_1(n)$ takes the form,

$$T_1(n) = \begin{cases} 0, & n \leq m-1, \\ 1, & n = m, \\ 2, & n = m+1, \\ \sum_{j=-1}^{n-m-1} T_0(j) T_0(n-m-2-j), & n \geq m+2. \end{cases} \tag{60}$$

- Some of the binary sequences of length n in which the pattern B of length m occurred exactly α times can be formed by adding the bit 1 at the position of the first occurrence of a sequence of 1s in a binary sequence of length $n-1$, which has exactly the $\alpha-1$ occurrence of B; hence all the sequences obtained in this way will match the pattern B more than once at the first matching of 1s. The number of such sequences is $T_{\alpha-1}(n-1)$. The rest of the binary sequences of length n in which the pattern B of length m appeared exactly α times will match the pattern B only once in the first matching of 1's sequence. Let us consider a n bit sequence that has $(m+2)$ bit pattern $011\cdots110$ with exactly zero of the pattern B before the selected sequence and exactly $alpha-1$ of B pattern after the sequence. In the case when the pattern starts with j-th bit in the sequence, the number of such sequences is $T_0(j) T_{\alpha-1}(nm-2-j)$. Hence the recursive formula for $T_\alpha(n)$ is as follows,

$$\begin{aligned} T_\alpha(n) = &\sum_{j=1}^{n-(m+\alpha-2)} \\ &\times T_0(j-2) T_0(n-(j+m+\alpha-1)) \\ &+ \sum_{k=1}^{\alpha=1} \sum_{j=1}^{n-2m-\alpha+2} T_0(j-2) \\ &\times T_k(n-(j+m+\alpha-k-1)), \end{aligned} \tag{61}$$

where $\alpha \geq 2$.

- The above result can be shortened,

$$\begin{aligned} T_\alpha(n) &= T_{\alpha-1}(n-1) \\ &+ \sum_{j=-1}^{n-2m-\alpha} T_0(j)\, T_{\alpha-1}(n-m-2-j), \end{aligned} \tag{62}$$

which allows the probability calculation,

$$\pi_i = \frac{T_i(n)}{2^n}, i = 0,1,2,3,4, \quad \pi_5 = 1 - \sum_{i=0}^{4} \pi_i. \tag{63}$$

Probabilities π_i, for $i = 0, \ldots, 5$, calculated by application of both methods are presented in Table 5.

Table 5. Comparison of probabilities π_i which can be used to the test of the overlapping patterns

	values by NIST	values by[24]
π_0	0.367879	0.364091
π_1	0.183940	0.185659
π_2	0.137955	0.139381
π_3	0.099634	0.100571
π_4	0.069935	0.0704323
π_5	0.140657	0.139865

The complement of the distribution function of the analyzed random variable can be written as,

$$L(u) = P(U > u) = e^{-\eta} \sum_{l=1}^{u} \frac{\eta^l}{l!} \sum_{k=1}^{u} \frac{1}{2^k} \binom{k-1}{l-1}. \tag{64}$$

In order to define the statistics $K+1$ classes should be selected, for example,

$$\{U = 0\}, \{U = 1\}, \ldots, \{U = K-1\}, \{U \geq K\}$$

, and next the accompanying probabilities π_i (exemplary values: $K = 2, \lambda = 2, \eta = 1$).

For m-bit pattern B the frequency of ν_i, where ν_0 means no pattern, ν_1 means a single occurrence of the pattern, etc. should be calculated. The frequencies are computed by shifting the window by the size m bit by bit in the analyzed segment and counting the occurrences of 1's series of length m. After analyzing the entire episode, the total number of appearances allows to determine which of the grades/frequencies should be increased.

Using these frequencies and probabilities the statistics χ^2 can be expressed as,

$$\chi^2 = \sum_{i=0}^{K} \frac{(\nu_i - N\pi_i)^2}{N\pi_i}. \tag{65}$$

The analyzed P attains the form,

$$P = \text{igamc}\left(\frac{K}{2}, \frac{\chi^2_{\text{obs}}}{2}\right), \tag{66}$$

i.e., incomplete γ-function. If P is lower than the threshold (0.01), then the whole series is not random and exhibiting a non-regular appearance of some patterns.

It is suggested

- to choose K, M, N that each sequence has a minimal length 10^6,
- m should have value 9 lub 10,
- for other m it should be taken into account $n \geq MN$, $N(\min \pi_i) > K$, $\lambda = \frac{(M-m+1)}{2^m} \approx 2$, $m \approx \log_2 M$, $K \approx 2\lambda$.

E.9 Universal statistical Maurer test

Adapted from[6,25–30].

In 1992, Ueli Maurer (Princeton University) presented a statistical test based on the entropy of a bit in the stream, described by the author as a correct measure of the quality of a secret cryptographic key allowing the measurement of key randomness imperfection, expressed by the work time of the opponent's optimal key breaking strategy.

The author notes that this test is designed to detect some very general statistical imperfections that can be modeled using a stationary ergodic finite memory source. This test replaces several standard statistical tests.

The main intention is to detect whether the parsed sequence can be significantly compressed without losing information. If the sequence can be significantly compressed, then it is not truly random. This is based on Ziv's idea that a universal statistical test can be constructed using a universal source encoding algorithm. The Lempel-Ziv source coding algorithm is considered by Maurer to be less appropriate in the statistical approach due to the difficulty of defining a statistical test whose distribution could be analyzed.

The required length of the analyzed sequence for this test is quite large - $10 \cdot 2^L + 1000 \cdot 2^L, 6 \leq L \leq 16$. Such a sequence is divided into,

- two L-bit blocks, $6 \leq L \leq 16$,
- $Q \geq 10 \cdot 2^L$ initialization blocks, Q should be chosen to allow all L-bits to occur in the initialization block,
- $K \approx 1000 \cdot 2^L$ test blocks, $K = \lceil \frac{n}{L} \rceil - Q$.

Too large L values are not recommended due to test initiation time increasing exponentially with L.

The test scheme can be presented as follows:

- initialization sequence is parsed - traversing the sequence and parsing L-bit segments in test segments;
- while traversing, the test parses the entire sequence to find the closest previous occurrence of the L-bit pattern analyzed;
- distance between occurrences is recorded;
- algorithm calculates $\log_2$ of all saved L-bit patterns in the test segment;
- results are averaged over all selected block lengths.

There are defined,

$$f_n = \frac{1}{K} \sum_{i=Q+1}^{Q+K} \log_2 \left(i - T_j\left(i\right)\right), \tag{67}$$

where $T_j(i)$ is the value corresponding to the decimal representation of the contents of the i-th block of length L.

The algorithm uses dynamic table to perform this procedure efficiently. Relation,

$$\mathrm{E}\left(f_n\right) = 2^{-L} \sum_{i=1}^{\infty} \left(1 - 2^{-L}\right)^{i-1} \log_2 i, \tag{68}$$

where f_n is the test statistic with the expected value equal to the expected value of the random variable $\log_2 G_L$ and G_L is the geometric random variable with the parameter $1 - 2^{-L}$.

It is possible to estimate the empirical variance patterns in various ways, for example:

$$\mathrm{Var}\left(f_n\right) = c\left(L, K\right) \mathrm{Var}\left(\log_2 G\right) / K, \tag{69}$$

where $c(L,K)$ contains information about the occurrences of patterns in the parsed sequence.

The initial sequence is first split into $r \leq 20$ subsequences, and on each of the subsequences the value of the test stats is computed (for the same K, L, Q parameter values). The value of P can be expressed here as,

$$P = \mathrm{erfc}\left(\left|\frac{f_n - \mathrm{E}\left(L\right)}{\sqrt{\mathrm{Var}\left(f_n\right)}}\right|\right). \tag{70}$$

or, approximately,

$$P = \text{erfc}\left(\left|\frac{f_n - \text{E}(L)}{\sqrt{2)c\sqrt{\frac{\text{Var}\,L}{K}}}}\right|\right), \tag{71}$$

where $c = 0.7 - \frac{0.8}{L} + \left(4 + \frac{32}{L}\right)\frac{K^{-\frac{3}{L}}}{15}$.

It is recommended to test for sequences with a minimum length of n bits, where $n \geq (Q+K)L$.

If the resulting value of P-value is less than the acceptable threshold (0.01) then the sequence should be considered non-random.

E.10 Linear Complexity Test

Adapted from[6,27,28,31].

In this test, linear complexity is used to verify the randomness of the analyzed sequence. The idea is based on the so-called *Linear Feedback Shift Registers* (LFSR), which are L registers composed of L so-called *delay elements*, each with a single input and output. For example, if the initial LFSR state is $\varepsilon_{L-1}, \ldots, \varepsilon_1, \varepsilon_0$, then the output sequence is $\varepsilon_L, \varepsilon_{L-1}, \ldots, \varepsilon_1$, where

$$j \geq L \quad \varepsilon_j = \left(c_1\varepsilon_{j-1} + c_2\varepsilon_{j-2} + \ldots + c_L\varepsilon_{j-L}\right) \mod 2, \tag{72}$$

with the proviso that $c_1, \ldots, c_L$ are polynomial coefficients specific to the given LFSR. If a certain binary sequence is an LSFR output sequence for some initial state, it is said to be generated by the LFSR.

Linear complexity, $L(s^n)$, for a given sequence $s^n = (\varepsilon_1, \ldots, \varepsilon_n)$, is defined as the shortest LFSR that will generate s^n as its first n terms.

By using the Berlekamp-Massey algorithm it is possible to use linear complexity to analyze randomness.

For a truly random sequence of bits of length n, s^n, the mean and variance are defined as:

$$\begin{aligned}
&\text{E}(L(s^n)) = \tfrac{n}{2} + \tfrac{4+B(n)}{18} - \tfrac{1}{2^n}\left(\tfrac{n}{3} + \tfrac{2}{9}\right),\\
&\text{Var}(L(s^n)) = \sigma_n^2\\
&= \tfrac{86}{81} - \tfrac{1}{2^n}\left(\tfrac{14-B(n)}{27}n + \tfrac{82-2B(n)}{81}\right)\\
&-\tfrac{1}{2^{2n}}\left(\tfrac{1}{9}n^2 + \tfrac{4}{27}n + \tfrac{4}{81}\right).
\end{aligned} \tag{73}$$

Despite the suggestion in the Crypt-X package[27], the asymptotic distribution $\frac{(L_n - \mu_n)}{\sigma_n}$ of a sequence of even or odd values of n defined on the combination of two geometric random variables does not exist[6]. Hence, two cases have to be considered separately – for even and odd values of n, which results in two different distributions.

Due to the above circumstances, the following series of statistics is proposed:

$$\begin{aligned}
&T_n = (-1)^n(L_n - \xi_n) + \tfrac{2}{9},\\
&\xi_n = \tfrac{n}{2} + \tfrac{4+r_n}{18}.
\end{aligned} \tag{74}$$

The above statistics take only integer values and converge to the distribution of the T random variable.

$$\begin{aligned}
&P(T=0) = \frac{1}{2}, \quad k = 1,2,\ldots,\\
&P(T=k) = \frac{1}{2^{2k}},\\
&P(T=k) = \frac{1}{2^{2|k|+1}}, \quad k = -1,-2,\ldots,\\
&P(T \geq k > 0) = \frac{1}{3\cdot 2^{2k-2}},\\
&P(T \leq k) = \frac{1}{3\cdot 2^{2|k|-1}}.
\end{aligned} \tag{75}$$

Thus, they allow us to determine the value of P for the observed T_{obs}.

$$P = \frac{1}{3\cdot 2^{2\kappa-1}} + \frac{1}{3\cdot 2^{2\kappa-2}} = \frac{1}{2^{2\kappa-1}}. \tag{76}$$

Due to the fact that the distribution is not uniform for the value of P and is discrete, the parsed sequence must be of length $n = MN$, where N is the number of subsequences of length M. For each of N subscripts, the T_M statistic is computed and $K+1$ classes are matched, depending on M. For each of the analyzed subsequences, the frequencies $\nu_0, \nu_1, \ldots, \nu_K$ of T_M belonging to the appropriate class (one of $K+1$) are determined.

The probabilities $\pi_0, \pi_1, \ldots, \pi_K$ corresponding to $K+1$ classes are determined using $P(T = k)$ for $k = 1.2, \ldots$ and $k = -1, -2, \ldots$, for a large enough M ($500 < M < 5000$).

The χ^2 statistic with K degrees of freedom is produced, approximately,

$$\chi^2 = \sum_{i=0}^{K} \frac{(\nu_i - N\pi_i)^2}{N\pi_i}. \tag{77}$$

P is defined as follows,

$$\frac{\int_{\chi^2_{\text{obs}}}^{\infty} e^{-\frac{u}{2}} u^{\frac{K}{2}-1} du}{\Gamma\left(\frac{K}{2}\right) 2^{\frac{K}{2}}} = \text{igamc}\left(\frac{K}{2}, \frac{\chi^2_{\text{obs}}}{2}\right) \tag{78}$$

The condition for the above approximation is $N(\min \pi_i) \geq K$.

For recommended, large enough values of M and N, six classes with appropriate probability values can be proposed:

- $\{T \leq -2.5\}$, frequency ν_0, and probability $\pi_0 = 0.010417$,
- $\{-2.5 < T \leq -1.5\}$, frequency ν_1, and probability $\pi_0 = 0.03125$,
- $\{-1.5 < T \leq -0.5\}$, frequency ν_2, and probability $\pi_0 = 0.125$,
- $\{-0.5 < T \leq 0.5\}$, frequency ν_3, and probability $\pi_0 = 0.5$,
- $\{0.5 < T \leq 1.5\}$, frequency ν_4, and probability $\pi_0 = 0.25$,
- $\{1.5 < T \leq 2.5\}$, frequency ν_5, and probability $\pi_0 = 0.0625$,
- $\{T > 2.5\}$, frequency ν_6, and probability $\pi_0 = 0.020833$.

It is recommended to choose $n \geq 10^6$, $500 \leq M \leq 5000$, and $N \geq 200$ for the meaning of the approximation χ^2.

If the resulting value of P is less than the acceptable threshold (0.01) then the sequence should be considered non-random.

E.11 Serial test – pattern distribution test

Based on[3,6,28,32,33].

In this test, the initial sequence is analyzed using a series of procedures based on testing the uniformity of pattern distribution of a given length. The test evaluates the frequencies of all possible M overlapping patterns throughout the sequence and compares them with the results for a theoretically truly random sequence that is uniform – each pattern of a given length has the same probability of occurrence as any other pattern of the same length. In case $m = 1$, this test is the same as the frequency test described earlier.

Let the m-bit pattern be defined as $i_1, \ldots, i_m$, the starting sequence of n length is expanded with $m-1$ initial bits, which gives the so-called a circular sequence $\varepsilon_1, \ldots, \varepsilon_n, \varepsilon_1, \ldots, \varepsilon_{m-1}$, and $\nu_{i_1 \ldots i_m}$ is the frequency of the pattern $i_1, \ldots, i_m$ in an expanded sequence.

The ψ_m^2 statistic of type χ^2 is defined as follows,

$$\begin{aligned}
&\psi_m^2 = \tfrac{2^m}{n} \textstyle\sum_{i_1 \ldots i_m} \left(\nu_{i_1 \ldots i_m} - \tfrac{n}{2^m}\right)^2 = \tfrac{2^m}{n} \textstyle\sum_{i_1 \ldots i_m} \nu_{i_1 \ldots i_m}^2 - n, \\
&\psi_{m-1}^2 = \tfrac{2^{m-1}}{n} \textstyle\sum_{i_1 \ldots i_{m-1}} \left(\nu_{i_1 \ldots i_{m-1}} - \tfrac{n}{2^{m-1}}\right)^2 \\
&= \tfrac{2^{m-1}}{n} \textstyle\sum_{i_1 \ldots i_{m-1}} \nu_{i_1 \ldots i_{m-1}}^2 - n, \\
&\psi_{m-2}^2 = \tfrac{2^{m-2}}{n} \textstyle\sum_{i_1 \ldots i_{m-2}} \left(\nu_{i_1 \ldots i_{m-2}} - \tfrac{n}{2^{m-2}}\right)^2 \\
&= \tfrac{2^{m-2}}{n} \textstyle\sum_{i_1 \ldots i_{m-2}} \nu_{i_1 \ldots i_{m-2}}^2 - n,
\end{aligned} \tag{79}$$

but it does not have the χ^2 distribution as the $\nu_{i_1 \ldots i_m}$ frequencies are not independent.

This generalized serialized statistic can be written as,

$$\begin{aligned}\nabla\psi_m^2 &= \psi_m^2 - \psi_{m-1}^2 \\ \nabla^2\psi_m^2 &= \psi_m^2 - 2\psi_{m-1}^2 + \psi_{m-2}^2,\end{aligned} \tag{80}$$

where $\psi_0^2 = \psi_{-1}^2 = 0$.

Such a statistic, $\nabla\psi_m^2$ has a distribution of χ^2 with 2^{m-1} degrees of freedom, and $\nabla^2\psi_m^2$ has a distribution of χ^2 with 2^{m-2} degrees of freedom.

For the recommended $m \le \lfloor log_2(n) \rfloor - 2$ values of P have the form,

$$\begin{aligned}P_1 &= \text{igamc}\left(2^{m-2}, \frac{\nabla\psi_m^2}{2}\right), \\ P_2 &= \text{igamc}\left(2^{m-3}, \frac{\nabla^2\psi_m^2}{2}\right).\end{aligned} \tag{81}$$

If any of the P values are less than the acceptable threshold (0.01), consider the tested sequence non-random.

E.12 Entropy Estimation Test

Based on[6,34–36].

This test is also based on the analysis of repeating patterns in the starting sequence. All possible pattern overlaps along the entire sequence are analyzed over two blocks of successive lengths m and $m+1$ and then compared to the results expected for truly random sequences.

A pattern of length m is defined as,

$$Y_i(m) = (\varepsilon_i, \ldots, \varepsilon_{i+m-1}), \tag{82}$$

where $1 \le i \le n - m + 1$.

The relative frequency of $Y_i(m)$ occurrences in the analyzed sequence is:

$$\begin{aligned}&C_i^m = \tfrac{1}{n+1-m}\{number\ of\ such\ \text{j}\ that \\ &1 \le j < n-m, Y_j(m) = Y_i(m)\} = \pi_i.\end{aligned} \tag{83}$$

The entropy of the empirical distribution corresponding to all 2^m possible patterns of length m can be written as,

$$-\Phi^{(m)} = -\frac{1}{n+1-m}\sum_{i+1}^{n+1-m}\log C_i^m, \tag{84}$$

thus,

$$-\Phi^{(m)} = -\sum_{l=1}^{2^m}\pi_l \log \pi_l, \tag{85}$$

where π_l is relative frequency of the pattern j $l = i_1, \ldots, i_m$ in the sequence under analysis.

Let $H(m)$ correspond to the approximate entropy for $m \ge 1$, and let be defined as,

$$H(m) = \Phi^{(m)} - \Phi^{(m+1)}, \tag{86}$$

where $H(0) = -\Phi^{(m)}$. This approximated entropy is a measure of frequency that describes the situation where the m blocks that are close together remain close together when their lengths are incremented by one bit. The extreme values of this entropy indicate deviations from a truly random nature- - small values indicate strong sequence regularity, large values indicate fluctuations and irregularities.

In case the approximated $H(m)$ entropy has the greatest possible value, the corresponding sequence is called m–fluent or m-random[35]. Pincus and Kalman obtained interesting results while analyzing binary and decimal expansions of $e, \pi, \sqrt{2}, \sqrt{3}$ for $m = 1,2,3$ – $\sqrt{3}$ expansion has more regularity than the π expansion.

This test is based on the observation that for long random, therefore irregular sequences, and for a fixed length of m pattern, $H(m) \approx \log 2$. Rukhin showed the correlation between the boundary distribution of $n[\log 2 - H(m)]$ and the distribution of χ^2 for the variable with 2^m degrees of freedom.

This allows for the definition,

$$\chi^2_{\text{obs}} = n\left[\log 2 - H(m)\right],$$
$$P = \text{igamc}\left(2^{m-1}, \frac{\chi^2_{\text{obs}}}{2}\right). \tag{87}$$

It is possible to analyze this problem more precisely by modifying the definition of approximated entropy.

Using the relative frequency formula $\nu_{i_1\ldots i_m} = \frac{\omega_{i_1\ldots i_m}}{n}$ pattern $i_1 \ldots i_m$ in an expanded sequence, $\varepsilon_1,\ldots,\varepsilon_n,\varepsilon_1,\ldots,\varepsilon_{m-1}$, you can write,

$$\tilde{\Phi}^{(m)} = \sum_{i_1\ldots i_m} \nu_{i_1\ldots i_m} \log \nu_{i_1\ldots i_m}. \tag{88}$$

So $\omega_{i_1\ldots i_m} = \sum_k \omega_{i_1\ldots i_m k}$ comes down to $\sum_{i_1\ldots i_m} \omega_{i_1\ldots i_m} = n$ for any m .

The modified approximated entropy is defined as:

$$\tilde{H}(m) = \tilde{\Phi}^{(m)} - \tilde{\Phi}^{(m+1)}. \tag{89}$$

Since $\log s \geq \tilde{H}(m)$ for any m, from Jensen's equation, it is possible that $\log s < \tilde{H}(m)$, which results in the largest possible value of $H(m) = \log s$ and is achieved when $n = s^m$ and for a uniform distribution of patterns of length m .

For large n, the approximated entropy and the modified approximated entropy do not differ much.

$$\nu^{'}_{i_1\ldots i_m} = \frac{\omega^{'}_{i_1\ldots i_m}}{n-m+1},$$
$$\sum_{i_1\ldots i_m} \omega^{'}_{i_1\ldots i_m} = n-m+1,$$
$$\omega_{i_1\ldots i_m} - \omega^{'}_{i_1\ldots i_m} \leq m-1,$$
$$\Rightarrow \left|\nu_{i_1\ldots i_m} - \nu^{'}_{i_1\ldots i_m}\right| \leq \frac{m-1}{n-m+1}. \tag{90}$$

This leads to the proximity of the approximated entropy and the modified approximated entropy – finally to the consistency of their asymptotic distributions.

If the resulting value of P is less than the acceptable threshold (0.01) then the sequence should be considered non-random.

It is recommended to choose m and n so that $m < \lfloor \log_2 n \rfloor - 5$.

E.13 Test of cumulative (rising) sums

Adapted from[6, 15, 37].

In this test, the initial zero-one sequence is converted to a sequence of ones and minus ones (zero transformations), then analyzed for the largest integer subtotal values. If the values are large, it means too ma 1s and zeros (minus 1s after conversion) in the initial stages of the analyzed sequence, on the other hand, if the values are very small, it indicates too even distribution (mixing) of 1s and 0s.

It is possible to get a similar test by using *reversed time random walk* for $S^{'}_k = X_n + \ldots + X_{n-k+1}$, with one difference – the results corresponding to the initial stages will now correspond to the final stages of the analyzed sequence.

The statistical concept of this test uses the asymptotic distribution of the maximum values of the subtotals, $\max\limits_{1\leq k\leq n} |S_k|$, what can be written as

$$\lim_{n\to\infty} P\left(\frac{\max\limits_{1\leq k\leq n}|S_k|}{\sqrt{n}} \leq z\right)$$
$$= \frac{1}{\sqrt{2n}} \int_{k=-\infty}^{\infty} (-1)^k e^{-\frac{(u-2kz)^2}{2}} du$$
$$= \frac{4}{\pi} \sum_{j=0}^{\infty} \frac{(-1)^j}{2j+1} e^{-\frac{(2j+1)^2\pi^2}{8z^2}} = H(z), \tag{91}$$

where $z > 0$. If $z = \frac{\max\limits_{1\leq k\leq n}|S_k|_{\text{obs}}}{\sqrt{n}}$ is high then such a sequence is rejected as non-random.

The value of P for the above statistic is defined as

$$\begin{aligned}P &= 1-H\left(\frac{\max\limits_{1\le k\le n}|S_k|_{\text{obs}}}{\sqrt{n}}\right)\\ &= 1-G\left(\frac{\max\limits_{1\le k\le n}|S_k|_{\text{obs}}}{\sqrt{n}}\right),\end{aligned} \tag{92}$$

where $G(z)$ is defined below.

Since $H(z)$ runs quickly, it is convenient to use $G(z)$ instead of $H(z)$ for medium and large z and functions $H(z)$ for small values. $G(z)$ takes the form,

$$\begin{aligned}G(z) &= \tfrac{1}{\sqrt{2\pi}}\int_{-z}^{z}\sum_{k=-\infty}^{\infty}(-1)^k e^{-\frac{(u-2kz)^2}{2}}du\\ &= \sum_{k=-\infty}^{\infty}(-1)^k(\Phi((2k+1)z)-\Phi((2k-1)z))\\ &= \Phi(z)-\Phi(-z)\\ &+2\sum_{k=1}^{\infty}(-1)^k(\Phi((2k+1)z)-\Phi((2k-1)z))\\ &= \Phi(z)-\Phi(-z)\\ &-2\sum_{k=1}^{\infty}(2\Phi((4k-1)z)-\Phi((4k+1)z)-\Phi((4k-3)z))\\ &\approx \Phi(z)-\Phi(-z)\\ &-2(2\Phi(3z)-\Phi(5z)-\Phi(z)) \approx 1-\tfrac{4}{\sqrt{2\pi}z}e^{-\frac{z^2}{2}}, \quad z\to\infty,\end{aligned} \tag{93}$$

where $\Phi(x)$ is the normal distribution.

Using the theorem[15]

Theorem: For any integer $a \le 0 \le b$ and $a \le u \le \nu \le b$, we have,

$$\begin{aligned}&P(a < -M_n^- \le M_n^+ < b, u < S_n < \nu)\\ &= \sum_{k=-\infty}^{\infty}P(u+2k(b-a) < S_n < \nu+2k(b-a))\\ &-\sum_{k=-\infty}^{\infty}P(2b-\nu+2k(b-a) < S_n < 2b-u+2k(b-a)),\end{aligned} \tag{94}$$

$$\begin{aligned}&P(a < -M_n^- \le M_n^+ < b) =\\ &\sum_{k=-\infty}^{\infty}P(a+2k(b-a) < S_n < b+2k(b-a))\\ &-\sum_{k=-\infty}^{\infty}P(b+2k(b-a) < S_n < 2b-a+2k(b-a)),\end{aligned} \tag{95}$$

$$\begin{aligned}P(M_n < b) &= \sum_{k=-\infty}^{\infty}P((4k-1)b < S_n < (4k+1)b)\\ &-\sum_{k=-\infty}^{\infty}P((4k+1)b < S_n < (4k+3)b),\end{aligned} \tag{96}$$

one can obtain,

$$\begin{aligned}&P\left(\max_{1\le k\le n}|S_n| \ge z\right)\\ &= 1-\sum_{k=-\infty}^{\infty}P((4k-1)z < S_n < (4k+1)z)\\ &+\sum_{k=-\infty}^{\infty}P((4k+1)z < S_n < (4k+3)z).\end{aligned} \tag{97}$$

The above theorem is used to calculate the correct P value for this test from $z = \frac{\max\limits_{1\le k\le n}|S_{k\,\text{obs}}|}{\sqrt{n}}$.

If the resulting value of P is less than the acceptable threshold (0.01) then the sequence should be considered non-random.

It is recommended that $n \le 100$.

E.14 Random excursion test

Adapted from[6,15,37,38].

The test analyzes the distribution of the number of visits to a state as a result of a trip or a simple random 'walk' - the initial sequence is transformed to contain only 1s and minus 1s (not zeros); successive sums of bits are considered.

Let $S_k = X_1 + \ldots + X_k$ denote a simple random walk, where X_i are independent variables taking the values ± 1 with the probabilities p and $1 - p = q$ respectively.

For $S_0 = 0$ let $\rho_1 < \rho_2 < \ldots$ denote the times when the walk start is reached,

$$\begin{aligned} &\rho_1 = \min\{k, k > 0, S_k = 0\}, \\ &\rho_2 = \min\{k, k > \rho_1, S_k = 0\}, \\ &\ldots. \end{aligned} \tag{98}$$

Within this random walk, a series of trips to and from zero can be folded in the form,

$$\left(S_0, \ldots, S_{\rho_1}\right), \left(S_{\rho_1}, \ldots, S_{\rho_2}\right), \ldots, \tag{99}$$

or

$$(i, \ldots, l) : S_{i-1} = S_{i+1} = 0, \quad S_k \neq 0, \quad i \leq k \leq l. \tag{100}$$

If J is the total number of such trips in the analyzed sequence – therefore it is a random variable – then its asymptotic distribution will be in the form,

$$\lim_{n\to\infty} P\left(\frac{J}{\sqrt{n}} < z\right) = \sqrt{\frac{2}{\pi}} \int_0^z e^{-\frac{u^2}{2}} du, \quad z > 0, \tag{101}$$

and the associated value of P can be written as,

$$P(J < J_{\text{obs}}) \approx \sqrt{\frac{2}{\pi}} \sum_0^J e^{-\frac{u^2}{2}} du = P\left(\frac{1}{2}, \frac{J_{\text{obs}}^2}{2n}\right). \tag{102}$$

For small values of J, $J < \max\{0.005\sqrt{n}, 500\}$, the parsed sequence is considered non-random. For $J \geq \max\{0.005\sqrt{n}, 500\}$, the number of random walk visits in a given state is calculated.

Let $\xi(x)$ be the number of visits of the $x \neq 0$ state during the first single trip. Its distribution can be obtained as follows.

For $\xi(x) > 0$, $\xi(x)$ has a geometric distribution with the parameter

$$\pi = \begin{cases} \frac{|p-q|}{1-\left(\frac{q}{p}\right)^x}, & p > q, x > 0 \text{ or } p < q, x < 0, \\ \frac{|p-q|}{1-\left(\frac{p}{q}\right)^x}, & p > q, x < 0 \text{ or } p < q, x > 0, \end{cases} \tag{103}$$

where $P(\xi(x) = 0) = 1 - \frac{|p-q|}{\left|1-\left(\frac{p}{q}\right)^x\right|}$.

So $\xi(x)$ has a geometric distribution with probabilities,

$$\begin{aligned} &P(\xi(x) = 0) = p_0, \\ &P(\xi(x) = k) = (1 - p_0)\pi(1 - \pi)^{k-1}, \quad k = 1, 2, \ldots, \end{aligned} \tag{104}$$

which gives,

$$\begin{aligned} E(\xi(x)) &= \frac{1 - p_0}{\pi}, \\ \text{Var}(\xi(x)) &= \frac{(1 - p_0)(1 - \pi + p_0)}{\pi^2}, \end{aligned} \tag{105}$$

and for $p = \frac{1}{2}$

$$\begin{aligned} &E(\xi(x)) = 1, \\ &\text{Var}(\xi(x)) = 4|x| - 2. \end{aligned} \tag{106}$$

For $a = 0, 1, 2, \ldots$ one can write,

$$\begin{aligned} &P(\xi(x) > a) = (1 - p_0)(1 - \pi)^a \\ &= \frac{P(\xi(x)=a+1)}{\pi}, \\ &p = \tfrac{1}{2} \to P(\xi(x) > a) = \tfrac{1}{2|x|}\left(1 - \tfrac{1}{2|x|}\right)^a \\ &= 2|x| P(\xi(x) = a + 1). \end{aligned} \tag{107}$$

The results allow the bit sequence randomness to be tested as follows,

- For a given collection of states, x values (for example, $-4 \leq x \leq 4, x \neq 0$ or $-7 \leq x \leq 7, x \neq 0$), observed frequencies k state visits x during J tours, $\nu_k(x)$, are computed,

$$\nu_k(x) = \sum_{j=1}^{J} \nu_k^j(x), \tag{108}$$

where $\nu_k^j(x) = 1$ if x is visited on j-th tour, $j = 1, \ldots, J$, exactly k times, and $\nu_k^j(x) = 0$ otherwise.

- $\xi(x)$ values are split into classes with different k, such as $k = 0.1, \ldots, 4$, and $k \geq 5$.
- For the above probability classes, they are computed as:

$$\begin{aligned} \pi_0 &= P(\xi(x) = 0) = 1 - \frac{1}{2|x|}, \\ \pi_k &= P(\xi(x) = k) = \frac{1}{4x^2}\left(1 - \frac{1}{2|x|}\right)^{k-1}, k = 1, \ldots, 4, \\ \pi_5 &= P(\xi(x) \geq 5) = \frac{1}{2|x|}\left(1 - \frac{1}{2|x|}\right)^4. \end{aligned} \tag{109}$$

For arbitrary x,

$$\chi^2(x) = \sum_{k=0}^{5} \frac{(\nu_k(x) - J\pi_k(x))^2}{J\pi_k(x)}, \tag{110}$$

should have an approximated distribution χ^2 with 5 degrees of freedom, where $J \min \pi_k(x) \geq 5$, i.e., $J \geq 500$ – if not, then greater a set of classes should be considered.

P values corresponding to the above can be written as,

$$1 - P\left(\frac{5}{2}, \frac{\chi^2_{\text{obs}}(x)}{2}\right). \tag{111}$$

The entire procedure can be presented as the following steps,

1. The parsed sequence is $\varepsilon_0\varepsilon_1 \ldots \varepsilon_n$.
2. The sequence is converted to X, $X_i = 2\varepsilon_i - 1$.
3. The set $S = \{S_i\}$ is computed, where S_i is the partial sum, $S_i = \sum_{k=1}^{i} X_k$.
4. S' is created by substituting S with $S_0 = 0$ at the beginning and appending S with $S_n = 0$ to the end of the set.
5. This generates a random walk
6. J is the integer of zeros in S' without a leading zero – J is actually the number of cycles in S'.
7. For each cycle and for each x value, without zero, for example $-p \leq x \leq -1$ and $1 \leq x \leq p$, where p is the test parameter, the frequency of each x is computed every cycle.
8. For each state of x the appropriate frequencies are stored in $\nu_k(x)$ – this is equal to the number of cycles in which the given state x occurs exactly k times, frequencies greater than k is stored in $\nu_k(x)$. $\sum_{l=0}^{k} \nu_l(x) = J$.
9. For each x state, the statistic is calculated from the relation,

$$\chi^2(x) = \sum_{l=0}^{k} \frac{(\nu_l(x) - J\pi_l(x))^2}{J\pi_l(x)}, \tag{112}$$

where probabilities π_l are calculated as shown above.

10. Finally, the value of P is computed for each state as,

$$P = \text{igamc}\left(\frac{k}{2}, \frac{\chi^2_{\text{obs}}}{2}\right). \tag{113}$$

If the resulting value of P is less than the acceptable threshold (0.01) then the sequence should be considered non-random.

It is recommended that the parsed sequence be of length $n \geq 10^6$ and $J \min \pi_k(x) \geq k$.

E.15 Variant random excursion test

Adapted from[6,15,37,38].

Using the definition of the Random Tour Test, you can create a variant of it.

Using $\xi(x)$ – the number of x state visits in the first single trip – let $\xi_J(x)$ be the total number of x state visits during J tours. Since S_k refreshes every occurrence of 0 in the trip, $\xi_J(x)$ will be the sum of independent variables identical to $\xi(x) = \xi_1(x)$, which gives an asymptotic distribution, $\xi_J(x)$

$$\lim_{J\to\infty} P\left(\frac{\xi_J(x) - J}{\sqrt{J(4|x|-2)}} < z\right) = \Phi(z), \tag{114}$$

which in this case is the normal distribution. Thus, the corresponding value of P has the form,

$$P = \text{erfc}\left(\frac{|\xi_{J\text{obs}}(x) - J|}{\sqrt{2J(4|x|-2)}}\right). \tag{115}$$

F New prototype of QRNG upon the project Jurand, JUR02

The small QRNG has been presented in 2020 upon the project NCBiR, POIR.01.01.01-00-0173/15. The prototype called JUR02 is miniaturized to the box of size $28\times10\times46.5$ mm and integrated with conventional USB port allowing for universal easy application in personal computers – cf. Fig. 1.

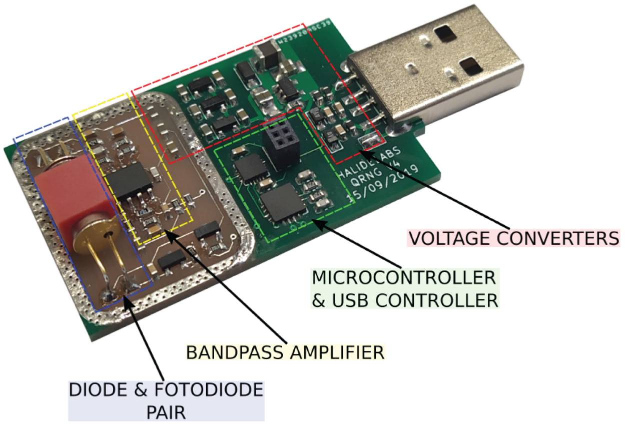

Figure 1. Prototype of miniaturized version of QRNG JUR02 designed at WUST (2020) – it passed all NIST/Dieharder tests without the bias reduction, at the speed of entropy creation 1 Mb/s (configured with conventional USB controller).

JUR02 successfully passed NIST SP-800-22 and Dieharder v. 3.31.1 tests. Testing using the package Linux-ent gives

- Optimum compression would reduce the size of this 2064385896 byte file by 0%
- Chi square distribution for 2064385896 samples is 255.90, and randomly would exceed this value by 47.23%
- Arithmetic mean value of data bytes is 127.5014 (127.5 = random)
- Monte Carlo value for π is 3.141579925 (error 0.00%)
- Serial correlation coefficient is -0.000035 (totally uncorrelated = 0.0)

The source of randomness in JUR02 is the shot noise in the photodiode and produces the random bit sequence at the time rate 1 Mb/s. The simplified block-scheme of JUR02 is shown in Fig. 2 and the scheme of starting sequence and the algorithm of random bit generation is visualized in Fig. 3. The exemplary data (2GB) from JUR02 are available at address https://halidelabs.eu/QRNG/data.bin.

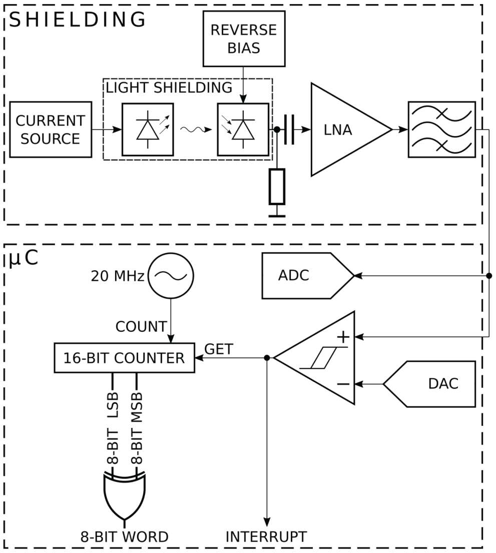

Figure 2. Simplified block scheme of JUR02.

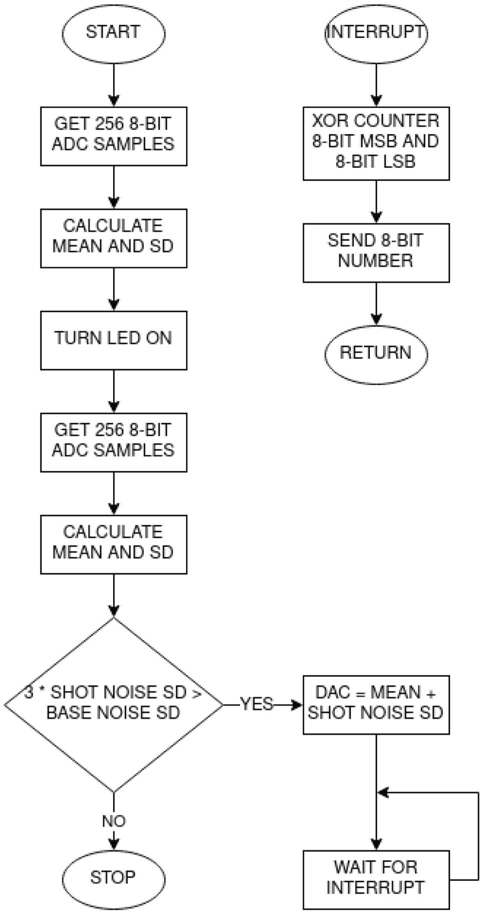

Figure 3. The scheme of the algorithm for automatic starting and calibration sequence and generating of random bit sequence implemented in JUR02.

References

1. Khrennikov, A. Randomness: quantum versus classical. *Int. J. Quantum Inform.* **14**, 1640009 (2016).
2. Marangon, D. G. *et al.* Long-term test of a fast and compact quantum random number generator. *J. Light. Technol.* **36**, 3778 (2018).
3. Knuth, D. E. *The Art of Computer Programming. Vol 2: Seminumerical Algorithms. 3rd ed.* (Addison-Wesley, Reading, 1998).
4. Marsaglia, G. *Diehard: a battery of tests of randomness* (1996). [Online]. Avaliable: http://stat.fsu.edu/pub/diehard/. Accessed on: January 5, 2021.
5. Brown, R. G. *Dieharder* (2006). [Online]. Avaliable: https://webhome.phy.duke.edu/~rgb/General/dieharder.php. Accessed on: January 5, 2021.
6. Rukhin, A. *et al.* A statistical test suite for random and pseudorandom number generators for cryptographic applications. *NIST Special Publ. 800-22 Revis. 1a* (2010). Revisor: Lawrence E. Bassham III.
7. Chung, K. L. *Elementary Probability Theory with Stochastic Processes* (Springer Verlag, New York, 1979).
8. Pitman, J. *Probability* (Springer Verlag, New York, 1993).
9. Rice, J. A. *Mathematical Statistics and Data Analysis (Second ed.)* (Duxbury Press, Belmont, 1995).
10. Maclaren, N. *Cryptographic Pseudo-random Numbers in Simulation. Cambridge Security Workshop on Fast Software Encryption* (R. Anderson, Cambridge, 1993).
11. Abramowitz, M. & Stegun, I. *Handbook of Mathematical Functions: NBS Applied Mathematics Series 55* (U.S. Government Printing Office, Washington, 1967).
12. Gibbons, J. D. *Nonparametric Statistical Inference, 2nd ed.* (Marcel Dekker, New York, 1985).
13. Godbole, A. P. & Papastavridis, S. G. *Runs and patterns in probability: Selected papers* (Kluwer Academic, Dordrecht, 1994).
14. David, F. N. & Barton, D. E. *Combinatorial Chance* (Hafner Publishing Co., New York, 1962).
15. Revesz, P. *Random Walk in Random and Non-Random Environments* (World Scientific, Singapore, 1990).
16. Kovalenko, I. N. Distribution of the linear rank of a random matrix. *Theory Probab. its Appl.* **17**, 342 (1972).
17. Marsaglia, G. & Tsay, L. H. Matrices and the structure of random number sequences. *Linear Algebr. its Appl.* **67**, 147 (1985).
18. Bracewell, R. N. *The Fourier Transform and Its Applications* (McGraw-Hill, New York, 1986).
19. Killman, W., Schüth, J., Thumser, W. & Uludag, I. A note concerning the dft test in nist special publication 800-22. *T-Systems, Syst. Integration* (2004).
20. Kim, S., Umeno, K. & Hasegawa, A. Corrections of the nist statistical test suite for randomness. *Cryptol. ePrint Arch. Rep. 2004/018* (2004).
21. Barbour, A. D., Holst, L. & Janson, S. *Poisson Approximation* (Clarendon Press, Oxford, 1992).
22. Chrysaphinou, O. & Papastavridis, S. A limit theorem on the number of overlapping appearances of a pattern in a sequence of independent trials. *Probab. Theory Relat. Fields* **79**, 129 (1988).
23. Johnson, N. J., Kotz, S. & Kemp, A. *Discrete Distributions. 2nd ed.* (John Wiley, New York, 1996).
24. Hamano, K. & Kaneko, T. The correction of the overlapping template matching test included in nist randomness test suite. *IEICE Transactions Electron. Commun. Comput. Sci.* **E90-A**, 1788 (2007).
25. Maurer, U. M. A universal statistical test for random bit generators. *J. Cryptol.* **5**, 89 (1992).
26. Coron, J.-S. & Naccache, D. *An Accurate Evaluation of Maurer's Universal Test. Proceedings of SAC '98 (Lecture Notes in Computer Science)* (Springer Verlag, Berlin, 1998).
27. Gustafson, H., Dawson, E., Nielsen, L. & Caelli, W. A computer package for measuring the strength of encryption algorithms. *Computers @AND@ Security* **13**, 687 (1994).
28. Menezes, A. J., van Oorschot, P. C. & Vanstone, S. A. *Handbook of Applied Cryptography* (CRC Press, Boca Raton, 1997).
29. Ziv, J. *Compression, tests for randomness and estimating the statistical model of an individual sequence. Sequences (ed. R.M. Capocelli)* (Springer Verlag, Berlin, 1990).

30. Ziv, J. & Lempel, A. A universal algorithm for sequential data compression. *IEEE Transactions on Inf. Theory* **23**, 337 (1977).

31. Rueppel, R. A. *Analysis and Design of Stream Ciphers* (Springer Verlag, New York, 1986).

32. Ziv, J. & Lempel, A. The serial test for sampling numbers and other tests for randomness. *Proc. Camb. Philos. Soc.* **47**, 276 (1953).

33. Ziv, J. & Lempel, A. Comparison of two statistical tests for keystream sequences. *Electron. Lett.* **23**, 365 (1987).

34. Pincus, S. & Singer, B. H. Randomness and degrees of irregularity. *Proc. Natl. Acad. Sci. USA* **93**, 2083 (1996).

35. Pincus, S. & Kalman, R. E. Not all (possibly) "random" sequences are created equal. *Proc. Natl. Acad. Sci. USA* **94**, 3513 (1997).

36. Rukhin, A. Approximate entropy for testing randomness. *J. Appl. Probab.* **37**, 88 (2000).

37. Spitzer, F. *Principles of Random Walk* (Van Nostrand, Princeton, 1964).

38. Baron, M. & Rukhin, A. L. Distribution of the number of visits for a random walk. *Commun. Stat. Stoch. Model.* **15**, 593 (1999).

39. Nielsen, M. & Chuang, I. *Quantum Computation and Quantum Information* (Cambridge UP, Cambridge, 2010).

40. Landau, L. & Lifschitz, L. *Machanika kwantowa, teoria nierelatywistyczna* (PWN, 2012).

Acknowledgements

Supported by the Polish National Centre for Research and Development project POIR.01.01.01-00-0173/15 Jurand
